# Reporting individual results for biomonitoring and environmental exposures: lessons learned from environmental communication case studies

**DOI:** 10.1186/1476-069X-13-40

**Published:** 2014-05-26

**Authors:** Julia Green Brody, Sarah C Dunagan, Rachel Morello-Frosch, Phil Brown, Sharyle Patton, Ruthann A Rudel

**Affiliations:** 1 Silent Spring Institute, Newton, MA, USA; 2 School of Public Health and Department of Environmental Science, Policy and Management, University of California, Berkeley, Berkeley, CA, USA; 3 Department of Sociology and Anthropology and Department of Health Sciences, Northeastern University, Boston, MA, USA; 4 Commonweal, Bolinas, CA, USA

**Keywords:** Bioethics, Biomonitoring, Community-based participatory research, Exposure assessment, Health literacy, Informed consent, Research ethics, Risk communication

## Abstract

Measurement methods for chemicals in biological and personal environmental samples have expanded rapidly and become a cornerstone of health studies and public health surveillance. These measurements raise questions about whether and how to report individual results to study participants, particularly when health effects and exposure reduction strategies are uncertain. In an era of greater public participation and open disclosure in science, researchers and institutional review boards (IRBs) need new guidance on changing norms and best practices. Drawing on the experiences of researchers, IRBs, and study participants, we discuss ethical frameworks, effective methods, and outcomes in studies that have reported personal results for a wide range of environmental chemicals. Belmont Report principles and community-based participatory research ethics imply responsibilities to report individual results, and several recent biomonitoring guidance documents call for individual reports. Meaningful report-back includes contextual information about health implications and exposure reduction strategies. Both narrative and graphs are helpful. Graphs comparing an individual’s results with other participants in the study and benchmarks, such as the National Exposure Report, are helpful, but must be used carefully to avoid incorrect inferences that higher results are necessarily harmful or lower results are safe. Methods can be tailored for specific settings by involving participants and community members in planning. Participants and researchers who have participated in report-back identified benefits: increasing trust in science, retention in cohort studies, environmental health literacy, individual and community empowerment, and motivation to reduce exposures. Researchers as well as participants gained unexpected insights into the characteristics and sources of environmental contamination. Participants are almost universally eager to receive their results and do not regret getting them. Ethical considerations and empirical experience both support study participants’ right to know their own results if they choose, so report-back should become the norm in studies that measure personal exposures. Recent studies provide models that are compiled in a handbook to help research partnerships that are planning report-back. Thoughtful report-back can strengthen research experiences for investigators and participants and expand the translation of environmental health research in communities.

## Background

In 1999, Cape Cod, MA, women generously opened their doors to Silent Spring Institute researchers to conduct a study of household exposures to 89 endocrine disrupting compounds [1]. The participants in the Household Exposure Study offered tea and shared information about their personal history and product use, while researchers collected air and dust samples and retrieved jars of urine samples. Shortly thereafter, study participants began calling to ask what the researchers had found and if it was safe. Their questions raised difficult issues about whether and how to report individual results. For some chemicals, these were the first measurements ever reported in homes, so researchers didn’t know what levels were typical, and, although the chemicals were known to be biologically active in the lab, the human health effects of low-level exposure had not been evaluated. At the time, research protocols typically called for reporting individual results only if they were clinically relevant, such as lead levels above an action level, but after considering ethical guidelines and consulting community members, we concluded that study participants had a right to decide whether or not they wanted to receive their own results.

Over the last several years, other researchers have similarly offered participants their own results, and this practice is slowly becoming the norm rather than the exception. Major guidance documents, described in Table 1, have called for report-back, too. The California Biomonitoring Program, established in 2006, is required by law to make individual results available [2], and European [3,4] and Canadian [5] biomonitoring programs also offer individual results. The National Academy of Sciences 2006 report on biomonitoring [6], the Centers for Disease Control National Conversation on Public Health and Chemical Exposures [7], and the federal Interagency Breast Cancer and the Environment Coordinating Committee [8] further identify the need to develop and test best practices. Parallel discussions about genetics results and patient access to medical records [9] offer partially relevant models for communicating uncertain data.

**Table 1:** Guidance on reporting personal exposure results

**Agency/Document**	**Text**
National Academy of Sciences, Human Biomonitoring for Environmental Chemicals, 2006	“…the committee considers that subjects should be told (or offered the chance to be told) whatever researchers know (or do not know)” (p. 73) [6]
	“Effective communication of results is among the biggest challenges to the future of biomonitoring…Recommendation: Advance individual, community, and population-based strategies for reporting results of biomonitoring studies.” (p.182) [6]
State of California, California Environmental Contaminant Biomonitoring Program (SB1379), 2006	“Individuals may request and shall receive their complete results.” (Section 2, 105443 (a)) [2]
Expert team to Support BIOmonitoring in Europe (ESBIO), Development of a coherent approach to human biomonitoring in Europe, 2007	“The possibility of reporting personal results to the participants … should have particular attention in order to enhance the benefits for study participants and to raise response and commitment in return.” (p.22) [3]
	“The individual pollutant concentrations of mother and child should be reported to the mother together with an evaluation of the results.” (p. 23) [3]
Statistics Canada, Canadian Health Measures Survey: Ethical, legal and social issues, 2007	“In accordance with ethical, legal and social principles, any information collected about a person should be provided to that person if requested.” (pp. 44–5) [5]
Environmental Protection Agency, Scientific and Ethical Approaches for Observational Exposure Studies, 2008	“Researchers need to develop the approach for reporting results to the participants, community, stakeholders, media, and others during the initial planning of the study.” (p. 87) [10]
Boston Consensus Conference on Human Biomonitoring, reported by Nelson et al. 2009	“…the group asserts that study participants should be able to decide whether or not they want to receive their personal results, and that an important element of this report be inclusion of action steps for reducing exposure, when these are available.” (pp. 497–8) [11]
National Conversation Leadership Council, National Conversation on Public Health and Chemical Exposures, 2011	“Recommendation 5.5: Increase public access to data by…ensuring that respondents have access to data collected on them…Study respondents should be offered the option to receive the results of health examinations and clinical tests, including biomonitoring and physical samples collected from their property. These data should be accompanied by an explanation in lay terms that provides context for the exposure measurements.” (p. 58) [7]
Interagency Breast Cancer and the Environment Coordinating Committee, Breast Cancer and the Environment: Prioritizing Prevention, 2013	“The growing consensus is that policies are needed to guide researchers in reporting study results back to participants…Researchers repeatedly have highlighted the ethical need to report back exposure information to research participants.” (p. 8–8) [8]
Consortium to Perform Human biomonitoring on a European Scale (COPHES), A systematic approach for designing a HBM Pilot Study for Europe, reported by Becker et al. 2014	“For DEMOCOPHES, in most countries, the participating mother received a letter with individual results of the chemical analyses…and mothers could indicate the wish not to receive results.” (p. 318) [4]
	“The procedures for reporting personal results to the participants…required particular attention in order to enhance the value for study participants and to raise response and commitment in return.” (p. 319) [4]

To develop best practices specifically for environmental health, we began a program of study and consultation to learn about the experiences of participants, researchers, and institutional review board (IRB) representatives in personal exposure studies. In this commentary we discuss ethical frameworks, effective methods, and lessons learned, so that researchers and IRBs can confidently expand report-back in environmental health studies. In addition to peer-reviewed literature, we draw on our own exposure studies, interviews with participants, researchers, and IRB members and staff in these and six other studies, and discussions among 44 participants in a 2010 workshop that brought together researchers, IRB representatives, ethicists, lawyers, public health officials, physicians, and activists. (The agenda and participants are shown in Additional files 1 and 2.) The interviews and workshop are part of the Personal Exposure Report-back Ethics (PERE) Study, and protocols were approved by IRBs at Northeastern and Brown universities.

This commentary contributes to improved exposure science by discussing strategies that encourage participation and retention by “giving back” to participants and showing them they are respected [12,13]. We also contribute to environmental health literacy by describing report-back methods that help people understand chemical exposures. A more detailed handbook, entitled, When Pollution is Personal: Best Practices for Reporting Results to Participants in Biomonitoring and Personal Exposure Studies (hereafter referred to as “Report-back Handbook”), includes additional examples (see Additional file 3) [14].

## Discussion

As more teams experiment with report-back, their experiences create a track record to inform ethical decisions and best practice methods.

### Ethics: weighing potential benefits as well as harms

As a starting point for ethical considerations, the 1979 Belmont Report, which established the basic ethical framework for modern biomedical research in the U.S., calls on researchers to avoid harm and maximize beneficence, autonomy, and justice [15]. These standards have sometimes been interpreted to weigh against reporting for emerging contaminants, because of concerns that participants cannot benefit if results have uncertain clinical health implications and might be harmed by excessive worry about their exposures. However, in environmental public health, the potential benefits often occur outside of clinical care, and decisions rely on animal and limited epidemiologic evidence, because these are the best resources available. In this context, beneficence can encompass giving participants opportunities to learn about the strengths and weaknesses of the science in order to make their own decisions about their results, and autonomy and justice also reinforce the participant’s right-to-know their results in order to act on them [16]. For example, participants may choose to reduce exposures as a precaution or to become engaged in public discourse about chemical use and regulation. From an evidence-based perspective, although researchers and IRBs often focus on the possibility of creating alarm [17], we have not observed this in our studies or other studies familiar to us [12,18,19].

Additional ethical concerns have been raised about reporting results when there is uncertainty in the exposure measurements themselves. For example, one-time assessments may not be representative of exposure to some chemicals, and this limitation should be explained.

The ethical framework for community-based participatory research (CBPR) offers an additional perspective that de-emphasizes clinical medicine and emphasizes community impact [20]. CBPR conceptualizes research as a joint effort of researchers, community members, and study participants. It values mutual respect, open communication, shared decision-making, co-ownership of data, and empowerment [21]. This perspective highlights the potential of report-back to inform and empower constructive action to improve public health. In addition, CBPR considers the rights of research communities, not just individuals, particularly regarding the potential for stigma or economic harm [16,17]. A key question in CBPR report-back is whether and how participants and communities want to receive their results. In studies that have asked participants, nearly all do want to know [11,19,22,23].

### Informed consent

With an eye toward autonomy, we think that ethical methods give participants a right to know or not know their exposure results. The decision about whether to receive results can be integrated into informed consent as a logical extension of this practice, which arose after past ethical abuses led to requirements for researchers to inform participants about the research protocol and its risks and benefits. Informed consent provides an early opportunity to set expectations about what participants will and won’t be able to learn from their results and ask about their choice. For example, the Three Generations Study (a study of the daughters and granddaughters of women enrolled when they gave birth in Oakland, CA, in 1959–1967) explains:

These results are not designed for medical use and the information you receive may not suggest any actions you can take to reduce your health risk or exposure to these compounds. However, if you do choose to receive these results we will provide you with as much information as we can and will refer you to available resources to help you understand them (Public Health Institute 2012, unpublished G2 consent form).

Legal issues should be addressed. For example, in rare circumstances, a participant who learns about household contaminants might be obligated to disclose those results prior to a home sale. Requirements may apply to regulated contaminants, such as lead, but few chemicals are regulated.

### Designing report-back

Decisions about the content of personal exposure reports can benefit from input from study participants and communities, and they can draw from a growing number of field-tested models. Literatures on risk communication, data visualization, science and health literacy, numeracy, and broader cognitive and social science fields also inform and improve report-back methods. The Report-back Handbook includes examples of text and graphs, and references to evaluations using focus groups or interviews with participants that give confidence in effectiveness. Examples of evaluation methods are included.

We have found that personal exposure reports should answer these basic questions: What did you find? How much? Where did it come from? Is it safe? What should I do? [16]. To make results meaningful, personal reports should explain what is known about health implications and exposure reduction, including both individual and community-level or national actions. This information can include discussions relevant to a particular study, for example addressing toxicity pathways, potential effects of mixtures, and strengths or gaps in government safety standards.

Reports should include both text and graphs, because different people respond better to different forms of communication. Researchers frequently assume that graphs will not be understood in communities with low numeracy, but, on the contrary, well-designed graphs can draw on hard-wired visual capacities to judge differences and relationships [24]. When we give participants results graphs, we find that even some who think the graphs will be “too hard” begin to read and interpret them, thinking aloud about their meaning.

**Figure 1:**
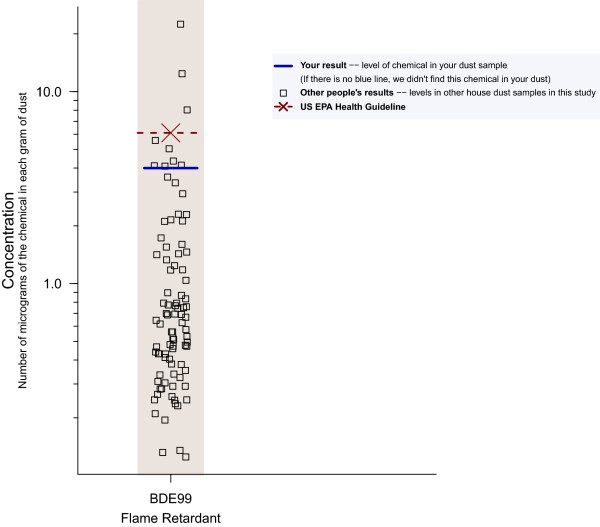
**Strip plot of individual results showing concentration of a flame retardant chemical in house dust.** Strip plots like this one have been used effectively to communicate to participants about their own results in comparison with others in the same study, a health guideline, and other benchmarks, such as NHANES results. This graph format has been evaluated in focus groups and one-on-one usability tests and interviews, including in low-income and recent-immigrant communities. Well-designed graphs have the advantage of drawing on innate visual abilities, relying less on numeracy and literacy.

Comparative data can help participants interpret their results. Comparisons may include government guidelines, if they are available, results for other study participants, and percentile levels from the National Health and Nutrition Examination Survey (NHANES) [25]. Figure 1 illustrates results for an individual in comparison with others in the study and a government health guideline.

To set expectations, researchers can explain that everyday chemicals are commonly detected and that detecting a chemical does not necessarily imply a health risk. For example, the Metals Exposure Study in Homes (MESH) study of exposures near mining sites tells participants, “MESH is designed to measure individual exposures to metals in the environment. It is not attempting to explain the relationship between an exposure and a health outcome” (University of Arizona 2013, unpublished results packet). However, participants can benefit from learning what scientists do know and what potential health concerns led them to select a chemical to measure. For example, the Cape Cod Household Exposure Study fact sheet says:

Other chemicals were chosen for this study because of evidence that they may affect hormones. Some of these chemicals mimic estrogen. They are found in common products, including some pesticides, cleaning products, plastics, furniture, and cosmetics. Exposure to the natural estrogen made in our bodies increases breast cancer risk, so learning about exposure to other chemicals that mimic estrogen may one day help us learn ways to prevent breast cancer [14].

The timing as well as content of report-back is important. Participants want to receive results promptly and to hear from researchers before they hear second hand through the media. On the other hand, researchers are reluctant to release findings prior to revisions that may result from peer review. The C8 Study (a study of perfluorooctanoic acid in residents affected by industrial contamination of drinking water from the Ohio River) resolved these dilemmas by developing a Community-First Model for reporting results after peer-review but before publication. The study developed a precisely timed sequence of communications to study participants, medical providers, news media, and the community [26]. News media reports and community meetings can support and augment individual report-back when these efforts are well-coordinated.

In small studies or studies where researchers are collecting repeat samples, participants can benefit from in-person reports. Studies have also successfully sent results by mail, and we are currently developing interactive online methods to personalize results in large studies, so that people can navigate to results of interest to them and control the level of detail. In any case, a researcher should be available to answer questions or to phone participants with unusual measurements.

In studies that actively invite participants to decide whether to receive their results, nearly all choose to do so. Other studies have required participants to contact the study team to request their report. This method appears to be a barrier to access and may not ensure that all participants who want their results will get them [17,19].

### Experiences from the field

The report-back approaches we describe have been adopted in a variety of settings, including low-income and immigrant communities. We have written about interviews with participants and researchers [12,17,18], and we are analyzing interviews from additional studies. Here we summarize themes from interviews and small-group discussions in multiple settings in order to give researchers considering report-back a sense of what to expect.

#### ***Participants were not excessively worried***

Our first concern was to assess whether participants were unduly worried by their results and the scientific uncertainties associated with them. In the studies we have examined, where researchers prepared reports with care to make them understandable and meaningful, participants have been grateful for their results and worries were kept in perspective. Participants were often surprised to learn that their bodies harbor chemicals from everyday consumer products, pollution, and even chemicals banned years ago, but they quickly began assimilating information and thinking about its meaning and solutions.

#### ***Participants learned about environmental health and some took steps to reduce exposures***

Personal exposure report-back is a powerful tool for increasing understanding of environmental health. In the Household Exposure Study [12], as well as other studies we are analyzing now, people often thought initially that military and industrial facilities were the major source of contaminants and learned from their results that chemical exposures can come from everyday consumer products, such as furniture, cleaners, cosmetics, and pesticides, purchased recently or lingering from years ago. A mother whose daughter was tested as part of the Cohort of Young Girls’ Nutrition, Environment, and Transitions (CYGNET) Study (a San Francisco Bay Area cohort study of puberty in girls) describes her changed awareness:

I really thought I was lily white and pure…but all of a sudden I read, “we detected 19 chemicals in your daughter’s urine,’ and I’m like… I have residues from insecticides and disinfectants and mothballs, and…I realized it’s from fragrances and soaps and detergents and things [14].

#### ***Participants began thinking about possible sources of chemicals in their bodies and homes, and strategies to reduce exposure***

Those who had already tried to be “green” shoppers considered the limits of individual actions for avoiding exposures and began asking questions about the role of government [18].

Many express intentions to change their exposures, and some describe changes they actually made. Follow-up to evaluate the extent to which report-back generates lasting change is an important avenue for future study. Changes could be in individual behavior or at the community level, as illustrated by this participant in the Household Exposure Study who decided to attend a public meeting about emissions from a nearby refinery:

At first I was thinking, ‘God, I wish I didn’t know all this.’ But the more I think about it, the more I understand it, the more I feel like it helps me to…to try to do whatever I can to mitigate or alleviate the toxins that are in my environment…If you don’t know the information, then you have an excuse for not being active. But if you know the information, then you can’t not participate in trying to make change [18].

#### ***Participants felt respected and grateful, and saw their contribution to science in a brighter light***

Another consistent theme from participant interviews is gratitude for researchers’ time, attention, and honesty. Perhaps because the results are complex and uncertain, participants felt respected and more trusting of researchers. After seeing their results, participants understood better their own contribution to knowledge and to future health solutions.

#### ***Researchers found report-back demanding but beneficial***

Researchers found the process intellectually challenging and time-consuming, and were concerned that it required skills beyond their expertise. However, they were glad they had done it. Their anticipatory fears that people would be alarmed were not realized. Researchers conducting cohort studies found the process strengthened relationships and commitment to participation.

### Benefits for research, environmental health literacy, and civil society

While we take a “rights” perspective on why researchers should report individual results, we also see benefits for the researchers themselves and for the standing of science in society. Researchers benefit because the report-back process strengthens relationships with participants and can support recruitment, retention, and trust in science. Report-back also can influence the researchers’ own thinking and public understanding of the science as well. While researchers generally focus on statistical measures of central tendency and relationships across distributions, report-back draws attention to outliers, which can lead to different kinds of discovery. When we prepared the individual reports for the Household Exposure Study, for example, we noted two individuals whose polychlorinated biphenyl (PCB) house dust levels were exceptionally high. Knowing that we would be talking to those participants motivated us to re-test their dust, collect blood samples, inspect the homes, and interview the residents in search of an explanation. We discovered that a floor finish was a likely source in these homes and a widespread, previously neglected source in older homes in general [27]. Extreme exposures, even in a mid-sized study, can represent a substantial number of people if those outliers represent an exposure scenario that is generalizable to the US population, so communicating with participants in these contexts can inform targeted public health interventions.

In addition to generating novel discoveries, report-back stimulates trans-disciplinary and integrative thinking that can help researchers develop the translational aspects of their findings. Writing the interpretive text for reports requires researchers to synthesize what is known about biological and human health effects of chemicals, and chemical sources, fate in the environment, and exposure pathways.

In the public sphere, as individuals learn their own exposure results and share them with family, friends, doctors, and public leaders, we envision the potential to raise the civic discourse about environmental public health. In a democratic society, data that make the invisible residues of consumer products and pollution known to the affected individuals and communities can empower them to make decisions about research funding, public policies, and their own behavior.

### Future needs

Experiences with reporting individual results have been positive, but researchers – both those who have reported results and those who have not – identify important needs. Concerns about resources and expertise for report-back are at the top of the list. To make report-back practical, researchers need access to models that can be adapted in diverse study settings. Our Report-back Handbook (see Additional file 3) is a starting point for creating a library of methods that researchers and community partners can adapt to their own studies. Sharing, building upon, and continually improving these resources will make report-back easier and encourage broader use of field-tested methods.

Improved IRB training in human research ethics for CBPR and, specifically, report-back ethics could also reduce delays and constraints that can unintentionally undermine participants’ trust in research and the effectiveness of report-back [28]. For example, IRBs need strategies that guide report-back without approval of every iteration, which can interfere with the natural back-and-forth between researchers and participants as they design report-back that is responsive to a particular community and address questions that arise. Researchers also need training in report-back methods and evaluation techniques, for example, through webinars and consultations with experienced practitioners.

Some important ethical questions remain unanswered for reporting environmental results, as for genetic and other studies. Even if individual report-back becomes the norm, are there particular situations in which it is not appropriate? What responsibilities do researchers have to help participants reduce high exposures, particularly in situations when the participants may not have the personal resources themselves? This question has a parallel in medical research, but health insurance and clinical care systems exist to respond to medical findings, while environmental study participants lack a parallel avenue for remediation.

### Key recommendations for reporting personal exposure results in community-based research

For research teams that are seeking guidance about report-back, we recommend these key considerations.

1. Plan for report-back when you plan your study. Budget time and money to get it done.

2. Involve study participants or others who can represent them throughout the process, so the plan is tailored for participants and communities.

3. Expect senior researchers to play a role in interpreting individual results, adding their experienced judgment of what results mean.

4. Educate the IRB in advance about CBPR values and advocate for the IRB to include at least one board member who has CBPR expertise. This perspective is relevant to individual report-back even if your study doesn’t use CBPR methods.

5. Ask participants whether they want their results when you get informed consent to participate in the study. Set expectations for what the study will and won’t be able to tell people about their exposures and health.

6. When health implications are uncertain, explain what is and is not known, including why you are studying the target chemicals.

7. Include both text and graphs in personal reports. Different people prefer different approaches. Draw attention to what’s important.

8. When there isn’t a clear health guideline for what exposure level is “safe,” use comparisons, such as the National Exposure Report or other study participants, to help put findings in perspective. But sometimes it’s important to communicate that “the same as everybody else” could represent community-wide risks, and “high” compared with others might still be safe.

9. Be sure to include information about how people can reduce exposures when this is possible. If exposure reduction strategies require policy change, say so. And, if you can, connect participants to opportunities to get involved.

10. Report aggregate-level findings to participants and their communities to put individual results in context and generate dialogue about the study implications. Also, this allows you to reach far more people than just the participants.

11. Make a plan for how to respond to findings of extremely high exposures. In some instances, re-analysis to confirm laboratory results may be a sensible first step.

12. Consider how to involve medical practitioners or other local leaders as advisers or resources. Be sure they have the information they need to provide accurate and useful guidance.

13. Pretest report-back materials on a few people who are similar to study participants. Ask them to speak for themselves rather than speculating about how someone else would respond.

14. Don’t forget to reflect on what you learned about your data by focusing on individual results and what you learned from your report-back experiences. Share what you learned.

## Conclusions

Researchers and IRBs have often speculated that reporting to people on their own chemical exposures might be harmful, because results could generate excessive worry when the health effects and remedies are unclear. However, study participants generally want their results, and studies that have reported individual results along with comparative benchmarks and interpretive context find that participants benefited by learning a great deal about environmental health. They were able to understand results without undue alarm and began to consider possible exposure reduction strategies. In addition, the human research ethics principles of beneficence and autonomy and the additional perspectives of CBPR favor a “research right-to-know.” Researchers benefit from strengthened relationships with participants and new opportunities for scientific insight. Taken together, ethical principles and empirical observations suggest that individual report-back should become standard practice in most studies. Studies that have implemented individual report-back provide guidance for researchers and IRBs to adopt report-back practices that respond to the particular community context of research and help individuals understand the meaning of their results.

## Abbreviations

CBPR: Community-based participatory research; COPHES: Consortium to Perform Human biomonitoring on a European Scale; DEMOCOPHES: Demonstration of a study to Coordinate and Perform Human biomonitoring on a European Scale; ESBIO: Expert team to Support BIOmonitoring in Europe; IRB: Institutional review board; NHANES: National Health and Nutrition Examination Survey; PBDE: Polybrominated diphenyl ether; PCB: Polychlorinated biphenyl.

## Competing interests

The authors declare they have no competing interests.

## Authors’ contributions

Concepts were developed from the longstanding research collaboration of JGB, RAR, PB, and RMF. JGB drafted the manuscript. SD compiled report-back models from other studies and drafted the report-back handbook. JB, PB, RMF, and SD conducted interviews and consultations, and led the stakeholder workshop. SP participated in the workshop and contributed input from the perspective of study participants. All authors contributed to drafting and revising of the manuscript and have read and approved it.

## Supplementary Material

Additional file 1 Workshop on the Ethics of Reporting Personal Environmental Exposures: Agenda. Click here for file

Additional file 2 **Workshop on the Ethics of Reporting Personal Environmental Exposures: Participants and Affiliations.** The authors convened a workshop of researchers, IRB representatives, study participants, government agency representatives, ethicists, lawyers, and community leaders to discuss ethics, best practices, and past experiences with reporting individual exposure results when the health effects are uncertain. This discussion was important groundwork for this commentary. Click here for file

Additional file 3 **When Pollution is Personal: Best Practices for Reporting Results to Participants in Biomonitoring and Personal Exposure Studies.** This Report-Back Handbook provides guidance on decision-making and planning in studies that report personal environmental exposure results. It includes examples of informed consent language, narrative results and graphs, and references to evaluations. It was reviewed by researchers and community leaders who participated in the workshop as well as others. Click here for file
