# Supplementary material for: Reporting individual results for biomonitoring and environmental exposures: lessons learned from environmental communication case studies
Source: Environ Health. 2014 May 26;13:40. doi: 10.1186/1476-069X-13-40 (PMC4098947; doi:10.1186/1476-069X-13-40)
Supplement: Additional file 1 — Workshop on the Ethics of Reporting Personal Environmental Exposures: Agenda. [file 1476-069X-13-40-S1.pdf]

Silent Spring Institute
Brown University
University of California, Berkeley
Harvard Law School

Workshop on the Ethics of Reporting Personal Environmental Exposures
Tuesday, September 21, 2010
Harvard Law School, Cambridge, MA (Hauser 104)

AGENDA

**8:00 am**	**Registration and Breakfast**
**8:30 am**	**Welcome and Framing**<br>Wendy Jacobs, Harvard Law School<br>Julia Brody, Silent Spring Institute
**9:00 am**	**Panel 1: Perspectives of Participants**<br>Moderator: Rachel Morello-Frosch, University of California, Berkeley<br>Panelists:<br>o Jennifer Hill-Kelley, Oneida Tribe of Wisconsin<br>o Sharyle Patton, Commonweal
**9:45 am**	**Panel 2: Experiences of Researchers**<br>Moderator: Julia Brody, Silent Spring Institute<br>Panelists:<br>o Susan Pinney, University of Cincinatti<br>o Ted Emmett, University of Pennsylvania School of Medicine<br>o Larry Kushi, Kaiser Permanente<br>o Rachel Morello-Frosch, University of California, Berkeley
**11:00 am**	**Break**
**11:15 am**	**Panel 3: Legal Dimensions**<br>Moderator: Rachel Morello-Frosch, University of California, Berkeley<br>Panelists:<br>o Shaun Goho, Harvard Law School<br>o Laura Hall, University of California, Berkeley, School of Law
**12:30 pm**	**Lunch**
**1:30 pm**	**Panel 4: Ethical Frameworks & Parallels to Biomedical Report-back**<br>Moderator: Phil Brown, Brown University<br>Panelists:<br>o Katsi Cook, Running Strong for American Indian Youth<br>o Tom Delbanco, Harvard Medical School<br>o David Resnik, National Institute of Environmental Health Sciences
**2:45 pm**	**Synthesis and Recommendations**<br>Julia Brody, Silent Spring Institute<br>Phil Brown, Brown University
**3:30 pm**	**Adjourn**
**4:00 pm**	**Public Forum: Margaret Kripke, President's Cancer Panel,**<br>***Reducing Environmental Cancer Risk: What We Can Do Now***

This workshop is part of the Personal Exposure Report-back Ethics (PERE) Study funded by NIEHS.
