# Supplementary material for: Reporting individual results for biomonitoring and environmental exposures: lessons learned from environmental communication case studies
Source: Environ Health. 2014 May 26;13:40. doi: 10.1186/1476-069X-13-40 (PMC4098947; doi:10.1186/1476-069X-13-40)
Supplement: Additional file 2 — Workshop on the Ethics of Reporting Personal Environmental Exposures: Participants and Affiliations. The authors convened a workshop of researchers, IRB representatives, study participants, government agency representatives, ethicists, lawyers, and community leaders to discuss ethics, best practices, and past experiences with reporting individual exposure results when the health effects are uncertain. This discussion was important groundwork for this commentary. [file 1476-069X-13-40-S2.pdf]

Silent Spring Institute
Brown University
University of California, Berkeley
Harvard Law School

**Workshop on the Ethics of Reporting Personal Environmental Exposures**

Tuesday, September 21, 2010
Harvard Law School, Cambridge, MA (Hauser 104)

## PARTICIPANTS AND AFFILIATIONS

Beth Anderson,National Institute of Environmental Health Sciences

Kathleen Attfield, Harvard School of Public Health, Environmental, Epidemiology, and Risk Program; Silent Spring Institute

Cynthia Barakatt, Silent Spring Institute; Boston University

Janice Barlow, Zero Breast Cancer

Julia Brody, Silent Spring Institute

Phil Brown, Brown University, Department of Sociology

Jane Burns, Harvard School of Public Health

Jennifer Burt, President's Cancer Panel

Suzanne Condon, Massachusett Department of Public Health

Katsi Cook, First Environment Collaborative at Running Strong for American Indian Youth

Alissa Cordner, Brown University, Department of Sociology

Kathy Curtis, Clean New York

Tom Delbanco, Harvard Medical School

Sarah Dunagan, Silent Spring Institute

Ted Emmett, University of Pennsylvania School of Medicine

Sarah Fort, Harvard Law School

Shaun Goho, Harvard Law School, Emmett Environmental Law and Policy Clinic

Laura Hall, University of California, Berkeley, School of Law

Jennifer Hill-Kelley, Oneida Tribe of Wisconsin

Wendy Jacobs, Harvard Law School, Emmett Environmental Law and Policy Clinic

Christine James, The John Merck Fund

Tania Jenkins, Brown University, Department of Sociology

Allan Just, Columbia University, Mailman School of Public Health

Margaret Kripke, President’s Cancer Panel; University of Texas M.D. Anderson Cancer Center

Larry Kushi, Kaiser Permanente Northern California Division of Research

Diana Lee, California Department of Public Health, Environmental Health Investigations Branch

Cindy Luppi, Clean Water Action

Marty McCoy, Harvard Law School

Rachel Morello-Frosch, University of California, Berkeley, Department of Environmental Science, Policy and Management & School of Public Health

Cheryl Osimo, Silent Spring Institute; Massachusetts Breast Cancer Coalition

Bindu Panikkar, Brown University, Department of Sociology

Lisa Paradis, President's Cancer Panel

Sharyle Patton, Commonweal

Susan Pinney, University of Cincinnati College of Medicine

Dianne Quigley, Brown University, Center for Environmental Studies

Les Reinlib, National Institute of Environmental Health Sciences

David Resnik, National Institute of Environmental Health Sciences

Ruthann Rudel, Silent Spring Institute

Madeleine Scammell, Boston University School of Public Health

Deborah Shields, Massachusetts Breast Cancer Coalition

Daniel Steinberg, Allergy & Asthma Center of Massachusetts

Jessica Tovar, Communities for a Better Environment

MarshaVanderford, Centers for Disease Control and Prevention, Emergency Risk Communication Branch

Julia Varshavsky, University of California, Berkeley, School of Public Health
