# Supplementary material for: Reporting individual results for biomonitoring and environmental exposures: lessons learned from environmental communication case studies
Source: Environ Health. 2014 May 26;13:40. doi: 10.1186/1476-069X-13-40 (PMC4098947; doi:10.1186/1476-069X-13-40)
Supplement: Additional file 3 — When Pollution is Personal: Best Practices for Reporting Results to Participants in Biomonitoring and Personal Exposure Studies. This Report-Back Handbook provides guidance on decision-making and planning in studies that report personal environmental exposure results. It includes examples of informed consent language, narrative results and graphs, and references to evaluations. It was reviewed by researchers and community leaders who participated in the workshop as well as others. [file 1476-069X-13-40-S3.pdf]

WHEN POLLUTION IS PERSONAL

HANDBOOK FOR REPORTING RESULTS TO PARTICIPANTS
IN BIOMONITORING AND PERSONAL EXPOSURE STUDIES

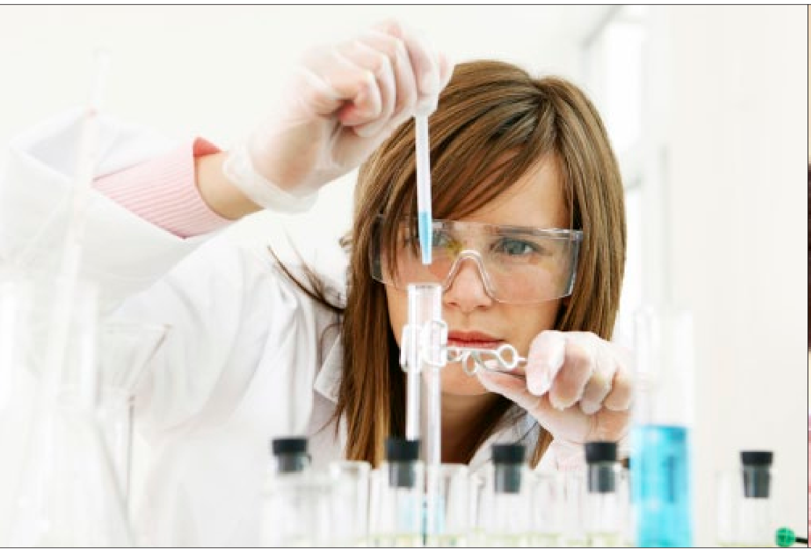

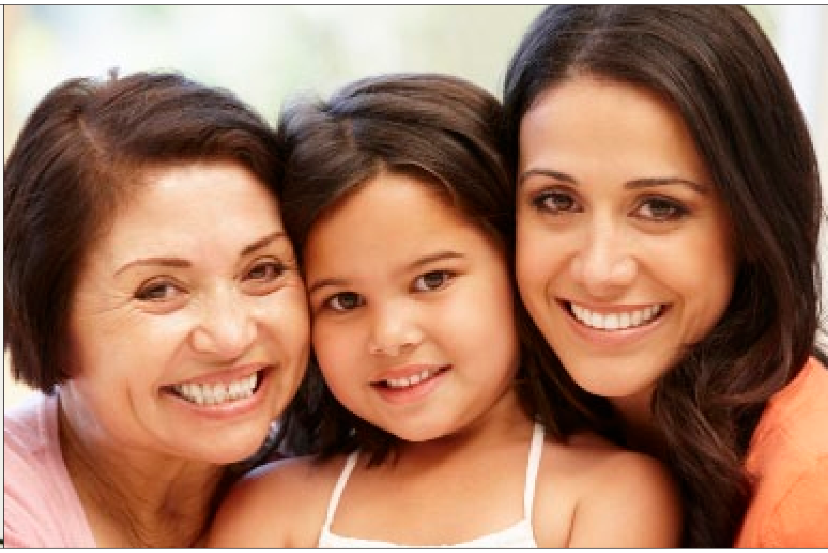

The Personal Exposure Report-back Ethics (PERE) Study
This publication reports on work supported by NIEHS grants R01ES017514 and R25ES013258.

SILENT SPRING INSTITUTE

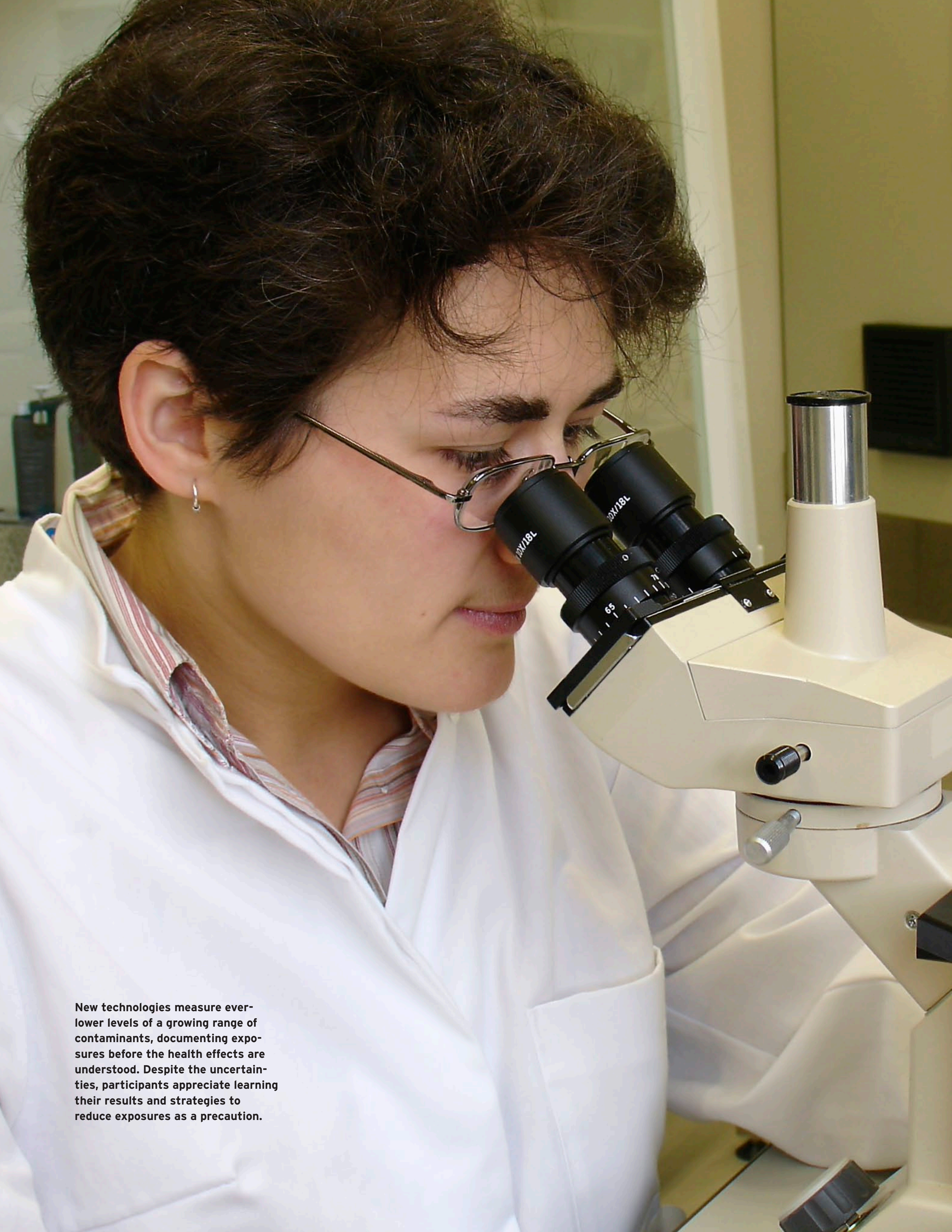

New technologies measure ever-lower levels of a growing range of contaminants, documenting exposures before the health effects are understood. Despite the uncertainties, participants appreciate learning their results and strategies to reduce exposures as a precaution.

WHEN POLLUTION IS PERSONAL

HANDBOOK FOR REPORTING RESULTS TO PARTICIPANTS IN BIOMONITORING AND PERSONAL EXPOSURE STUDIES

AUTHOR LIST:
Sarah C. Dunagan[a], Julia G. Brody[a], Rachel Morello-Frosch[b], Phil Brown[c], Shaun Goho[d], Jessica Tovar[e], Sharyle Patton[f], Rachel Danford[a]

[a]Silent Spring Institute, Newton, MA,

[b]University of California, Berkeley, CA

[c]Northeastern University, Boston, MA

[d]Harvard Law School Emmett Environmental Law and Policy Clinic, Cambridge, MA

[e]Communities for a Better Environment, Oakland, CA

[f]Commonweal Biomonitoring Center, Bolinas, CA

Please address correspondence to Julia Brody, info@silentspring.org.

The Personal Exposure Report-back Ethics (PERE) Study
This publication reports on work supported by NIEHS grants R01ES017514 and R25ES013258.

Copyright © 2013 by Silent Spring Institute
Silent Spring Institute grants permission to copy, distribute, and display the information in this publication. Please give credit to Silent Spring Institute and the original author(s) of the material that you use.

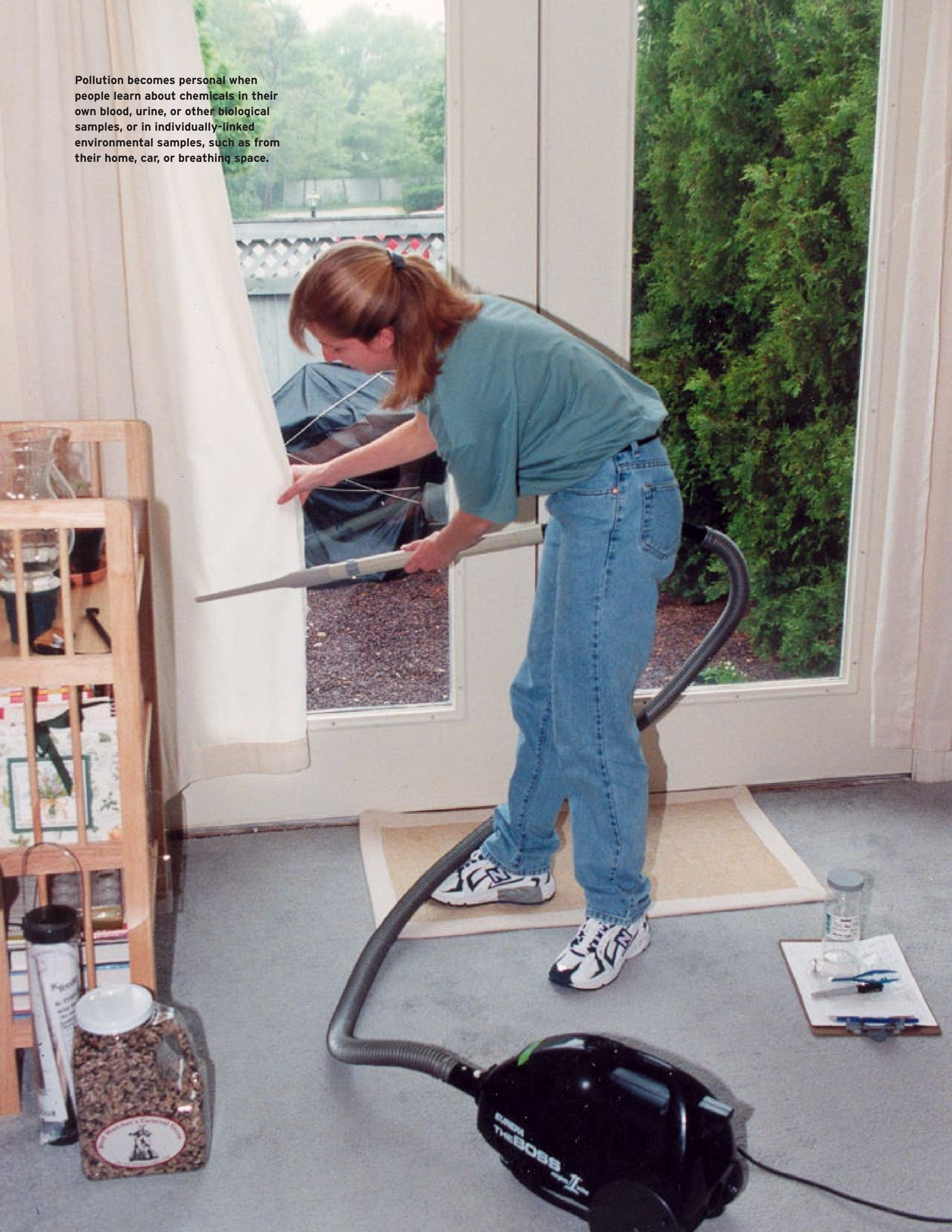

Pollution becomes personal when people learn about chemicals in their own blood, urine, or other biological samples, or in individually-linked environmental samples, such as from their home, car, or breathing space.

TABLE OF CONTENTS

5 LIST OF CHARTS AND FIGURES

7 TWELVE TIPS FOR REPORTING PERSONAL EXPOSURE RESULTS

9 INTRODUCTION

13 CHAPTER 1: GETTING STARTED

- 13 Planning ahead
- 13 Deciding whether to report
  - 13 Ethical principles
  - 14 CBPR approaches consider community and participant views, support health-protective action, and build trust
- 15 Anticipating the costs
- 17 Roles and responsibilities
- 17 Informed consent
- 18 Legal issues
- 18 Communicating results for children
- 18 Navigating the IRB process

21 CHAPTER 2: METHODS FOR REPORTING RESULTS

- 21 More than "one size fits all"
- 21 Considering principles of risk communication
- 22 Deciding what to report
- 23 Designing report-back materials
  - 23 Combining graphs and text
  - 26 Graphs and tables
  - 26 Examples of individual results
  - 27 Study-wide results
  - 38 Put results in context with comparisons
  - 38 Highlight key messages with text
  - 39 Address the need for information about actions to reduce exposure
  - 39 Individual actions
  - 39 Community-level and policy action
  - 40 Getting comfortable with uncertainty
  - 40 Consider varying levels of literacy and numeracy
  - 40 Consider community context
  - 41 Pilot test materials
- 41 Distributing results
  - 43 Active versus passive reporting
  - 43 Timing
  - 44 Community first report-back
  - 44 Develop a response plan

47 CHAPTER 3: EVALUATING REPORT-BACK

- 47 Why evaluate the report-back process?
- 47 Planning ahead
- 47 Interviewing participants about their experiences
  - 47 Developing an interview protocol
  - 48 Getting informed consent, again
  - 49 Selecting interviewers
  - 49 Conducting interviews
  - 49 Case Study: Household Exposure Study
  - 50 Case Study: La Familia Study
  - 50 Case Study: Greater Boston PBDE Breastmilk Biomonitoring Study
  - 50 Case Study: The Growing Up Female Study
- 50 Evaluating community meetings with surveys

53 CONCLUSION

55 REFERENCES

59 RESOURCES

61 APPENDIX

- A. California Household Exposure Study sample report-back packet
- B. Chemicals in Our Bodies Study sample report-back packet
- C. Green cleaning fact sheet
- D. Take action fact sheet
- E. California Household Exposure Study interview
- F. Community meeting survey: post-forum questionnaire

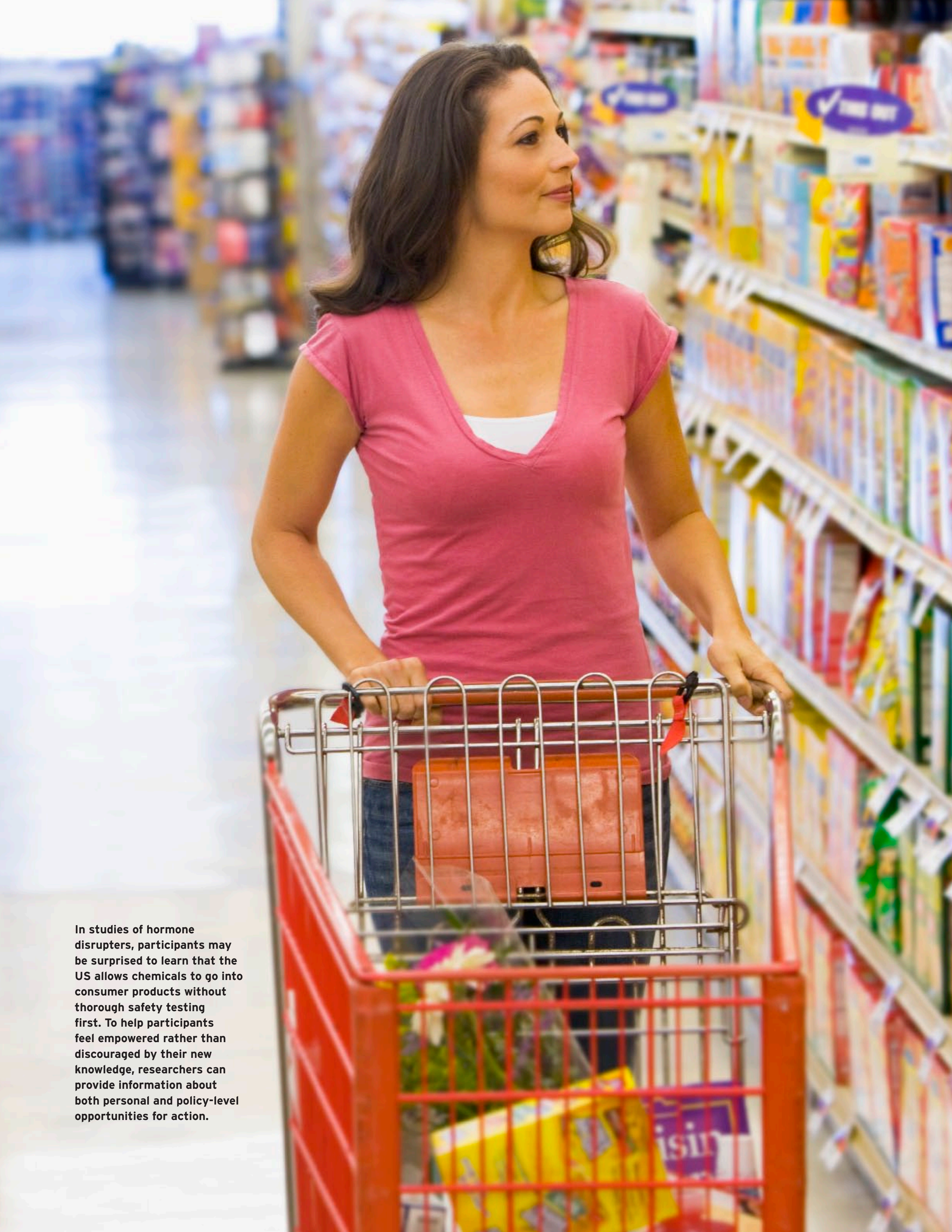

In studies of hormone disrupters, participants may be surprised to learn that the US allows chemicals to go into consumer products without thorough safety testing first. To help participants feel empowered rather than discouraged by their new knowledge, researchers can provide information about both personal and policy-level opportunities for action.

LIST OF CHARTS AND FIGURES

16 **Table 1.** Selected personal exposure and biomonitoring studies that have reported individual results to participants.

22 **Table 2.** Report-back information that helps answer typical participant questions.

24 **Figure 1.** Examples of guides that can be used as a roadmap for interpreting graphical presentations of individual exposure data, data from other participants, national averages, and benchmarks.
a) A guide from the Northern California Household Exposure Study.
b) A guide from the Chemicals in Our Bodies Study.

28 **Figure 2.** Strip plot used to communicate levels of phthalates, flame retardants, and PCBs measured in dust in a home in the Household Exposure Study (Rudel 2010).

29 **Figure 3.** A strip plot comparing individual levels of brominated flame retardants (PBDEs) to levels in other participants of the same study and to the national average (Brown-Williams 2009b).

30 **Figure 4.** Bar chart reporting individual levels of bisphenol A in participants' blood and urine relative to the national median for bisphenol A (Commonweal Biomonitoring Resource Center and the Body Burden Work Group 2007).

31 **Figure 5.** Shaded grid showing the number and types of pesticides found in study participants' homes. This graph included a key on the reverse side indicating which agricultural pesticides were represented by each heading (Quandt 2004).

32 **Figure 6.** Reports in the Cape Cod Drinking Water Study used colors to show contaminant levels above natural background levels or above a guidance level.

33 **Figure 7.** Box plots comparing levels of fine particulate matter ($PM_{2.5}$) in two study communities in the Northern California Household Exposure Study. These aggregate results can also be compared to benchmarks to provide additional context for interpreting results.

34 **Figure 8**. Strip plots comparing levels of fine particulate matter ($PM_{2.5}$) in two study communities in the Northern California Household Exposure Study.

35 **Figure 9.** A cumulative bar graph comparing the number of compounds detected in outdoor air in two study communities in the Northern California Household Exposure Study.

36 **Figure 10.** Strip plot reporting individual blood serum levels of PFOA relative to other study participants and the national average in the Growing up Female Study (Hernick 2007).

37 **Figure 11.** Graph reporting average blood serum levels of PFOA for two study communities relative to workers with high exposure, the national average, and a sample from the San Francisco Bay area (Growing up Female Study) (Hernick 2007).

41 **Figure 12.** Recommendation for further research or action should be calibrated to reflect the level of certainty in knowledge about health effects and exposure reduction methods (Brody 2007).

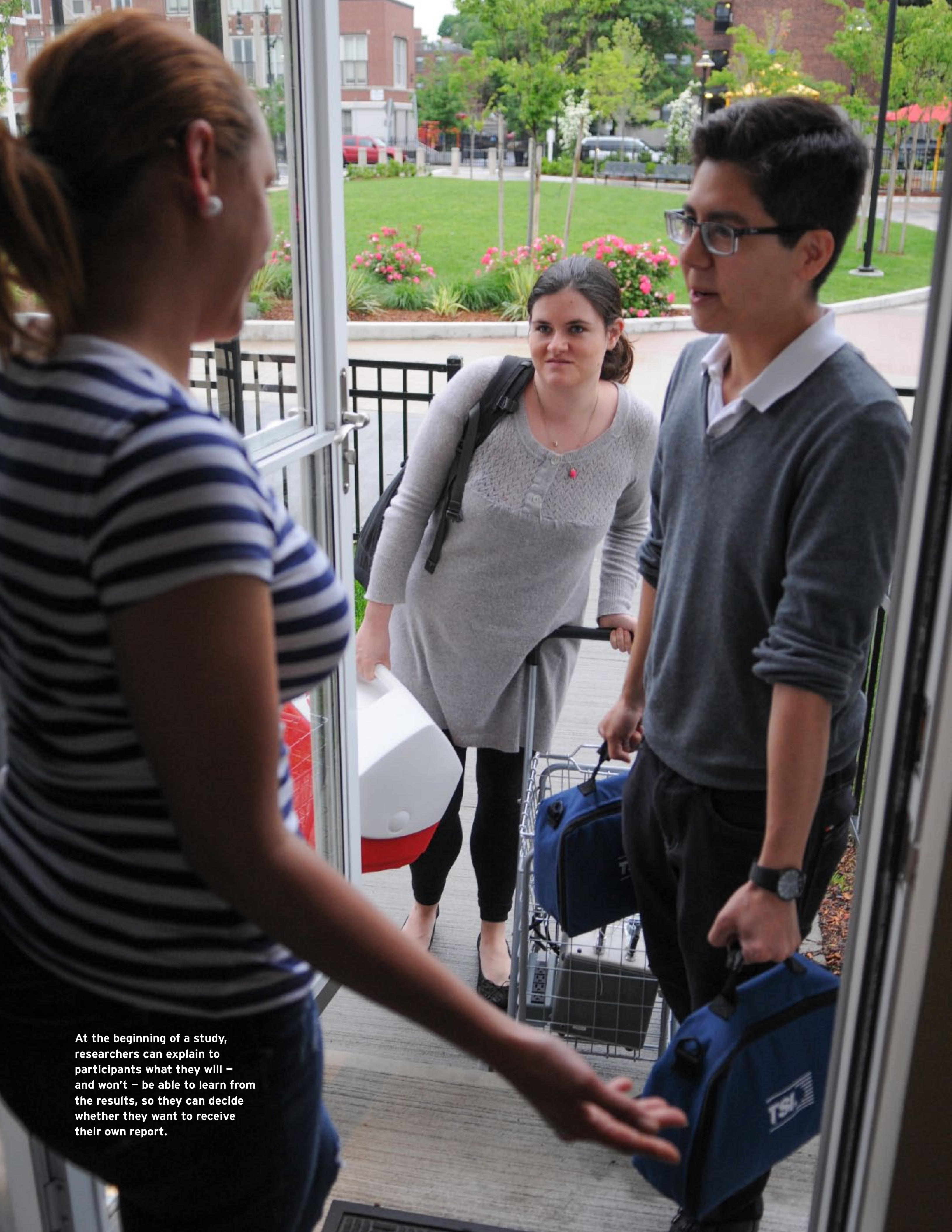

At the beginning of a study, researchers can explain to participants what they will — and won't — be able to learn from the results, so they can decide whether they want to receive their own report.

TWELVE TIPS FOR REPORTING PERSONAL EXPOSURE RESULTS

Here's our top advice for reporting personal exposure results in a CBPR context.

1. Plan for report-back when you plan your study. Budget time and money to get it done.
2. Involve study participants or others who can represent them throughout the process, so the plan is tailored for participants and communities.
3. Expect senior researchers to play a role in interpreting individual results, adding their experienced judgment of what results mean.
4. Educate the IRB in advance about CBPR values and advocate for the IRB to include at least one board member who has CBPR expertise. This perspective is relevant to individual report-back even if your study doesn't use CBPR methods.
5. Ask participants whether they want their results when you get informed consent to participate in the study. Set expectations for what the study will and won't be able to tell people about their exposures and health.
6. When health implications are uncertain, explain what is and is not known, including why you are studying the target chemicals.
7. Include both text and graphs in personal reports. Different people prefer different approaches. Draw attention to what's important.
8. When there isn't a clear health guideline for what exposure level is "safe," use comparisons, such as the National Exposure Report or other study participants, to help put findings in perspective. But sometimes it's important to communicate that "the same as everybody else" could represent community-wide risks, and "high" compared with others might still be safe.
9. Be sure to include information about how people can reduce exposures when this is possible. If exposure reduction strategies require policy change, say so. And, if you can, connect participants to opportunities to get involved.
10. Report aggregate-level findings to participants and their communities to put individual results in context and generate dialogue about the study implications. Also, this allows you to reach far more people than just the participants.
11. Pretest report-back materials on a few people who are similar to study participants. Ask them to speak for themselves rather than speculating about how someone else would respond.
12. Don't forget to reflect on what you learned about your data by focusing on individual results and what you learned from your report-back experiences. Share what you learned with others.

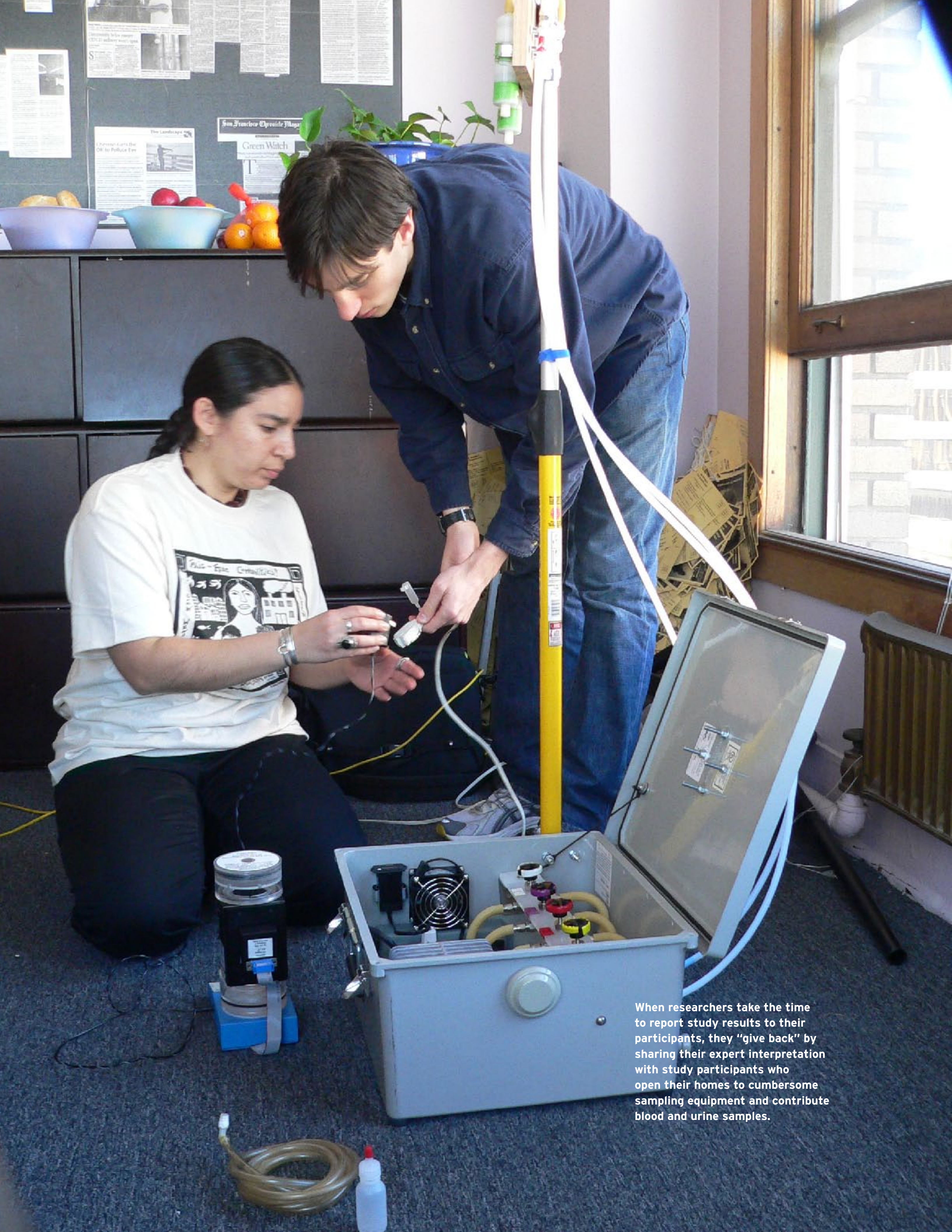

When researchers take the time to report study results to their participants, they "give back" by sharing their expert interpretation with study participants who open their homes to cumbersome sampling equipment and contribute blood and urine samples.

INTRODUCTION

IN 2003, CAPE COD COMMUNITY MEMBERS GENEROUSLY OPENED THEIR DOORS TO SILENT SPRING INSTITUTE RESEARCHERS TO CONDUCT A GROUNDBREAKING STUDY OF HOUSEHOLD EXPOSURES TO ENVIRONMENTAL POLLUTANTS. STUDY PARTICIPANTS WELCOMED RESEARCHERS INTO THE INTIMATE SPACE OF THEIR HOMES, OFFERING TEA AND SHARING INFORMATION ABOUT THEIR PERSONAL HISTORY AND PRODUCT USE, WHILE RESEARCHERS COLLECTED AIR AND DUST SAMPLES FROM THEIR LIVING ROOMS WITH NOISY MACHINES AND RETRIEVED JARS OF URINE SAMPLES.

Shortly thereafter, study participants began calling to ask what the researchers had found and if it was safe. Their questions raised difficult issues about whether and how to report individual results when the health effects were so uncertain. For some chemicals, these were the first measurements ever reported in homes, so we didn't yet know what levels were typical in the US. Despite these uncertainties, the research team, in consultation with community members, decided that study participants had a right to decide whether or not they wanted to receive their own results.

Since the Silent Spring Institute studies began, personal exposure research, including biomonitoring, has become a cornerstone for environmental health research and advocacy.

> Biomonitoring involves the measurement of environmental chemicals in human blood, urine, breast milk, saliva, breath or other tissue (Pausentbach 2006). Personal exposure assessment also measures chemicals in personal spaces such as homes, automobiles, and the breathing space near a person's face. Biomonitoring and personal exposure studies are being conducted by researchers, advocates, public health officials, and communities. Nationally, the US Centers for Disease Control and Prevention (CDC) is systematically tracking exposures to hundreds of chemicals in blood and urine collected from a representative sample of the US population. In 2006, California became the first state to mandate its own statewide biomonitoring program, and state law requires the program to provide individual results to study participants who want them (Perata 2006).

"I MEAN I'M SURPRISED THAT THEY CAN FIND THAT MANY THINGS BY LOOKING AT YOUR DUST AND LOOKING AT YOUR AIR. I MEAN THAT'S AMAZING TO ME THAT THEY CAN ACTUALLY FIND CHEMICALS IN YOUR AIR IN ANY AMOUNT WHATSOEVER."

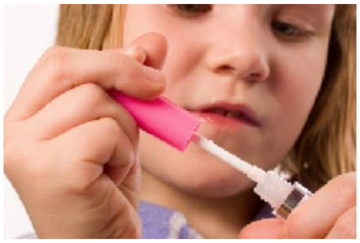

When study participants learn that their personal exposures can be linked to everyday products like make-up, they have an opportunity to change their actions to reduce exposures.

Yet early exposure measurements often outpace our understanding of the health implications and strategies for reducing exposures. The 2006 National Academy of Sciences (NAS) report *Human Biomonitoring for Environmental Chemicals* noted that as new technologies measure lower concentrations of larger numbers of chemicals, new challenges arise about how to interpret, report, and act on results that only partially illuminate the links between environmental chemicals and health (National Academy of Sciences 2006).

Decisions about whether and how to report study results to participants must weigh two dimensions. On one dimension, report-back may motivate behavior change and protective public health policies, increase trust in and understanding of research, and respect participants' autonomy. On the other dimension, there is potential for harm, for example, from worry, stigma, or ineffective action to reduce exposures.

A growing number of study teams are deciding that participants have a right to know their individual results if they want them, but there is little information about how to report results ethically and effectively. The NAS report recommended sharing information about multiple approaches in order to develop best practices. There's been a catch-22, though, because some IRBs have been reluctant to allow development of individual report-back alternatives. Perhaps these decision-makers are not aware of successful examples of individual-level report-back.

This handbook aims to address this information gap about report-back practices by sharing the lessons learned by our team, beginning from the Household Exposure Study (HES), an ongoing study of exposures to endocrine disrupting compounds (EDCs) in homes. The HES team is an interdisciplinary collaboration led by Silent Spring Institute with partners at Communities for a Better Environment, Brown University, Northeastern University, the University of California, Berkeley, Commonweal, and the Harvard Law School Emmett Environmental Law and Policy Clinic. This handbook draws on our experience reporting community and individual results, and interviewing participants about their experiences after they received their reports. We also incorporate our observations and interviews in the studies that are part of our National Institute of Environmental Health Sciences (NIEHS)-funded Personal Exposure Report-back Ethics (PERE) Study, and we draw on our day-long workshop of 40 researchers, study participants, IRB representatives, and state and federal agency officials. Our goal is to help guide other study teams, including researchers, advocates, IRB members, and public health officials through decisions on whether and how to report study findings to participants.

As more teams begin reporting individual exposure results, we hope to share additional experiences and perspectives. Please send comments, questions, and examples of report-back materials to brody@silentspring.org.

“I’M SURROUNDED BY CHEMICALS. MANY OF THEM THAT, YOU KNOW, IT JUST SHOWS YOU HOW MANY CHEMICALS YOU ARE AROUND. I MEAN, THIS IS ONLY A PARTIAL LIST [POINTING TO STUDY RESULTS]…AND I THINK THAT SOMETHING HAPPENS ON, YOU KNOW, ON A CELLULAR LEVEL BETWEEN THE BODY AND THE ENVIRONMENT, AND THINGS THAT WE SEE IN OUR ENVIRONMENT ARE NOT AS DANGEROUS AS THE THINGS WE DON’T SEE. WE DON’T SEE ALL THE STUFF…THESE ARE OUT OF SIGHT OUT OF MIND THINGS. AND WE’VE CHANGED OUR ENVIRONMENT SO MUCH SINCE WE’VE LEARNED HOW TO MAKE THINGS AND WE DON’T KNOW HOW ALL THIS AFFECTS THE BODY, BUT WE DO KNOW IT’S AFFECTING, YOU KNOW, THERE ARE SO MANY THINGS GOING ON NOW, YOU KNOW, HEALTH-WISE. “

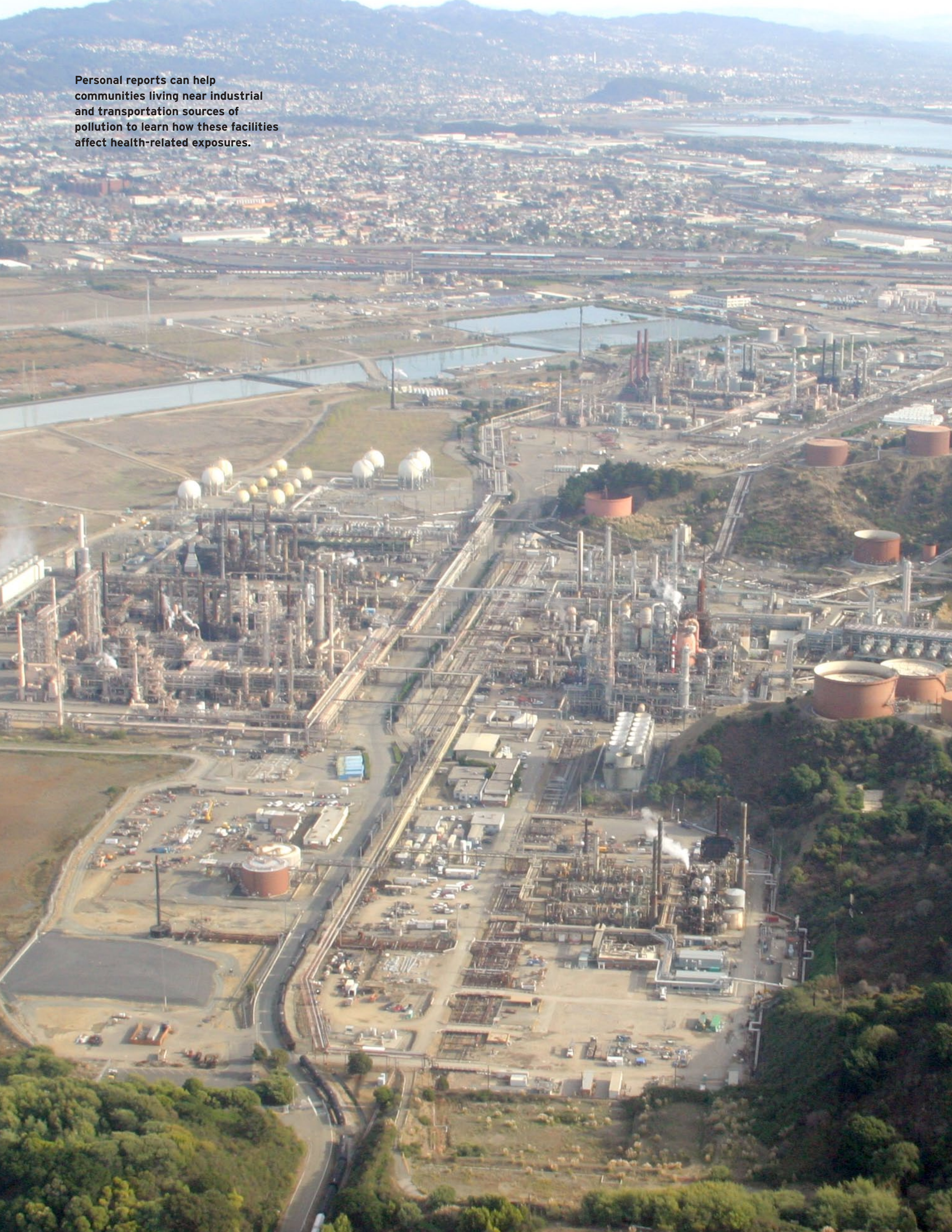

Personal reports can help communities living near industrial and transportation sources of pollution to learn how these facilities affect health-related exposures.

CHAPTER 1: GETTING STARTED

PLANNING AHEAD

Effective report-back begins with planning and communication. One of the first steps is to bring together researchers, community leaders, participants, and other stakeholders to identify communication needs and goals. Getting started early in the research process can help teams ensure that they allocate time and resources and anticipate roadblocks. The National Institute of Environmental Health Sciences (NIEHS) is even beginning to encourage researchers to address these issues in proposed data sharing plans.

This chapter helps teams get started, including deciding whether to report individual results, resource requirements, and team member responsibilities. It includes a discussion of incorporating the report-back plan into the informed consent and tips for working with Institutional Review Boards (IRBs).

DECIDING WHETHER TO REPORT

Should researchers report individual results, including in studies testing for chemicals for which sources of exposure, health effects, and exposure reduction strategies are uncertain? The ethical principles of human subjects research (box) are a helpful jumping off point for answering that question.

Ethical principles

The ethical principles of **autonomy** and **justice** favor reporting individual results. The principle of autonomy directs researchers to provide individuals with the opportunity to decide freely if they wish to become study participants. Extending that principle to communicating results, it follows that study participants have a right to know or not know their individual study results as a basis for self-determination in taking action, for example by making personal changes to reduce exposures or by supporting protective public health policies.

Beneficence guides researchers to consider benefits, such as the potential for results reporting to inform and motivate individuals and communities to take actions to reduce exposures, protect their health, and participate more fully in public health research and policy. **Nonmalfeasance** guides researchers to avoid harm, such as the potential for report-back to cause fear, worry, or stigma; legal and economic com-

ETHICAL PRINCIPLES IN HUMAN SUBJECTS RESEARCH

The guidelines for protecting human subjects in research rest on ethical principles identified in the Common Rule:

- *Autonomy*, which includes respecting participants as individuals capable of self-determination
- *Beneficience* and *nonmalfeasance*, which together encompass the researcher's responsibility to maximize good and minimize harm, and
- *Justice*, which refers to the distribution of benefits and harms (US Department of Health and Human Services 1991; US DHHS 1991).

"HAVING THE COMMUNITY MEETINGS, ESPECIALLY OUT HERE IN ATCHISON VILLAGE, IT REALLY SHOWS THAT THEY DO CARE ABOUT THE AIR QUALITY HERE AND THE PEOPLE HERE. AND THE INFORMATION... HAVING THE SCIENTISTS COME OVER AND TALK AND EXPLAIN THINGS—IT'S VERY ENLIGHTENING."

plications, such as effects on health insurance or property values; and the possible unintended promotion of unnecessary or counter-productive interventions.

The principle of **justice** includes the responsibility to provide equitable access to the potential benefits of research. Sharing results with participants not only disseminates knowledge that can inform decisions about exposure reduction, it can also address disparities in access to knowledge.

We identified three ethical frameworks that have been used to make decisions about reporting individual results in personal exposure studies. Each gives varying weight to the ethical principles of human subjects research:

- *Clinical medicine* — an expert-driven approach, which has historically supported reporting results only when the health significance of exposures is known gives priority to preventing harm from worry,
- *Community-based participatory research (CBPR)* — a prevention-oriented approach, which emphasizes autonomy in participants' right to decide whether to learn their results and beneficence in the potential to inform constructive action even when health effects are uncertain, and
- *Citizen-science data judo* — an advocacy-driven approach, which encourages reporting individual results to support precautionary action and policy change (Brody 2007; Morello-Frosch 2009; Morello-Frosch 2005).

We believe that the best interpretation of ethical values weighs in favor of a participant's right to decide whether to know or not know individual results. At present, though, only the California biomonitoring program requires report-back, so choices about whether to report remain with researchers and IRBs.

CBPR approaches consider community and participant views, support health-protective action, and build trust

We adopted a CBPR framework, which values mutual respect and open communication, co-ownership of data, and empowerment (Brody 2007). This handbook reflects our CBPR approach.

The CBPR framework emphasizes that reporting study results can benefit participants and communities by creating access to information and empowering people to act on that information. Because reporting individual results has the potential to negatively impact communities—through stigmatization, for example—CBPR considers the rights of both individuals and communities. A CBPR approach involves collaborative decision making among researchers, study participants, and community members. Just as researchers and participants enter into a relationship to conduct the research, that relationship extends to the report-back process.

A key question, then, is whether the study community and participants want their results. Experience in studies that have asked their participants show that most do want to know (Brown-Williams 2009a; Nelson 2009; Quandt 2004; Wu 2009). For example, in our Household Exposure Study (see box), community leaders advocated for reporting individual results to participants who wanted them, despite uncertainty about the health effects of many of the contaminants measured (Brody 2007). Nearly all participants requested their results, and follow-up interviews with participants indicate that they appreciated the opportunity to receive them (Altman 2008; Brody 2007). One study participant noted:

> At first I was thinking, "God, I wish I didn't know all this." But the more I think about it, the more I understand it, the more I feel like it helps me to, ... do whatever I can...if you know the information then you can't not participate in trying to make change.

HOUSEHOLD EXPOSURE STUDY

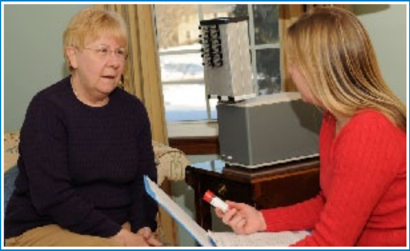
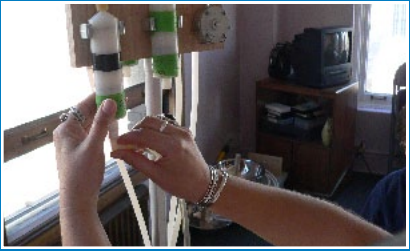
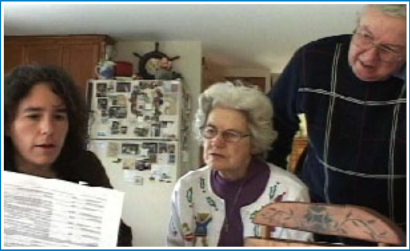

Silent Spring Institute launched the Household Exposure Study in 1999 to test indoor air and dust samples from 120 homes in Cape Cod, MA, for 89 endocrine disrupting compounds (EDCs), including phthalates, flame retardants, parabens, pesticides, alkylphenols, polycyclic aromatic hydrocarbons (PAHs), and polychlorinated biphenyls (PCBs) (Rudel 2003; Rudel 2008). Many of the target chemicals are considered emerging contaminants—chemicals for which exposures are not well documented and links to health are uncertain—and 30 of the compounds were reported by this study for the first time in indoor environments.

In 2004, Silent Spring Institute partnered with Communities for a Better Environment, a California environmental justice organization; Commonweal, a health and environment organization; and researchers from Brown University and the University of California, Berkeley, to expand the Household Exposure Study. The expanded study tested homes in Richmond, CA, an urban community bordering a Chevron oil refinery, transportation corridors, and other industry, and Bolinas, CA, a nonindustrial comparison community (Brody 2009; Dodson 2012; Rudel 2010). The team collected indoor and outdoor air and household dust samples from 50 homes. Samples were analyzed for over 150 compounds, including EDCs such as phthalates, PBDEs, parabens, pesticides, alkylphenols, PAHs, PCBs, and other estrogenic phenols, as well as metals and particulate matter (PM2.5), which are associated with industry and transportation. We interviewed study participants about their experiences learning their results (Adams 2011; Altman 2008).

In the CYGNET Study, one of the NIEHS Breast Cancer and the Environment Research Centers (BCERC), all but one of the parents expressed an interest in learning their daughters' results, even if they expressed concern about the potential implications (Brown-Williams 2009b).

A study of pesticide exposures in the homes of farmworkers in North Carolina followed a CBPR approach to communicating results, emphasizing participants' right-to-know the information (Quandt 2004). Informal interviews with participants and more structured interviews with community members about what they thought participants would want to know supported reporting individual results. The researchers argued that participants in this and other community-based research studies should receive their results because "It is ethical to return information to the 'owner' of that information." They note that sharing information builds trust among researchers, participants, and communities.

Our interviews with researchers and study participants indicate a trend in favor of communicating results to participants (Adams 2011; Altman 2008; Morello-Frosch 2009). Indeed, a growing number of personal exposure and biomonitoring studies are reporting individual results, while others are considering reporting or are in the planning phase. Examples of studies that have reported individual results are shown in **Table 1**.

ANTICIPATING THE COSTS

Implementing a communication plan can be time-intensive. Don't forget to allocate time for interpreting data, developing and disseminating materials, answering participants' questions, and maintaining regular communications with the study community. Some studies need funds for translating materials into multiple languages. Build these

TABLE 1.
SELECTED PERSONAL EXPOSURE AND BIOMONITORING STUDIES THAT HAVE REPORTED INDIVIDUAL RESULTS TO PARTICIPANTS.

STUDY	SUMMARY
CHAMACOS (UC, Berkeley, Center for Environmental Research and Children's Health), http://cerch.org/research-programs/chamacos/	A longitudinal birth cohort study that measures exposure to pesticides and other chemicals in farmworkers' children from birth to age 12 to determine if this exposure affects growth, health, or development.
Chemicals in Our Bodies Study/Maternal Infant Environmental Exposure Project (UCSF, UC Berkeley and Biomonitoring California)	A study of environmental chemical exposures in mothers and newborns conducted as a pilot project. Participants were interviewed about potential exposure sources and participated in usability testing interviews to provide input into the final report-back materials.
Community Exposures to Perfluorooctanoic Acid (C8 or PFOA) Study (Center of Excellence in Environmental Toxicology, University of Pennsylvania School of Medicine)	This partnership of environmental researchers, local health care providers in the Parkersburg, WV, area, and the Little Hocking Rural Water Association, investigated PFOA levels in residents, sources of exposure, and selected health effects. The study pioneered the Community First Communication strategies to report results to participants and community stakeholders.
CYGNET Study (Bay Area BCERC, Kaiser Permanente), http://bayarea.bcerc.org/cygnet.htm	A longitudinal epidemiologic study focused on determinants of pubertal maturation in young girls. This study measures environmental chemical exposure, development and lifestyle factors, and genetic and psychosocial factors. A wide range of results, including environmental chemical measurements, were offered to parents.
Environmental Justice for Saint Lawrence Island (ACAT)	In collaboration with the Yupik Eskimo people of St. Lawrence Island, researchers measured PCB contaminant levels in traditional subsistence foods and found high levels of PCBs in important foods including bowhead whale, walrus, and seal.
Greater Boston PBDE Body Burden Study (Boston University School of Public Health and Lowell Center for Sustainable Production at UMass Lowell)	An epidemiologic study designed to identify pathways of exposure to polybrominated diphenyl ethers (PBDEs). The study tested breast milk and house dust samples from 46 pregnant women and new mothers in the Boston area.
Growing Up Female (Cincinnati BCERC, University of Cincinnati) http://www.eh.uc.edu/growingupfemale/	A longitudinal epidemiologic study focused on determinants of pubertal maturation in young girls. This study measures environmental chemical exposure, development and lifestyle factors, and genetic and psychosocial factors. After unexpectedly high levels of PFCs were detected in some of the girls, the study reported individual results to parents.
Household Exposure Study, (Silent Spring Institute), http://silentspring.org/our-research/everyday-chemical-exposures	This tested air, dust, and urine samples in 170 homes in California and Massachusetts for about 90 endocrine disruptors and additional industrial pollutants.
La Familia Study (Wake Forest University Health Sciences), http://www.wakehealth.edu/Research/Family-Medicine/La-Familia.htm?LangType=1033	A CBPR project to develop a lay-person intervention to reduce pesticide exposure in families with a migrant or seasonal farmworker. Researchers measured pesticide levels in participants' homes; reported results, source information, and reduction strategies; and evaluated participant comprehension.

costs into the research budget when possible. If a large sample size is important, or even mandated, computerized approaches to report-back may be useful. For that reason, our team is developing and testing such approaches to support effective report-back in larger studies, such as government biomonitoring programs where individual contact with each participant may be too costly or not logistically feasible.

ROLES AND RESPONSIBILITIES

In CBPR projects, many players have important roles to play. Involving community leaders and trusted liaisons can ensure that report-back methods and messages are tailored appropriately for the study community. In addition, it is critical for the study's scientific leaders to remain responsible for interpreting data and reviewing messages to ensure their accuracy. Researchers also must make sure that everyone, including community members who are involved in reporting individual results, is comfortable with confidentiality practices and trained in human subjects protections.

Some teams may involve healthcare providers in interpreting and reporting results, particularly when the provider is known and trusted by the community. Because environmental health may be outside their area of expertise, they will need to be thoroughly briefed about the scientific context, aims, methods, and results of the study as well as the interpretation of individual results for specific chemicals. In particular, they may benefit from training in messages about precautions when health effects, sources, and exposure reduction strategies for chemicals are uncertain.

INFORMED CONSENT

For research teams that have decided up front to report individual results, communication can begin with informed consent. The principle of autonomy directs researchers to provide individuals with the opportunity to decide freely if they wish to become study participants. Extending that principle to communicating results, it follows that study participants have a right to decide whether they want to know — or not know — their individual study results. We have found that getting informed consent for study participation and report-back at the same time is effective and convenient.

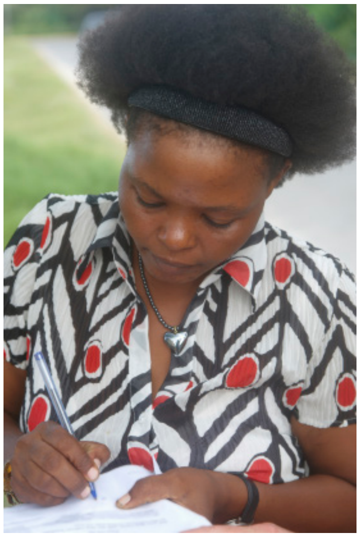

The informed consent process is a good opportunity to find out whether study participants want to receive their own results. This is also a good time to set expectations about what participants will and will not be able to learn from the study.

The Household Exposure Study told potential participants about the option to receive individual results in the informed consent form. Here is an excerpt:

> HOW WILL THE FINDINGS BE REPORTED? You will have an opportunity to learn the results for your home if you wish. In addition, a summary of the findings for all the homes together will be reported to Richmond residents in public meetings and news media. The overall research results will also be published in scientific journals. Your name and other identifying information will never be used in any reports or publications.

To help participants decide, the informed consent can clarify what information the study will and will not be able to provide. For example, in fact sheets distributed to potential participants, we communicated that the study was likely to detect pollutants in the home, but that it was not designed to find relationships between those pollutants and health effects:

> We are not able to draw conclusions about the health effects of exposure to the chemicals. Further studies would be needed to determine any links between exposures and health consequences.

Researchers can also articulate that reporting individual results — even when health effects are uncertain — can provide participants with opportunities to take action to reduce exposures.

LEGAL ISSUES

In some studies, individual results may have legal implications. For example, if an individual learns that contamination of a property could harm others, that information might need to be disclosed to those who use the property or when the property is sold. Although this circumstance would not be expected to occur in most studies, participants need to be informed during the consent process if this risk is reasonably anticipated.

In addition to the usual research practices that protect individual information, researchers can apply for a Certificate of Confidentiality, which protects the researchers themselves from forced disclosure of identifying information in federal, state, and local civil, criminal, administrative, legislative, or other proceedings (National Institutes of Health 2009). These Certificates are relatively new, so their legal limitations are not yet well understood. This Certificate does not prevent participants from voluntarily releasing information about their involvement in the research.

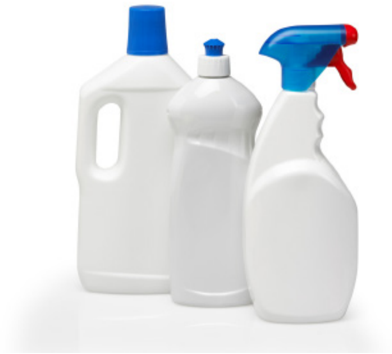

Study participants may be able to reduce exposures by switching products.

COMMUNICATING RESULTS FOR CHILDREN

Studies involving vulnerable populations — including participants with limited capacity for free consent or limited capacity to understand their results — require special protections. For example, the research team will need to decide if children are mature enough to receive study results or if results should be reported to parents or legal guardians, or some combination. If results are reported to children, tailored communications should be developed.

NAVIGATING THE IRB PROCESS

Once the communications strategy is developed, it must be approved by the study's Institutional Review Board (IRB)—a committee charged with overseeing human subjects research to ensure that the rights and confidentiality of individual research participants are protected (Penslar 1993). Because IRBs may not be familiar with CBPR, they have sometimes been reluctant to oversee community partners and to approve reporting individual results when health effects are uncertain. This can lead to substantial delays, diversion of study resources, and damage to community-researcher relationships. We recommend educating the IRB well in advance about CBPR and working to ensure that the IRB includes at least one member familiar with CBPR.

Brown University's IRB was initially hesitant to oversee researchers from the community partner organizations in our collaborative. In response, our team extensively discussed CBPR issues with the Brown IRB and demonstrated that the community partners were experienced in scientific research and trained in human subjects protection. We showed the IRB that CBPR processes were growing in importance and in federal funding. These efforts resulted in a novel agreement to cover the work of all partners in our collaborative (Brown 2010).

Similarly, in the CHAMACOS Study, a pesticide biomonitoring study with pregnant women and children in an agricultural community in California, the IRB initially objected to dissemination of individual results (Bradman 2007). The researchers organized meetings with various study stakeholders—researchers, community members, doctors, advocates, and industry representatives—during which advocates and industry demonstrated support for returning individual results. One community member expressed study participants' right-to-know, saying, "I think you will get a very positive response from the women. They are very interested in their children's health and how to improve it. You need to give them access to their results." The team was able to use this information in their efforts to educate the IRB, which ultimately led the IRB to reverse its initial decision.

Still, some researchers have found that IRBs only allow "passive" report-back in which the responsibility is on the participants to call and request their data. And some IRBs have forbidden advocacy groups from even conducting biomonitoring research in which report-back would be a component of responsive community engagement. We hope these barriers will fall as IRBs become more familiar with effective practices.

Based on our experience and research, we suggest the following strategies for research partnerships:

- *Get to know the IRB.* Becoming familiar with IRB members before the review process can help researchers assess their familiarity with CBPR and the extent of education they may need.
- *Take time to educate the IRB.* This may involve such activities as preparing memos on the history, principles, and practices of CBPR; maintaining regular contact with IRB staff through emails and in-person dialogue; demonstrating precedent by pointing to other successful projects; and inviting IRB staff to CBPR workshops or other educational events.
- *Make sure academic IRBs know community partners.* Academic researchers can connect community partners with IRB staff to demonstrate the community's involvement in the research process and how this involvement is key to the project's success. Research partners can include a description of "community consent" in their IRB application.

"YES. AT FIRST I WAS THINKING, "GOD, I WISH I DIDN'T KNOW ALL THIS." BUT THE MORE I THINK ABOUT IT, THE MORE I UNDERSTAND IT, THE MORE I FEEL LIKE IT HELPS ME TO…TO TRY TO DO WHATEVER I CAN TO MITIGATE OR ALLEVIATE THE TOXINS THAT ARE IN MY ENVIRONMENT… IF YOU DON'T KNOW THE INFORMATION THEN YOU HAVE AN EXCUSE FOR NOT BEING ACTIVE. BUT IF YOU KNOW THE INFORMATION THEN YOU CAN'T NOT PARTICIPATE IN TRYING TO MAKE CHANGE."

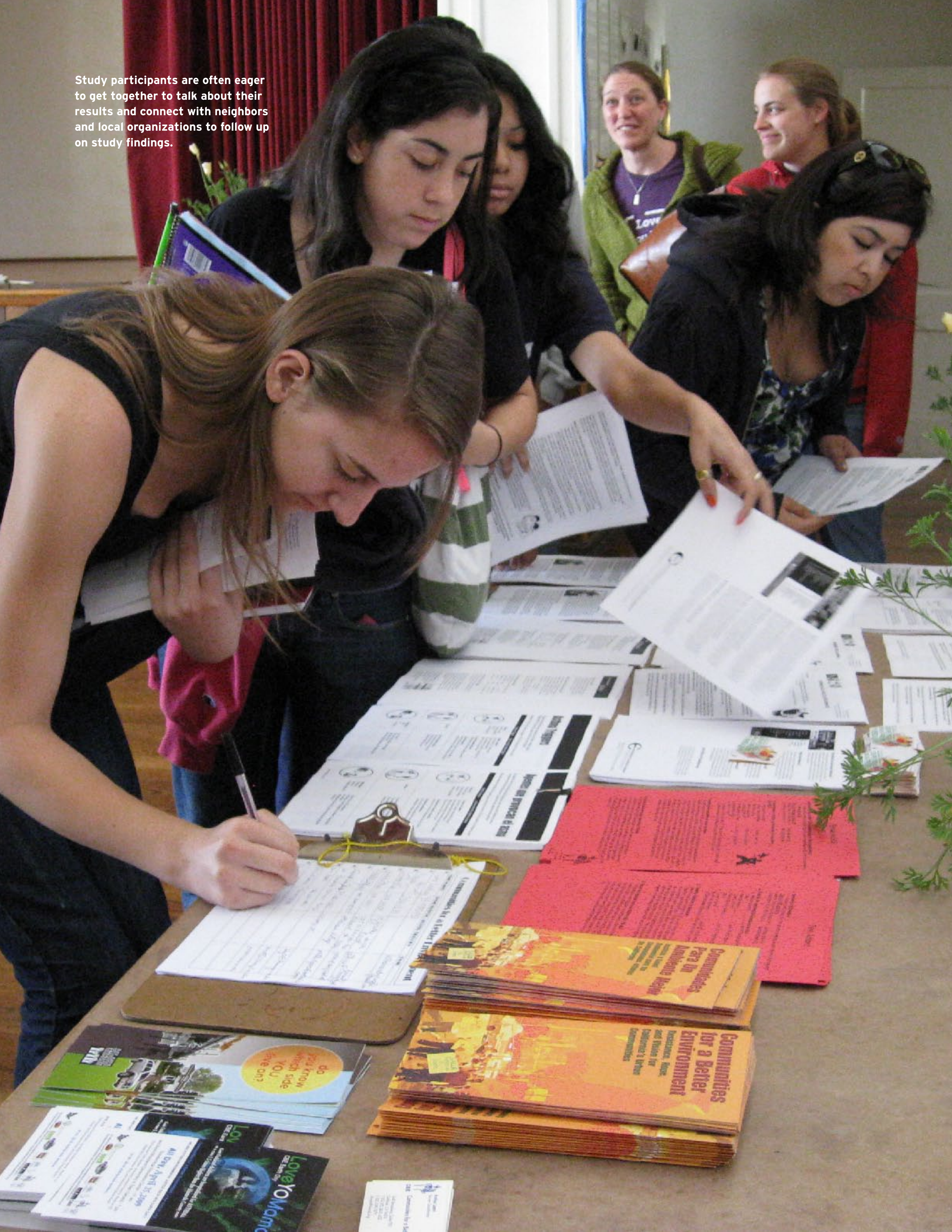

Study participants are often eager to get together to talk about their results and connect with neighbors and local organizations to follow up on study findings.

CHAPTER 2: METHODS FOR REPORTING RESULTS

MORE THAN "ONE SIZE FITS ALL"

Research teams that have decided to report individual results will then need to determine how, including the content and process of report-back. Some of the questions facing researcher teams about report-back include:

- What information do we report? All or only some of the results?
- How do we design the materials? Graphs, text, pictures?
- How do we distribute the results? Mail, in person, drop-off, or at a community meeting or clinic visit?
- When do we report results? Before or after disseminating results in peer-reviewed journals and conference presentations?
- What if there is a problem with report-back? What if a participant is upset?

The information in this chapter is intended to help guide teams through the decisions about how to report results. First steps include engaging community representatives in the process to help ensure that report-back is conducted in a way that is understandable, meaningful, and culturally appropriate. We have also found it helpful to consider principles and experiences in risk communication, such as strategies for building trust, respecting cultural context, and considering how people process information.

> "WE WERE JOINING A SMALL GROUP OF PEOPLE THAT ACTUALLY KNEW WHAT THEIR CHEMICAL BODY BURDEN WAS AND THAT THIS WAS INFO THAT WE WOULD NOT BE ABLE TO UNLEARN…IT WOULD BE A NEW KIND OF RESPONSIBILITY."

CONSIDERING PRINCIPLES OF RISK COMMUNICATION

Research on risk perception has shown that when people make judgments about risk under uncertainty they rely on various heuristics to simplify their decision-making process (National Academy of Sciences 2006; US Environmental Protection Agency 1988). Heuristics are simple, often unconscious, "rule of thumb" decision-making strategies that are practical shortcuts for complex situations and incomplete information but sometimes result in misjudgment. For example, people tend to underestimate the risk of a common hazard, such as driving an automobile, while overestimating the risk of a rare, memorable one, such as a shark attack. At the same time, people tend to judge a hazard based on how easily they can recall or imagine an event, so they may overestimate the risk of a hurricane if one has been in the news recently.

Reliance on such rules of thumb has the potential to lead participants to under- or overestimate risks associated with study findings. Although it is difficult to anticipate how participants will respond to receiving their own results, it is helpful for teams to keep these principles in mind as they develop a report-back plan. Consulting with community representatives throughout the process will give researchers a heads-up about information needs and potential misunderstandings.

DECIDING WHAT TO REPORT

When deciding what information to include in report-back materials, research teams can begin by working with community members to determine what study participants want to know. In our Household Exposure Study, we sought input from study participants, community leaders, advisory council members, and other researchers about what to report. We summarized participants' questions about their results and identified what study data would best answer those questions (Brody 2007) (**Table 2**). Over the years, we have found these questions to be an excellent reference for preparing report-back materials.

Quandt et al.(2004) also conducted informal interviews with study participants and community members to assess participants' communication needs. During these interviews, participants reported that they wanted more rather than less information, even if the health effects were uncertain:

> In terms of ambiguity, [the participants] thought it was important that scientists present "la verdad" (the truth). If this meant telling women that it was not possible to know the level of danger represented by the findings, they would prefer to know that rather than to have the scientists give a simpler, but incomplete answer.

TABLE 2.
REPORT-BACK INFORMATION THAT HELPS ANSWER TYPICAL PARTICIPANT QUESTIONS.

QUESTION	REPORT-BACK INFORMATION THAT IS RESPONSIVE
**Description**	
What did you find?	List of detected chemicals
How much?	Concentration shown in a table or graph
**Analysis/Comparison**	
Is that high?	Study participant's result shown in relation to<br>• the distribution of others in the study or<br>• a reference group, such as CDC Exposure Report
Is it safe?	Study participant's result shown in relation to<br>• a health-based regulatory guideline<br>• levels associated with health effects in epidemiologic studies
What should I focus on?	Results for tested chemicals shown in relationship to each other
Where did the chemical come from?	List of types of products or processes that commonly contain or emit detected chemicals such as combustion and auto exhaust or specific types of consumer products
**Recommendation**	
What can/should I do?	Individual and community exposure-reduction strategies, precautionary strategies, research needs

Similarly, a focus group of parents in the CYGNET Study of puberty in girls found that they wanted to receive comprehensive information about all chemicals, including chemical sources and potential health effects (Brown-Williams 2009b). The Child Health and Development Studies formed an 18-member Participant Advisory Council that meets regularly to guide this cohort study, and this group has enthusiastically discussed report-back options, considering models from earlier studies and their own values and priorities (Judd 2012).

> "AS RESEARCHERS IT'S GOOD TO REMEMBER THAT HUMAN BEINGS ARE NOT DATA POINTS… YOU ARE ENTERING INTO A RELATIONSHIP WITH ANOTHER HUMAN BEING AND YOU NEED TO FIGURE OUT WHAT THE BENEFITS ARE FOR BOTH OF YOU IN TERMS OF THIS RELATIONSHIP, ON A HUMAN BASIS, NOT ONLY ON A SCIENTIFIC DATA PRODUCING BASIS."

DESIGNING REPORT-BACK MATERIALS

Designing individual reports for personal exposure and biomonitoring studies presents significant challenges when results involve multiple media (e.g., air, dust, blood, urine), a large number of unfamiliar chemicals, or chemicals for which there are limited or no health-based guidelines or comparisons from other studies. Furthermore, participants may have varying levels of literacy and numeracy; and researchers may lack experience reporting results to non-scientific audiences.

When determining which materials and formats to use, teams need to consider the nature of the study data, key messages, the preferences of the study community, and what resources are available. Report-back materials may include graphs, text, pictures, video, DVDs and other media or a combination. Some studies may present opportunities to experiment with new media, such as interactive web-based tools; however, these formats may raise additional challenges to protecting confidentiality. Engaging community members or trusted representatives can help teams design materials that are appropriate. Teams can assess participant preferences through focus groups, community meetings, conversations, surveys, or other methods.

Combining graphs and text

Based on our experience, report-back that includes a mixture of information-rich graphs and brief verbal summaries allows for individual differences in the ability and desire to understand report-back information. Some participants prefer text summaries while others are more comfortable with graphs and images. Keep in mind that well-designed graphs rely on people's natural ability to judge above/below and larger/smaller relationships and can depend less on literacy and numeracy than text or tables.

We used a combination of materials in our Household Exposure Study. Results were presented to participants in packets (**Appendix A**) that included:

- Cover letter that introduces report-back and reviews the goals of the study
- A half-page narrative summary of key results and exposure reduction implications for the participant's home
- One-page guide to reading the graphs (**Figure 1A**)
- Graphs showing chemical concentrations in the participant's home compared with other homes in the study and Environmental Protection Agency (EPA) health guidelines, where available (**Figure 2**)
- Graph of key community results: comparing chemical concentrations in the two study communities
- Table of information on potential sources of each chemical
- One-page summary of study goals, methods and contact information for the study team
- Summary of potential sources and health effects of chemical classes in the study
- Fact sheets about exposure reduction strategies tailored to the study community and participant's results (e.g., information about Integrated Pest Management (IPM) or low-emission wood stoves).

FIGURE 1.
EXAMPLES OF GUIDES THAT CAN BE USED AS A ROADMAP FOR INTERPRETING GRAPHICAL PRESENTATIONS OF INDIVIDUAL EXPOSURE DATA, DATA FROM OTHER PARTICIPANTS, NATIONAL AVERAGES, AND BENCHMARKS.

A) A GUIDE FROM THE NORTHERN CALIFORNIA HOUSEHOLD EXPOSURE STUDY.

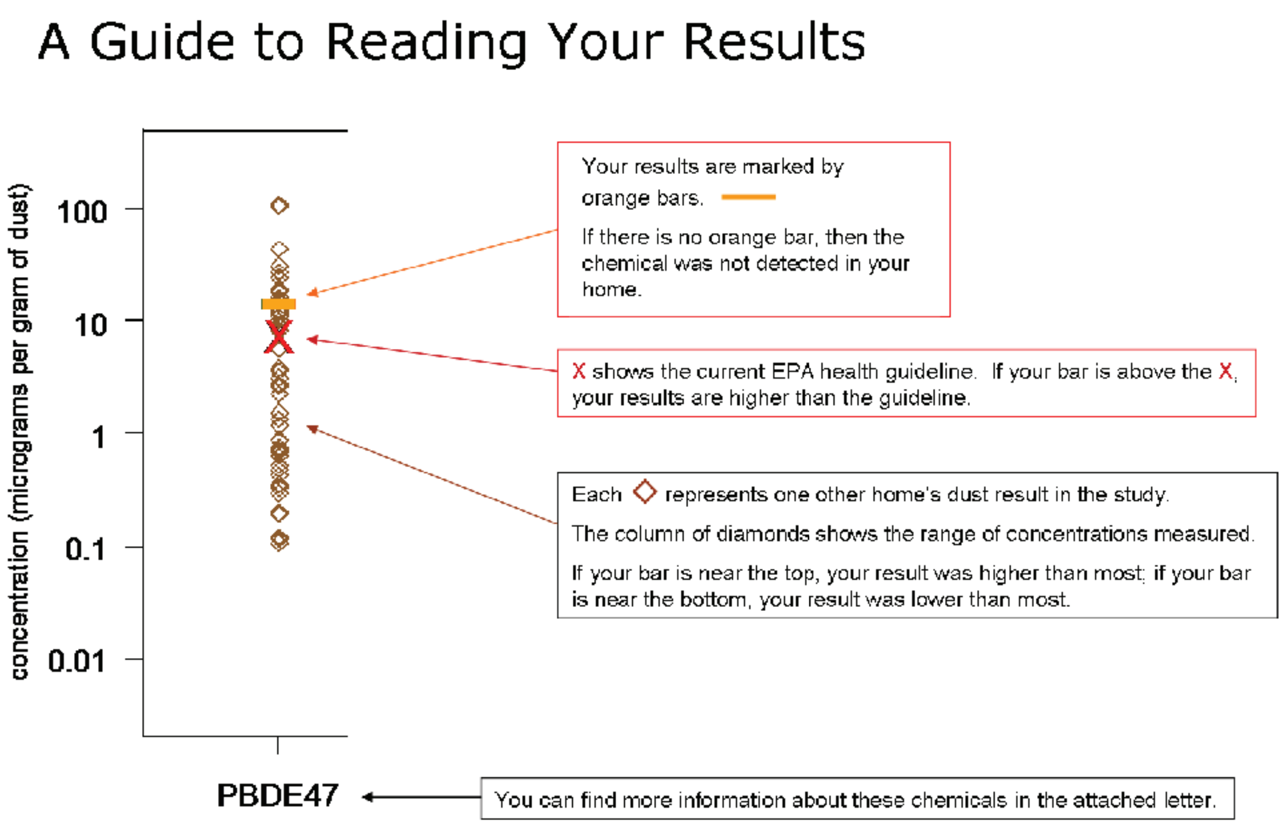

FIGURE 1.

B) A GUIDE FROM THE CHEMICALS IN OUR BODIES STUDY.

How to Read the Results Charts for You and Your Baby

There are xx Results Charts. The charts show the amount, or level, of each chemical found in your blood or urine or in your baby's blood

Some charts show results for both you and your baby. Some show the results for you only. This is because we tested both blood and urine samples from mothers, but only blood samples from babies.

What the Symbols Mean:

National average
This is the average level found in pregnant women in the U.S.

Benchmark
Levels close to or above the benchmark are a health concern. Benchmarks do not appear in most charts, because they have not been established for most chemicals that we measured.

Your result
This is the amount found in your sample. If there is no blue circle, that chemical was not found in your sample.

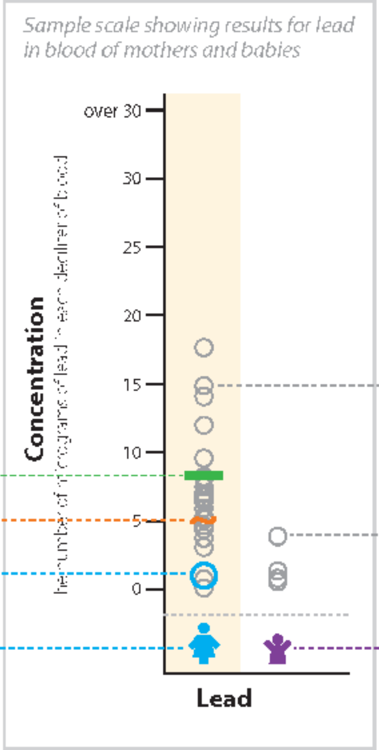

Other people's results
Results for the other mothers and babies in the study.

Your baby's result
If there is no purple circle, that chemical was not found in your baby's sample. Charts about tests in urine do not have results for babies. That is because babies' urine was not tested.

Understanding Averages and Benchmarks

You can compare your results to the levels for other people in this study or for pregnant women nationwide. The national averages are taken from the National Health and Nutrition Examination Survey (NHANES). This survey was conducted by the Centers for Disease Control and Prevention, a federal agency.

You can also compare your results to benchmarks when they exist. Benchmarks are established by different government agencies, such as the U.S. Environmental Protection Agency (EPA) and California's EPA. Levels close to or above the benchmark are a possible health concern. Benchmarks have not been established for most chemicals that we measured.

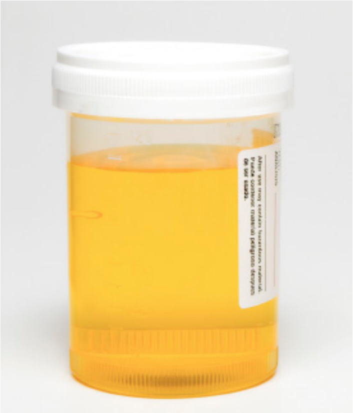

Biomonitoring allows study participants to learn that chemicals in the environment and everyday products end up in their blood, urine, finger nails, breast milk, and other tissues.

Graphs and tables

Graphs and tables are efficient ways to present quantitative data and the comparisons that give the results meaning.

Tables are useful for listing precise results or comparing one result to a health guideline (Few 2004). For participants used to receiving medical results, table formats may be familiar. They are also compact for reporting large numbers of findings.

Graphs are preferred for communicating patterns in data (Few 2004). As a result, graphs are useful for presenting results for chemicals for which there are limited or no health guidelines and interpretation is based on distributions and comparisons of results, for example, across media, locations, and chemicals. In addition, graphs avoid some of the limitations of tables and text, which are more dependent on literacy and numeracy (Few 2004). Graphs communicate basic concepts, such as larger/smaller, above/below, and many/few that correspond to key study messages. The specific type of graph used will depend on the information being communicated as well as participants' preferences. We have included a variety of graphs as examples below.

Our team has experimented with several types and versions of graphs, informed by the type of data in the Household Exposure Study and feedback from community members and study participants. Selecting and developing appropriate graphs has been an iterative process and continues to be a work in progress.

Graphs should be designed to be self-explanatory. In our experience, though, participants who think of themselves as unskilled with numbers are reassured when we present a "how to read the graph" guide before presenting individual results graphs (**Figure 1A and 1B**). While the first reactions of some professional risk communicators and environmental literacy practitioners consider our graphs to be "too difficult," we have observed community members in many different settings reading them successfully. We think this is because they are visual communications —"pictures" — rather than relying fundamentally on literacy or numeracy. Since we began using these methods, participants and community members affiliated with other studies have also found that graphs worked well to communicate results. Sometimes it takes a few minutes for study participants to begin reading the graphs, because they don't have confidence in their science competency. We are developing strategies to help break down confidence barriers and build environmental health knowledge.

When studies have very large numbers of results to report, graphs may be used to convey the most meaningful or important results, accompanied by tables to make reports comprehensive. We have sometimes graphed "indicator" chemicals to represent a type of exposure.

Examples of individual results

We used **simple strip** plots in the Household Exposure Study to communicate how much of each chemical was found in a given sample compared to other study participants and a health-based guideline when available (**Figure 2**). Variations of this basic approach have been used successfully in several studies. Additional modifications that address the difficulty people have with logarithmic scales would be a further advance.

In another example, the CYGNET Study used strip plots to report levels of environmental chemicals in participants' blood and urine (**Figure 3**). Each participant's value is compared with other study participants and a reference value.

Bar charts are also useful for displaying individual results relative to other participants and guidelines, and across different media or communities. For example, an advocacy biomonitoring study of consumer product chemicals in volunteers in seven states used bar charts to report on levels of bisphenol A (BPA) in participants' blood and urine (**Figure 4**).

For studies involving data collected over time, **line graphs** may be appropriate to communicate rates and magnitudes of change in levels of target chemicals.

Shaded grids may be useful for communicating values such as detect/no detect or safe/unsafe. For example, Quandt et al (2004) used a grid to display what and how many pesticides were detected in households in their study (**Figure 5**). These graphs show pesticides in columns and households in rows, with shaded cells indicating if a chemical was found in a participant's home.

Shading can also be used to convey concepts like "high, medium, low" or "safe, unsafe." We used the traffic-light colors red, yellow, and green to indicate to Cape Cod residents whether nitrate measured in their drinking water was similar to natural background levels (green), approaching a level of concern (yellow), or above the regional health guideline (red) (**Figure 6**).

Study-wide results

Graphs of study-wide results help participants understand the overall findings and put their own results in context. These graphs communicate important environmental and health messages and help participants understand how their own data contributed to knowledge.

To show community-level comparisons between Richmond, an urban environmental justice community, and Bolinas, a rural comparison, in the Household Exposure Study, we have used box plots. **Box plots** are useful for interpreting the distribution of a dataset. They quickly communicate "typical" results represented by the "box" and show the distribution of outliers, represented by the confidence interval lines and dots, sometimes called "whiskers." (**Figure 7**)

However, based on feedback we received from participants and community members who found the box plots difficult to understand, we are currently experimenting with using **strip plots** for comparisons between communities (**Figure 8**). In this example, individual results are "jiggered" horizontally, so that they don't lie on top of each other. This can be accomplished in standard graphing software.

A **cumulative bar** chart was helpful for comparing the number of chemicals at higher levels across the two communities (**Figure 9**).

In another example, the Growing Up Female Study in Ohio used strip plots to show dramatic differences in blood levels of PFOA in two communities. The graph shows individual results relative to other participants and the national average (**Figure 10**).

Another graph showed averages for each community relative to workers with high exposures, the national average, and girls from the San Francisco Bay Area (**Figure 11**).

"…THE THING THAT I WAS REALLY SURPRISED ABOUT WAS THAT THEY FOUND DDT HERE. IN THE HOME. I DON'T KNOW WHETHER IT WAS IN THE VACUUMING OR WHETHER IT WAS IN THE AIR…BUT THAT REALLY SURPRISED ME BECAUSE IT WAS OUTLAWED IN 1972 I THINK, AND WE GOT THIS LAND IN 1980 OR 1982, AND SO THAT REALLY SURPRISED ME. ALL OVER THE CAPE THEY HAD HAD A LOT OF FARMING HERE, SO I GUESS IT JUST HANGS AROUND FOR A LONG LONG TIME."

FIGURE 2.
STRIP PLOT USED TO COMMUNICATE LEVELS OF PHTHALATES, FLAME RETARDANTS, AND PCBS MEASURED IN DUST IN A HOME IN THE HOUSEHOLD EXPOSURE STUDY (RUDEL 2010).

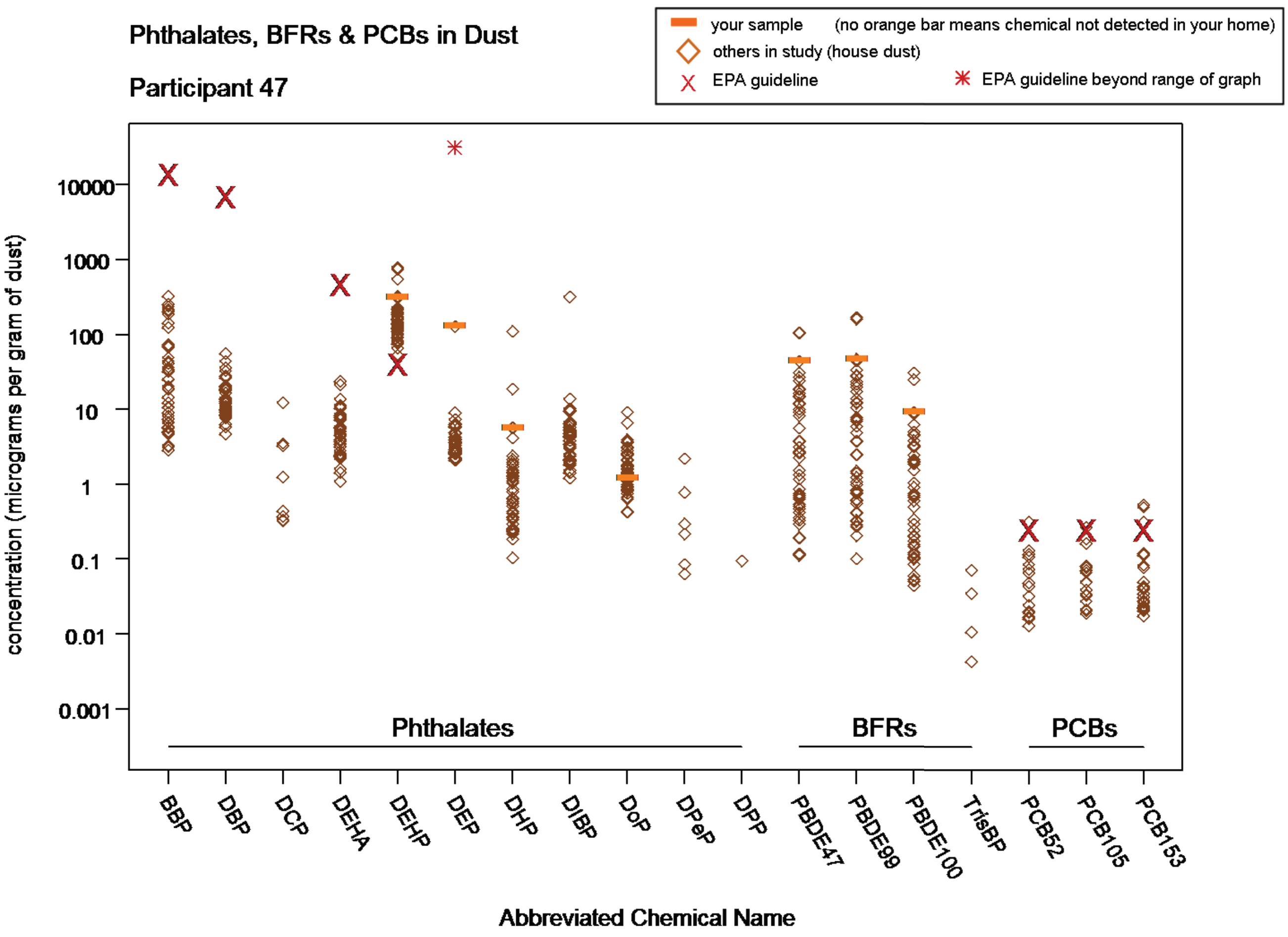

FIGURE 3.

A STRIP PLOT COMPARING INDIVIDUAL LEVELS OF BROMINATED FLAME RETARDANTS (PBDES) TO LEVELS IN OTHER PARTICIPANTS OF THE SAME STUDY AND TO THE NATIONAL AVERAGE (BROWN-WILLIAMS 2009B).

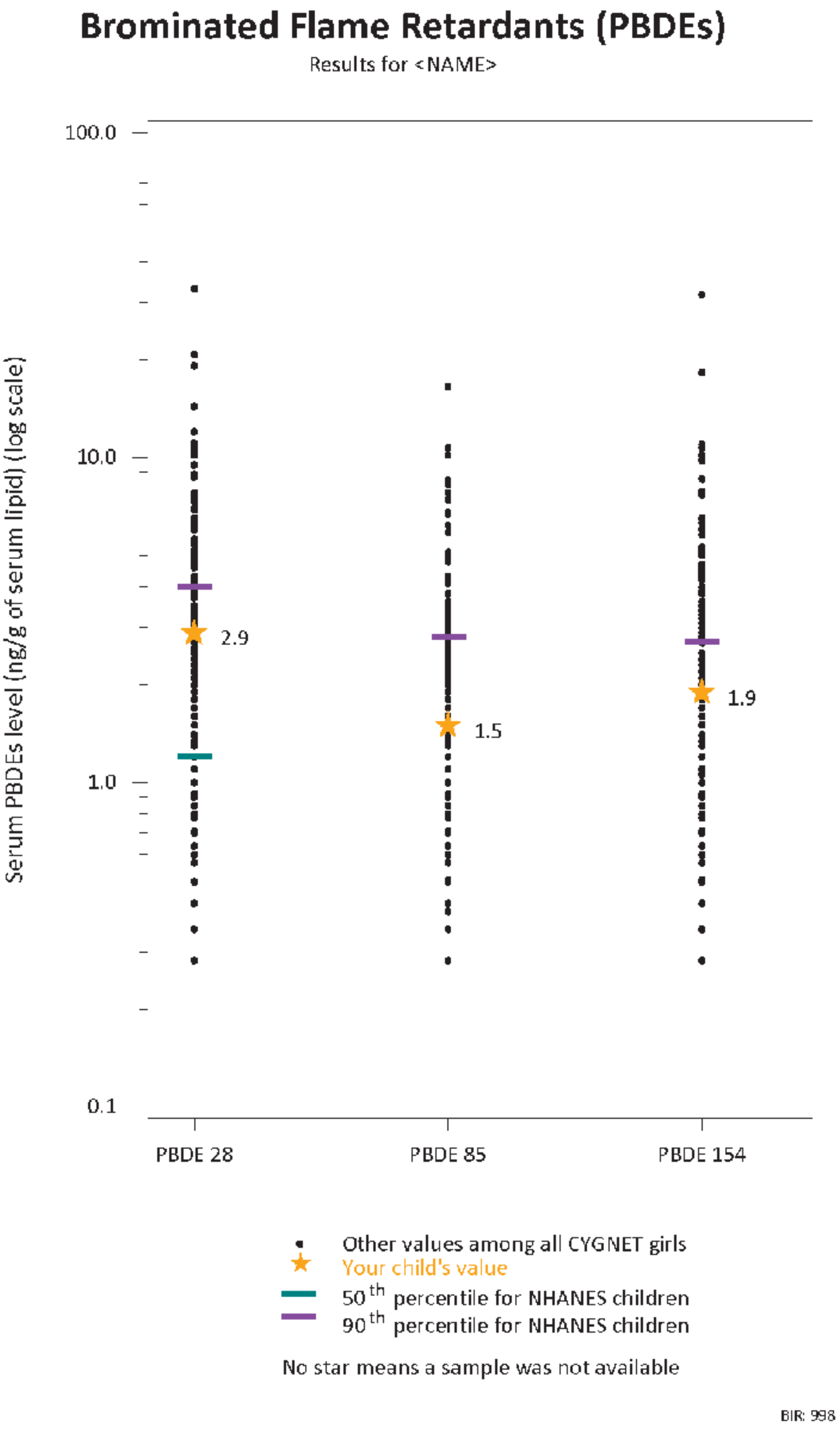

FIGURE 4.

BAR CHART REPORTING INDIVIDUAL LEVELS OF BISPHENOL A IN PARTICIPANTS' BLOOD AND URINE RELATIVE TO THE NATIONAL MEDIAN FOR BISPHENOL A (COMMONWEAL BIOMONITORING RESOURCE CENTER AND THE BODY BURDEN WORK GROUP 2007).

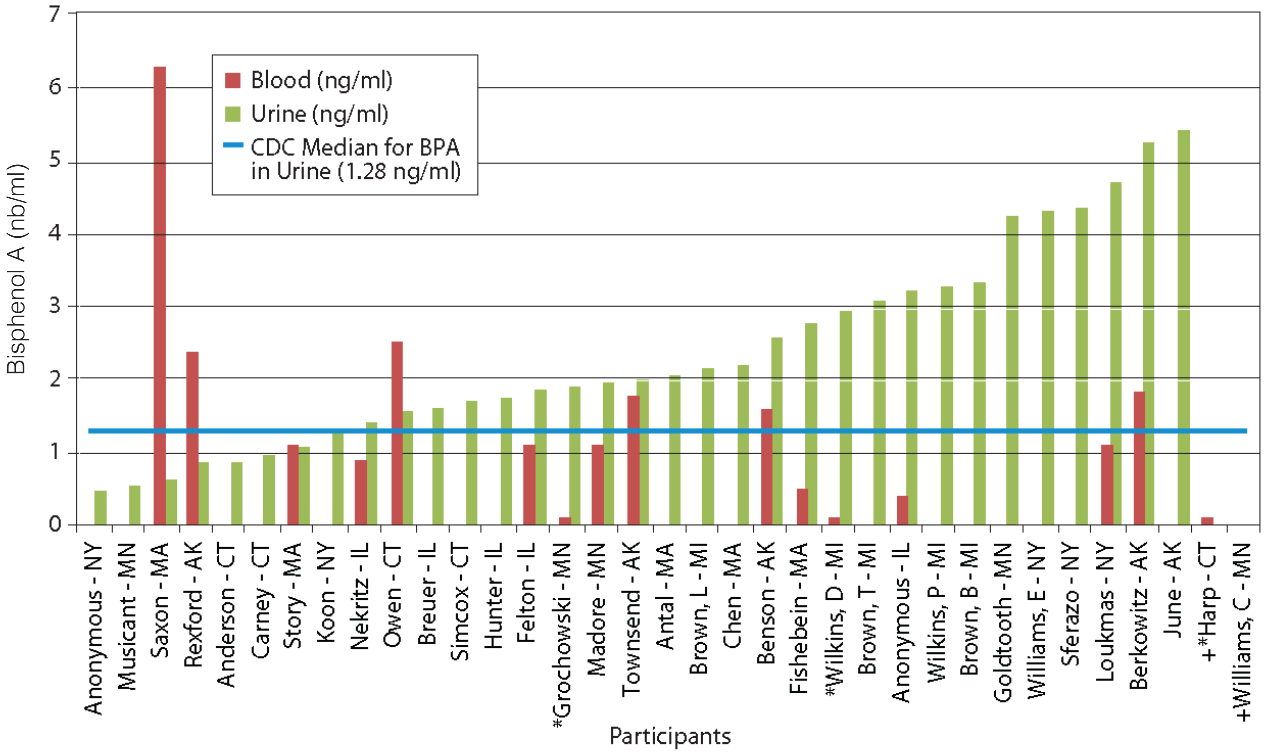

* Indicates participants's blood had detectable, but not quantifiable, levels of BPA.

\+ Indicates participants did not contribute urine samples and therefore have no BPA urine results.

FIGURE 5.

SHADED GRID SHOWING THE NUMBER AND TYPES OF PESTICIDES FOUND IN STUDY PARTICIPANTS' HOMES. THIS GRAPH INCLUDED A KEY ON THE REVERSE SIDE INDICATING WHICH AGRICULTURAL PESTICIDES WERE REPRESENTED BY EACH HEADING (QUANDT 2004).

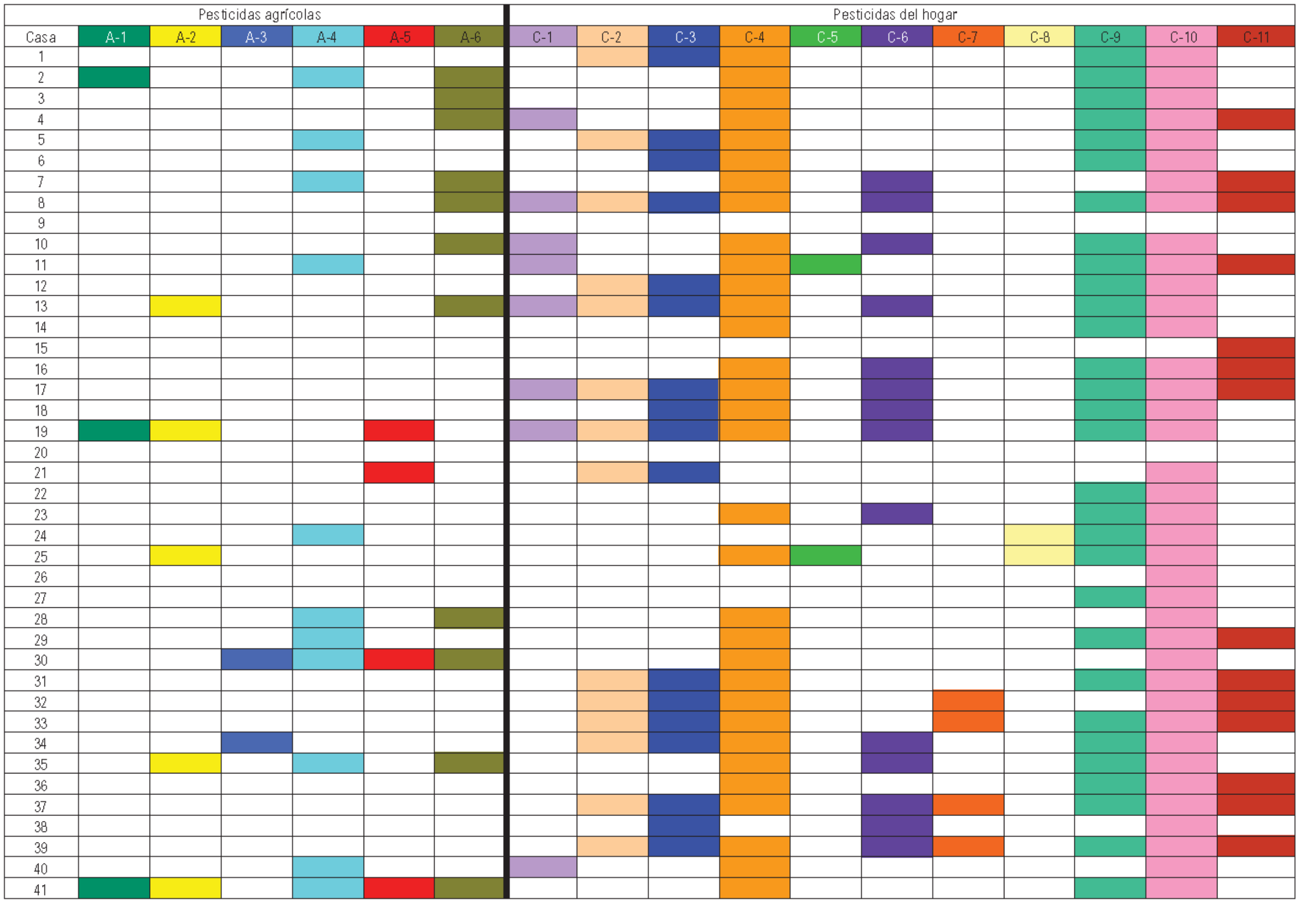

FIGURE 6.
REPORTS IN THE CAPE COD DRINKING WATER STUDY USED COLORS TO SHOW CONTAMINANT LEVELS ABOVE NATURAL BACKGROUND LEVELS OR ABOVE A GUIDANCE LEVEL.

Sodium

Sodium concentration in your water sample	US EPA's Guidance Level
26 mg/L	20 mg/L

The Guidance Level is a guideline developed by the US EPA for people with restricted sodium intake. This guideline level is not intended for the general population.

What do the colors mean?

- Above US EPA's guidance value for low sodium diets
- Below US EPA's guidance value for low sodium diets

The sodium concentration in your water sample was 26 mg/L, which is above the EPA's Guideline Value for people on low sodium diets of 20 mg/L. There is currently no guideline for sodium in drinking water for the general population.

Mercury

Mercury concentration in your water sample	US EPA's Maximum Contaminant Level
0.2 ng/L	2000 ng/L

US EPA's drinking water standard, called the Maximum Contaminant Level, is the highest concentration of mercury that the US EPA allows in public drinking water supplies.

What do the colors mean?

- Above US EPA's drinking water standard
- Above levels that occur naturally
- Within range of levels that occur naturally

The mercury concentration in your water sample was 0.2 ng/L. This concentration is well below the EPA's Maximum Contaminant Level of 2000 ng/L. Mercury occurs naturally in Cape Cod groundwater at around 3 ng/L or below.

FIGURE 7.

BOX PLOTS COMPARING LEVELS OF FINE PARTICULATE MATTER ($PM_{2.5}$) IN TWO STUDY COMMUNITIES IN THE NORTHERN CALIFORNIA HOUSEHOLD EXPOSURE STUDY. THESE AGGREGATE RESULTS CAN ALSO BE COMPARED TO BENCHMARKS TO PROVIDE ADDITIONAL CONTEXT FOR INTERPRETING RESULTS.

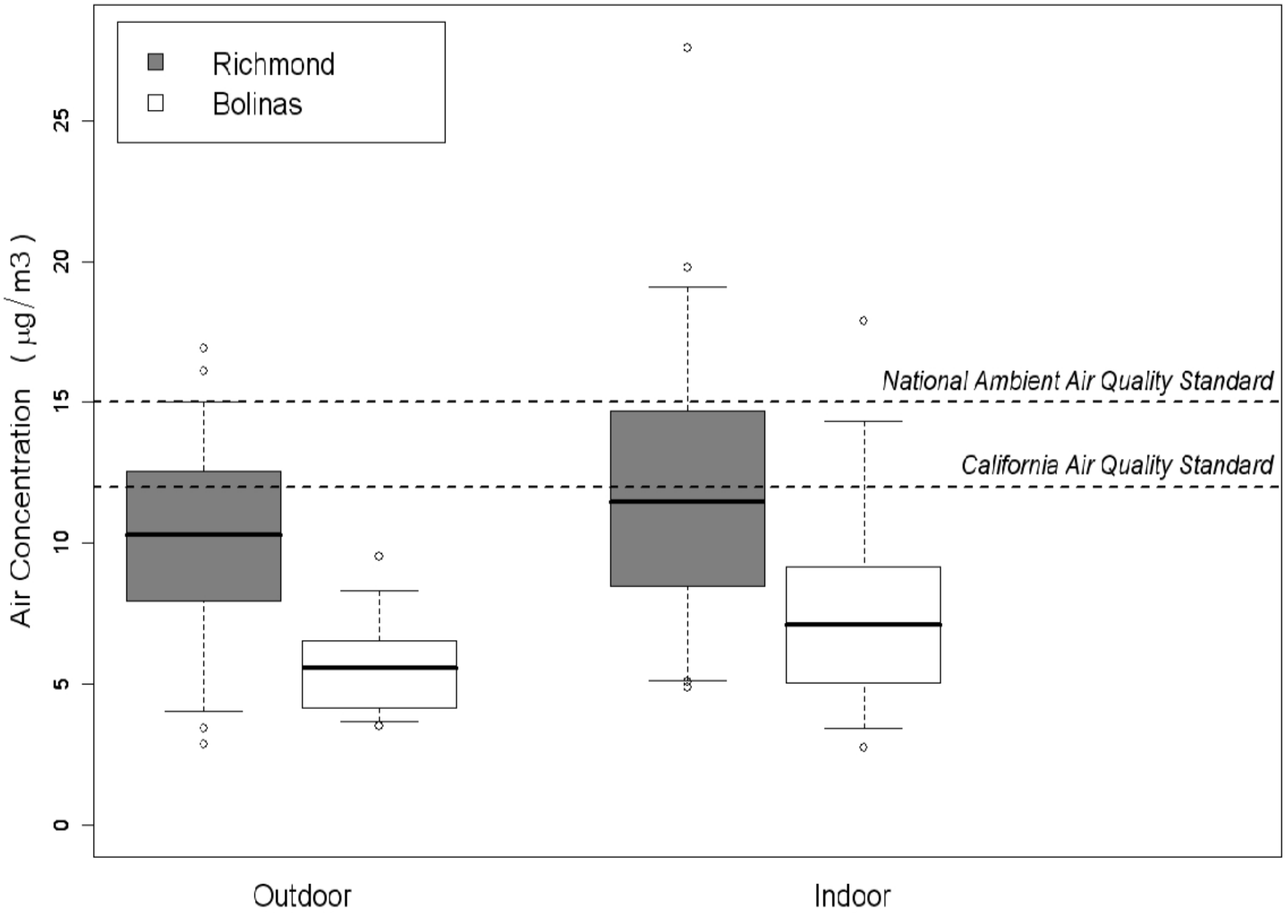

FIGURE 8.
STRIP PLOTS COMPARING LEVELS OF FINE PARTICULATE MATTER (PM2.5) IN TWO STUDY COMMUNITIES IN THE NORTHERN CALIFORNIA HOUSEHOLD EXPOSURE STUDY.

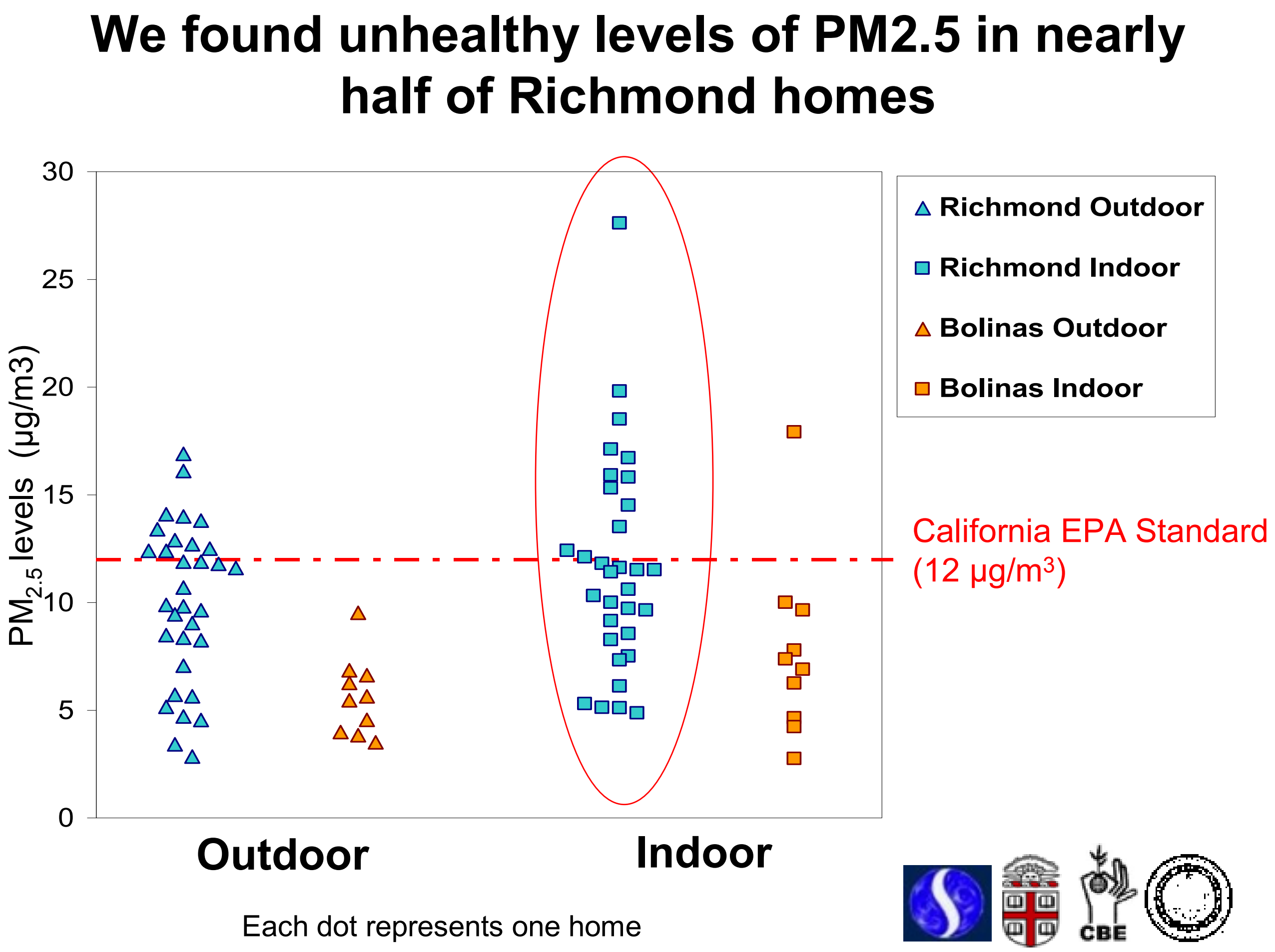

FIGURE 9.
A CUMULATIVE BAR GRAPH COMPARING THE NUMBER OF COMPOUNDS DETECTED IN OUTDOOR AIR IN TWO STUDY COMMUNITIES IN THE NORTHERN CALIFORNIA HOUSEHOLD EXPOSURE STUDY.

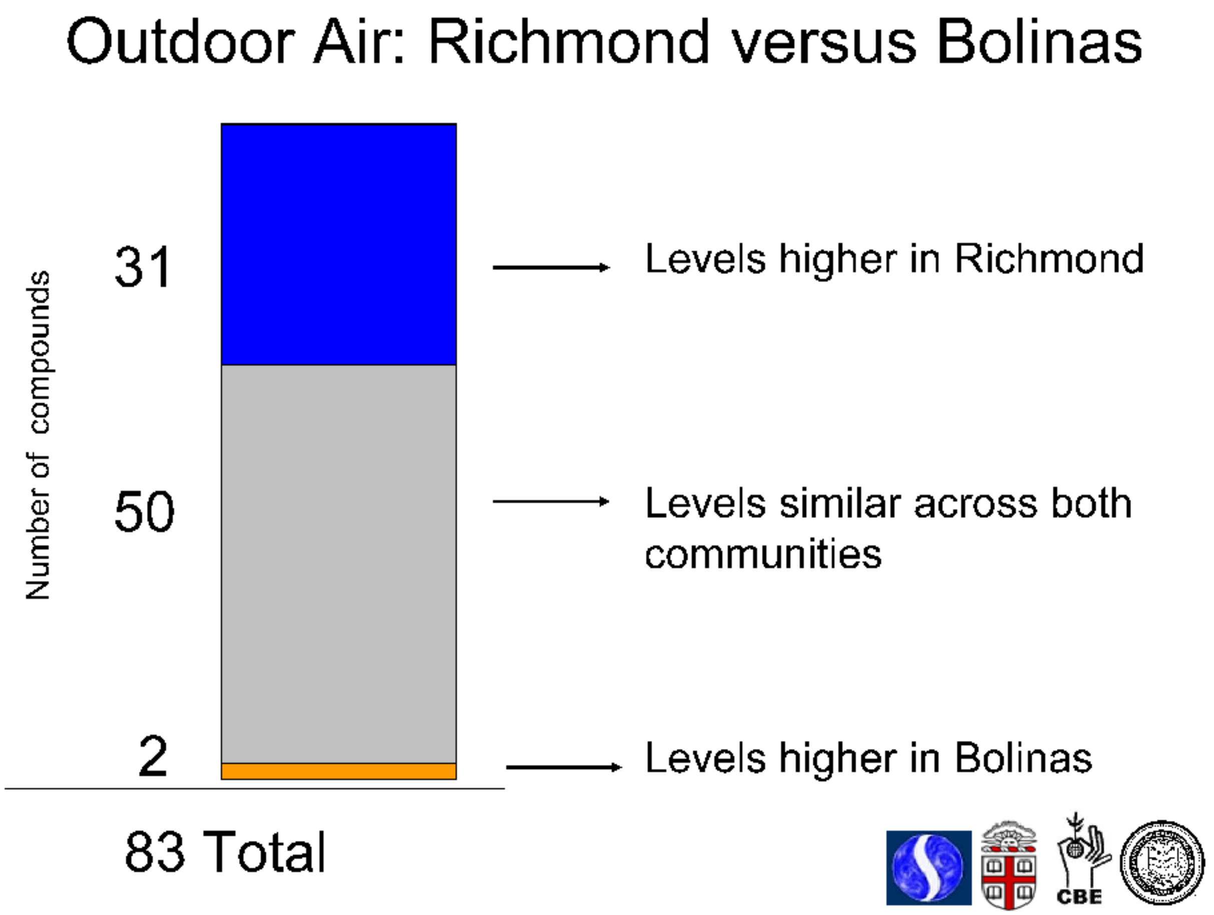

FIGURE 10.
STRIP PLOT REPORTING INDIVIDUAL BLOOD SERUM LEVELS OF PFOA RELATIVE TO OTHER STUDY PARTICIPANTS AND THE NATIONAL AVERAGE IN THE GROWING UP FEMALE STUDY (HERNICK 2007).

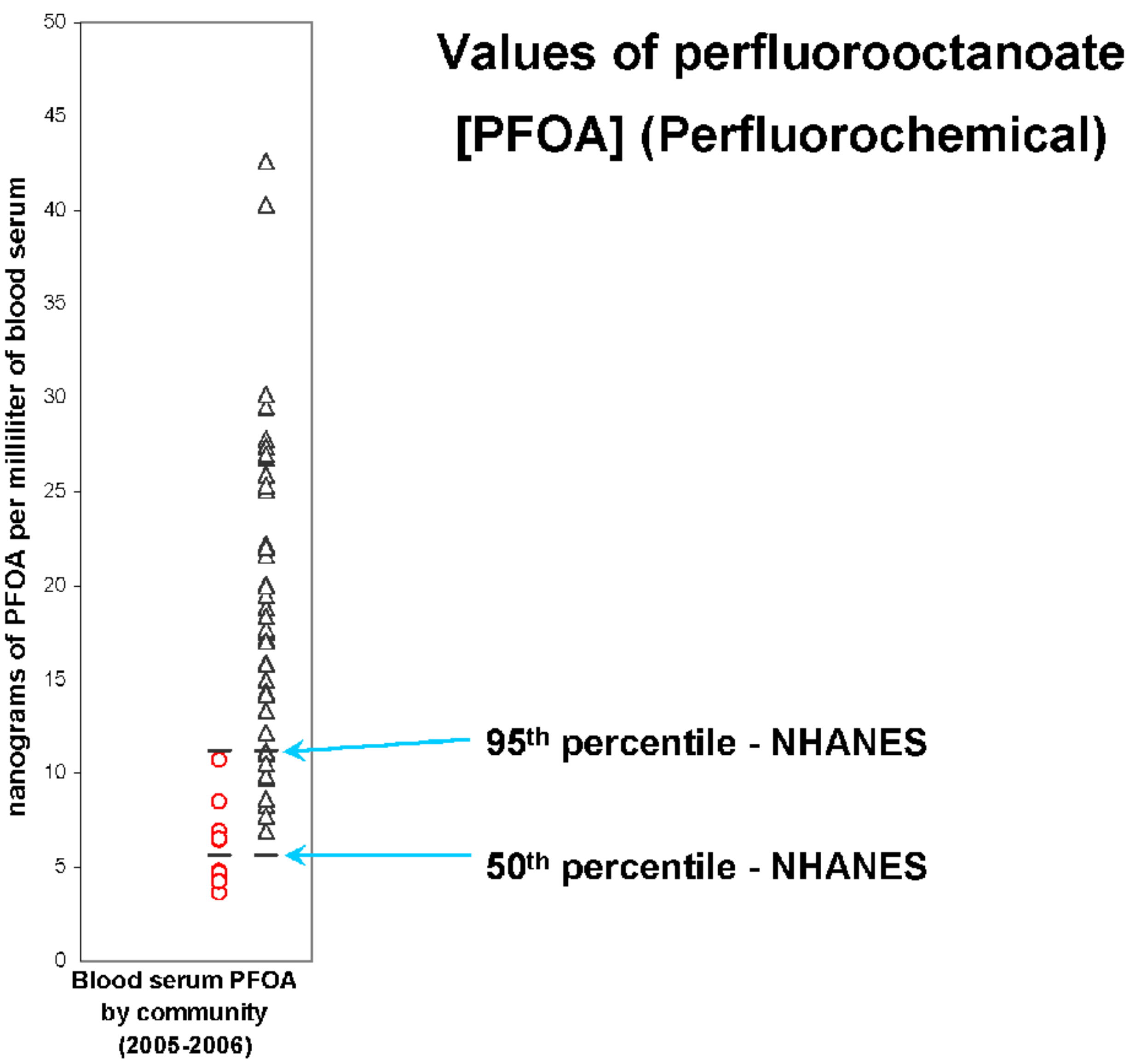

FIGURE 11.
GRAPH REPORTING AVERAGE BLOOD SERUM LEVELS OF PFOA FOR TWO STUDY COMMUNITIES RELATIVE TO WORKERS WITH HIGH EXPOSURE, THE NATIONAL AVERAGE, AND A SAMPLE FROM THE SAN FRANCISCO BAY AREA (GROWING UP FEMALE STUDY) (HERNICK 2007).

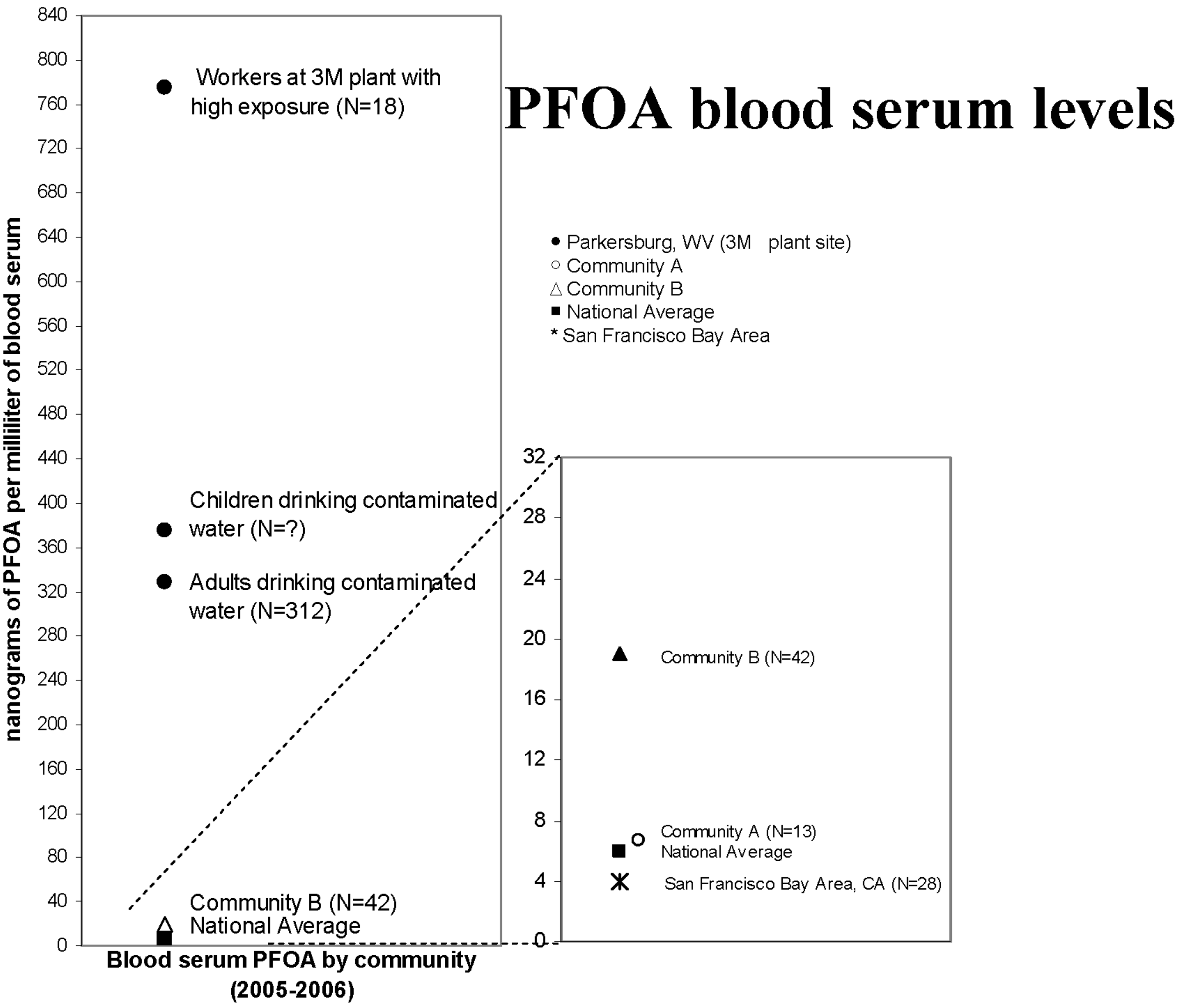

> "SHE SORT OF PUT ME AT EASE IN A WAY THAT THESE ARE CHEMICALS IN THE ENVIRONMENT AND SOME OF THEM PERSIST FOR MANY YEARS AND EVEN IF I DID CLEAN BETTER THEY WOULD STILL BE IN THE AIR. SO, YOU KNOW, IT'S JUST HELPED ME UNDERSTAND THE REALITY OF THE ENVIRONMENT I LIVE IN."

Put results in context with comparisons

Many of the example graphs include comparisons to other studies or to benchmarks. Incorporating risk comparisons like these in report-back materials can help participants contextualize unfamiliar information and inform decision-making. By benchmarks we mean guidelines such as those established by federal agencies like the U.S. EPA. Levels approaching or exceeding benchmarks may signal potential health concerns. While benchmarks may be useful when available, official health guidelines have not been established for many emerging contaminants. In addition, some guidelines that exist are outdated, so they do not reflect current science. They may even be confusing, because they don't take into consideration the study's research hypothesis. In these situations, it can be difficult to decide whether to show benchmark levels.

For personal exposure studies involving chemicals with limited or no health-based guidelines to use as benchmarks, individual results can be compared with the study population, or a reference group, such as the National Health and Nutrition Examination Survey (NHANES), which reports on chemical exposures in a representative sample of the US population. While useful, these comparisons have the potential to lead participants to over- or under-estimate risks or to misinterpret reference group levels as safety benchmarks (Brody 2007). For example, a participant who discovers that the level in her home falls below the study average may interpret her result as "safe," whereas another participant may be concerned to find his value falls at the upper end of the study distribution, even if the entire distribution falls below a benchmark. To clarify the interpretation for participants, research teams should define the reference levels used to ensure that they are not confused with regulatory benchmarks.

Highlight key messages with text

While graphs are useful for visualizing data, verbal summaries can draw attention to key messages from the graphs, help participants contextualize their results, and share with participants the researchers' expert judgment. This is particularly important in studies that are testing for numerous analytes and in different media (e.g. dust and air, or urine and blood), because the short summaries draw attention to what's most important. Reports to participants in our studies included a half-page to one-page narrative summary of results and exposure reduction strategies for the participant's home (**Appendix A**).

For example, one participant's summary had the following information about flame retardants:

> Your house contained PBDE flame retardants. The PBDEs in your house dust were generally higher than most others in the study (See page 9, PBDE). PBDE flame retardants are in foam furniture and cushions, and synthetic carpets. These chemicals were banned in Europe because of effects on thyroid hormones.

Another participant's summary placed the participant's result in the context of the other participants and a benchmark:

> You also had the highest level of lead (Pb) in your outdoor air, but this level was much lower than the EPA lead standard (page 2, Pb).

The verbal summaries included practical steps for reducing exposures:

> We found two insecticide ("bug killer") ingredients in your indoor air (page 2) and dust (page 8). You can reduce your exposure by controlling indoor pests with bait traps and other less toxic methods.

We found it useful to have team members work together to develop prototypes of the short summaries and to have each team member write up some of the short summaries, which we then compared, in order to check our consistency concerning both style of presentation and choice of what content to emphasize.

We included fact sheets about the study (**Appendix A**) and the chemical classes (**Appendix A**) in report-back packets to provide further contextual information. The study fact sheet communicates information about the goals and methods of the study, who is conducting the study, how the results are reported, and how participants can contact the study team if they have additional questions. The chemicals fact sheet explains potential sources and health effects, and names an example from each chemical class in the study. These early examples of our work have been helpful to teams who have developed their own report-back protocols. It is important to keep in mind that materials development is an iterative process as communication methods continue to improve.

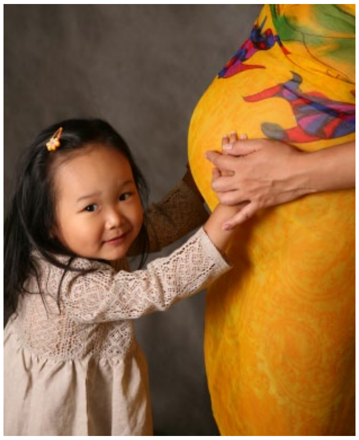

Studies involving children or samples taken during pregnancy raise special issues for reporting results. The CYGNET Study, Chemicals in Our Bodies, and Growing Up Female offers successful models.

Address the need for information about actions to reduce exposure

The report-back process is an opportunity to provide information about strategies for reducing exposure to target chemicals. Like health information, exposure reduction information should be based on the strength of evidence, and researchers need to be clear about when more research is needed before recommendations can be made (Brody 2007).

In two pesticide exposure studies, participants reported that information about exposure reduction was the most important part of the report-back process (Morello-Frosch 2009). Participants in our studies also expressed similar priorities:

> And that's what I would want from this study is give me something I can do about it. Don't just give me information that tells me I have problems....Because that's frustrating, you know? But I'm proactive enough that I'll say, "Ok, I have this information now it's up to me to do something. It's not enough for you to do it for me but just to give me some options of what I can do to change it." That, I would think, with this study would be the most important thing....

Individual actions

To inform exposure reduction, we included information about possible sources of target chemicals and exposure reduction strategies in the verbal summaries (**Appendix A**), a detailed chemical sources table (**Appendix A**), and fact sheets. The chemical sources table was designed to help participants identify the products and practices that could produce the chemicals detected in their home. During report-back consultations and at annual community meetings, team members provided handouts about exposure reduction strategies based on the participants' study results and community context. For example, in response to detecting pesticides, we provided handouts about Integrated Pest Management (IPM). Other handouts included information on wood burning stoves and flame retardants. In response to community members' interest in exposure reduction related to cleaning products and practices, we developed a "Greening your cleaning" fact sheet (**Appendix C**).

Community-level and policy action

While providing information about individual exposure reduction strategies is useful, it is also important to offer recommendations for community and policy level actions. We told participants in our study how a particular chemical is regulated in the US and in Europe and provided information about our community partners' advocacy campaigns (**Appendix D**). During community meetings, participants brainstormed creative ways to apply study findings to grassroots organizing, including using individual study

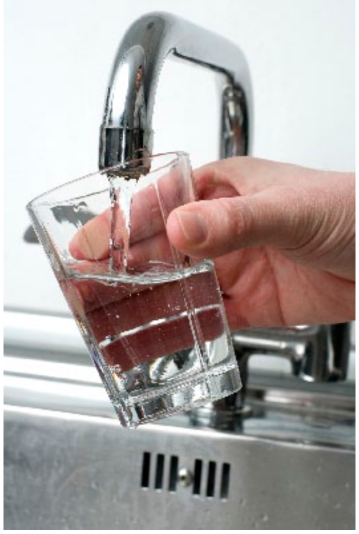

Study participants are often surprised to learn that drinking water contains both regulated and unregulated contaminants.

results in testimony at city council hearings about the proposed expansion of a nearby oil refinery. This demonstrates that report-back can be valuable even for those residents who are not participants in the study itself, since their community as a whole is affected. This value-added involves both the education about contaminants and the collective motivation to take action.

Getting comfortable with uncertainty

Although providing information about action can help participants reduce their individual exposures and leverage results to support advocacy efforts, the health effects of target chemicals may be uncertain; and interventions can be costly, inconvenient, and difficult to implement. Furthermore, there is a dearth of evidence-based information about the efficacy of many exposure reduction strategies. As a result, we caution researchers to acknowledge this uncertainty and not allow the wish to "fix" things and reduce worry to lead to unsubstantiated reassurance or recommendations. We developed a conceptual graph (**Figure 12**) (Brody 2007) to help shape recommendations for action based on how much is known about a chemical's health effects and exposure reduction strategies. Increasing certainty about health effects and exposure reduction leads to clear recommendations for individual and community level action, whereas decreasing certainty leads to recommendations for further research and precautionary exposure reduction.

Consider varying levels of literacy and numeracy

Personal exposure studies may involve participants with varying levels of literacy, numeracy, and environmental health science knowledge. Populations with lower levels of scientific literacy are as interested in receiving their individual data as are more educated groups and are able to grapple with uncertainty (Adams 2011; Brown-Williams 2009a; Morello-Frosch 2009; Quandt 2004). To successfully communicate personal exposure results to participants with lower levels of literacy and numeracy, teams can incorporate basic health communication practices, work with community members to assess participants' needs, and pilot test materials to ensure they are understandable, engaging, and relevant.

Our approach is to rely as much as possible on people's basic capacities and common sense, de-emphasizing skills and information learned in school. Nearly everyone learns to interpret spoken language and visual relationships in early childhood. We have found that using graphs and creating opportunities for conversation, such as community meetings or one-on-one interviews, is particularly helpful.

Consider community context

Health educators emphasize the need to consider study participants' sociodemographic characteristics, including language, education level, age, ethnicity, and gender. In addition, it is important to take into account the study community's unique social, historical, and environmental setting. Our research suggests that participants' prior experience with illness and environmental pollution may shape their interpretation of and response to personal exposure study results (Adams 2011; Altman 2008). For example, participants in our Household Exposure Study initially associated pollution with local sources, including a military reservation, a refinery, and other industry, and with well-known contamination events outside the community such as Love Canal. This focus on outdoor pollution sources led many participants to overlook sources from everyday consumer products and activities. In another example of how history affects report-back, in Richmond, CA — a community bordering an oil refinery, other industry, and transportation corridors — the active organizing and policy campaigns

of study partner Communities for a Better Environment gave study participants a better baseline understanding of how community-level action can address exposures. Our findings underscore that there is no one-size-fits-all model for report-back; rather, decisions about reporting results will depend on the particular study and community. Considering community context when deciding how to report results will help ensure report-back is understandable and meaningful.

Pilot test materials

Developing report-back materials is an iterative process. In order to experiment with various designs and modifications to find the right fit, teams can collect informal feedback from community representatives or conduct more formal communications studies or pilot testing to evaluate whether report-back materials are responsive, understandable, appealing, and appropriate.

DISTRIBUTING RESULTS

Options for disseminating results include mail, telephone, drop-off, face-to-face, internet, or a combination of approaches. Consulting with community members will help teams determine a suitable process. In our experience combining communication materials with in-person home visits works well; however, this is not always feasible.

Our Northern California Household Exposure Study used a combination of mail, drop-off, telephone, and face-to-face communication for reporting results. A team

"SO THAT'S WHAT I THINK BIOMONITORING IS GOOD FOR…PERSONAL CHOICES, POLITICAL ACTION, PUBLIC AWARENESS…BUT ALSO THE CREATION OF COMMUNITY. WHEN YOU'VE GOT A COMMUNITY OF PEOPLE THAT TRUST EACH OTHER AND HAVE MOVED THROUGH THE GRIEF, THEN YOU CAN START MAKING CHANGES."

FIGURE 12.
RECOMMENDATION FOR FURTHER RESEARCH OR ACTION SHOULD BE CALIBRATED TO REFLECT THE LEVEL OF CERTAINTY IN KNOWLEDGE ABOUT HEALTH EFFECTS AND EXPOSURE REDUCTION METHODS (BRODY 2007).

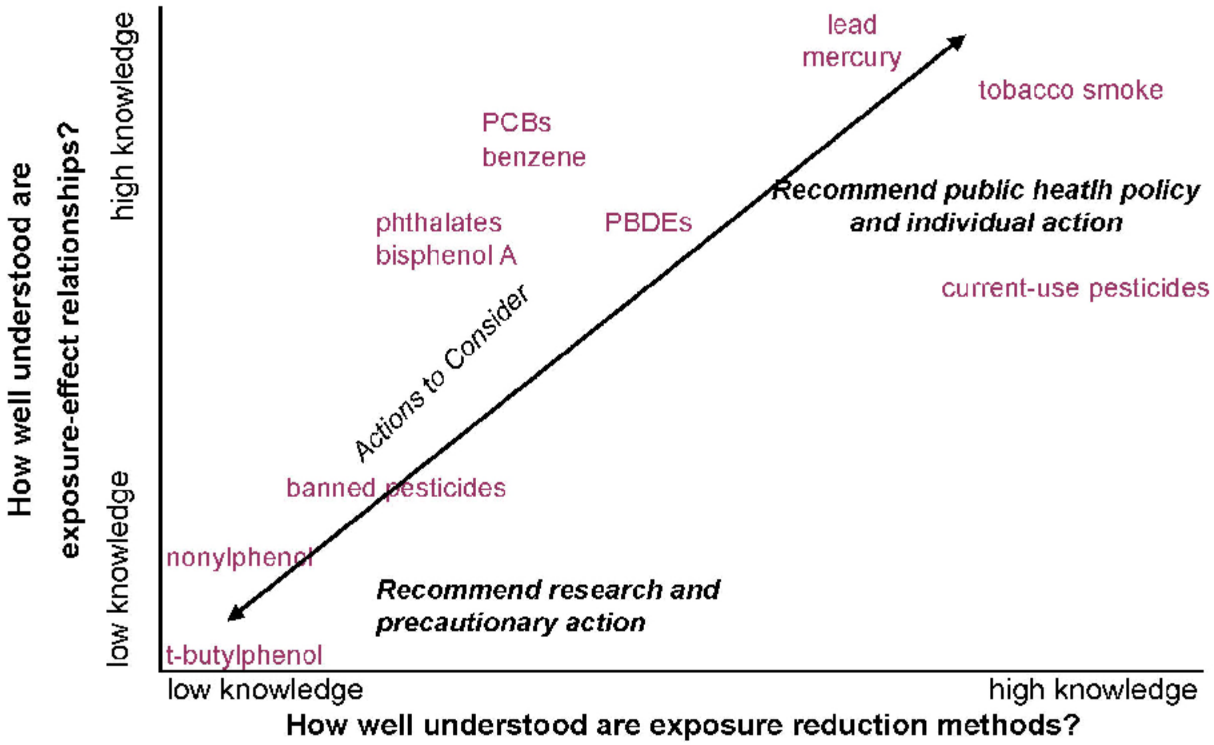

member first called participants who had requested their results during informed consent to let them know they would receive a packet of their results in the mail and to invite them to schedule a home visit to review the results. Results packets were then mailed or hand delivered. During home visits, team members reviewed the results packets, answered questions, and provided information about exposure reduction strategies (**Appendix B**).

Our interviews with participants reveal that they appreciated the opportunity for the face-to-face conversations (Adams 2011). One participant commented:

> ...just very supportive comments and good insights in terms of how my place registered in relation to the others that have been tested. And the sense, I got the sense that there were things that I could do and that helped.

COMMUNITY REPORT-BACK

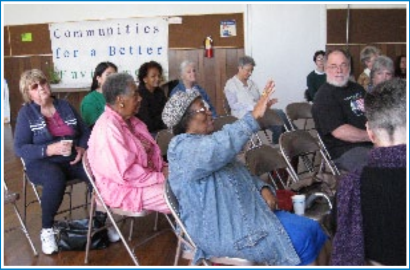

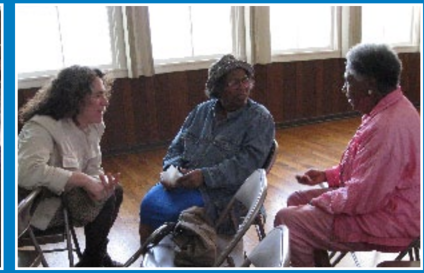

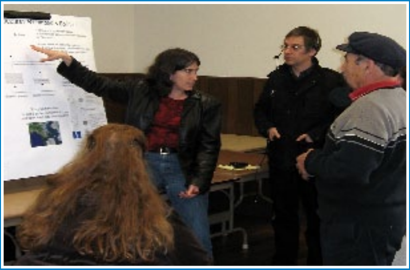

Research teams that develop report-back protocols need to carefully consider the relationship between reporting results to individuals and reporting aggregate results to the study community, the broader scientific community, and the news media. Based on our experience, we suggest the following guidelines for community level report-back:

- ***Host community meetings***. This can provide opportunities to present aggregate study findings to community residents, study participants, public officials and other stakeholders; answer questions about the study; provide information about exposure reduction strategies; obtain feedback about the study and report-back process; facilitate discussion about study findings; and provide an opportunity for participants to brainstorm ways to act on study findings. If there is a community advisory board (which we generally believe to be integral), advisers should be encouraged to attend and co-host the meeting.
- ***Provide brief fact sheets***. Include information about why and how the study was done, the results, and their meaning and implications for action at both the individual and community or national level. Remind participants what information the study can and cannot provide. Those attending community meetings can take this fact sheet to others to further disseminate findings.
- ***Publish study findings***. Publishing study findings in scientific journals is a no-brainer for researchers, but we also consider it a CBPR responsibility in many studies. It supports translating study findings into policy. It also aids community outreach, because peer-reviewed publication signals to journalists that the work is scientifically sound, encouraging them to cover the story. We encourage researchers to publish their report-back methods and evaluations, too.
- ***Be proactive with media***. Media coverage of study findings becomes part of the report-back experience for participants. CBPR scientists need to coordinate strategies, roles, and messages with community partners. Developing media talking points before community meetings and issuing press releases can provide teams with greater control over messaging, improving the accuracy of the reporting. Working with the news media can be a highly cost-effective strategy for reaching study participants, community members, and decision-makers. Don't forget to include Spanish-language press or others relevant to specific studies.
- ***Incorporate fact sheets and action links on web sites and in social media***. Online communications may reach a different audience and links to community organizations or national policy action can help people take action on what they have learned.

A comment from another participant reflects that the report-back conversations built trust:

> I felt good someone was recording these things and that maybe there would be some results or some, you know, future improvement because of the study. And that somehow industry might not be able to get away with as much as they do get away with. There would be more awareness. So it was good, I felt good about that. That we weren't just being ignored.

Such resource intensive face-to-face meetings may not be possible for larger scale studies. In these cases, mailed materials combined with a clear way to contact a research team member with follow-up questions or an online tutorial or video could serve a similar function. Regardless of which strategy a team chooses, we expect a researcher would still need to be accessible by phone to respond to questions. Based on our experience, the research team can expect a small number of participant queries. In addition, researchers may want to phone participants with unusually high measurements. We used this strategy for homes in our Cape Cod Household Exposure Study where we found exceptionally high levels of contaminants in about a dozen homes (Rudel 2008). This additional contact enabled us to discover the source of high PCBs.

Active versus passive reporting

While our study used an active form of report-back in which participants were asked directly if they wished to receive individual results, some teams have used a passive, or "opt-in," form, where participants must contact the study team to request their results. Although both methods can provide participants with the option to not receive their results, passive reporting makes it harder for participants who do want them. Experience shows that participants who wish to receive results don't always contact the research team to get them. One researcher we interviewed noted that a third of participants who did not initially call for results later expressed interest in getting their results during a follow-up survey a week later (Morello-Frosch 2009). A recent assessment by Wu et al.(2009) found that the passive reporting techniques used in a breast milk biomonitoring study created a barrier to accessing results. In that study, only 30% of participants contacted researchers for their results after receiving a letter letting them know there results were available. In contrast, nearly all participants elected to receive their results in our active-report-back study. We believe asking participants directly whether they want their results is more respectful than placing the burden on them to ask.

Timing

Reporting individual results should be part of a strategy for reporting aggregate results to the study community, the broader scientific community, and the news media (See Box on community report-back). The sequence of these events depends on weighing a number of factors: participant and community wishes, time required for data analysis, scientific journal publication practices, and media coverage of results presented at scientific conferences. Study teams have a responsibility to return results promptly; however, reporting results too soon could mean the interpretation doesn't benefit from the peer review process. Also, peer-reviewed publication is a signal of credibility to the news media, which affects the coverage and impact of the results.

However teams decide to proceed, they should communicate with participants up front about expected timing and update them if the schedule changes.

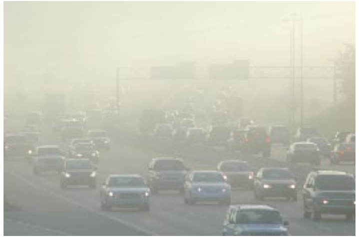

People know that auto exhaust is pollution, but learning personal results connects this information to health in a new way.

Community first report-back

The community advisory council for a study of PFOA in residents of Little Hocking, Ohio, developed a "community first" communication model that carefully timed sequential communications (Emmett 2009). After peer-review but before publication, results were reported to participants, local medical providers, and relevant authorities, followed by the news media and the local community. Researchers may find that some communities, especially tribal ones with their own IRBs and similar bodies, have express criteria for "community-first" report-back.

Develop a response plan

Learning about contamination in one's own home or body can be worrisome, and research teams should be prepared to respond to concerns. Some researchers have worried that reporting individual results when the health effects of target chemicals are uncertain could particularly cause undue distress. The experiences in our Household Exposure Study and other studies described in this handbook have been reassuring. In the Household Exposure Study, most participants had a moderate response to receiving their results. Some were initially surprised or puzzled by results but not unduly worried. We found that making team members available to answer questions and provide recommendations for action helped participants process the information and mitigate distress.

Teams can be proactive by developing a plan and delineating responsibilities for how they will respond to participants' questions and concerns, for example about the health implications of the findings or opportunities for exposure reduction. Resources should include both a senior researcher, who knows the underlying science, and someone who is connected with and skilled at talking with the study participants' community, whether community is geographically based or defined by shared illness or cultural experiences.

Our team and colleagues who have reported individual exposure results have found this to be a valuable experience for both researchers and participants. Research about report-back documents that these methods can be effective. We are eager to hear about your experiences, too.

"…I REALLY THOUGHT I WAS LILY WHITE AND PURE. I MEAN, EVEN AFTER WE LIVED FOUR YEARS IN [BIG EASTERN CITY], RIGHT DOWNTOWN, I KNEW I HAD SOME CARBON MONOXIDE, OR, I MEAN, SOMETHING FROM CARS OR YOU KNOW, SOMETHING LIKE THAT, BUT ALL OF A SUDDEN I READ, "WE DETECTED 19 CHEMICALS IN YOUR DAUGHTER'S URINE" AND I'M LIKE…'CHEMICALS!' I HAVE RESIDUES FROM INSECTICIDES AND DISINFECTANTS AND WOOD PRESERVATIVES AND MOTHBALLS, AND UM…IT JUST SHOCKED ME BECAUSE IT HIT HOME TOO MUCH. I NEVER, NEVER SAW IT COMING FOR ME. I ALWAYS HAVE LIVED A HEALTHY LIFE, LIFESTYLE. AND THIS JUST SHOCKED ME…THEN, WHEN I REALIZED IT'S FROM FRAGRANCES AND SOAPS AND DETERGENTS AND THINGS…"

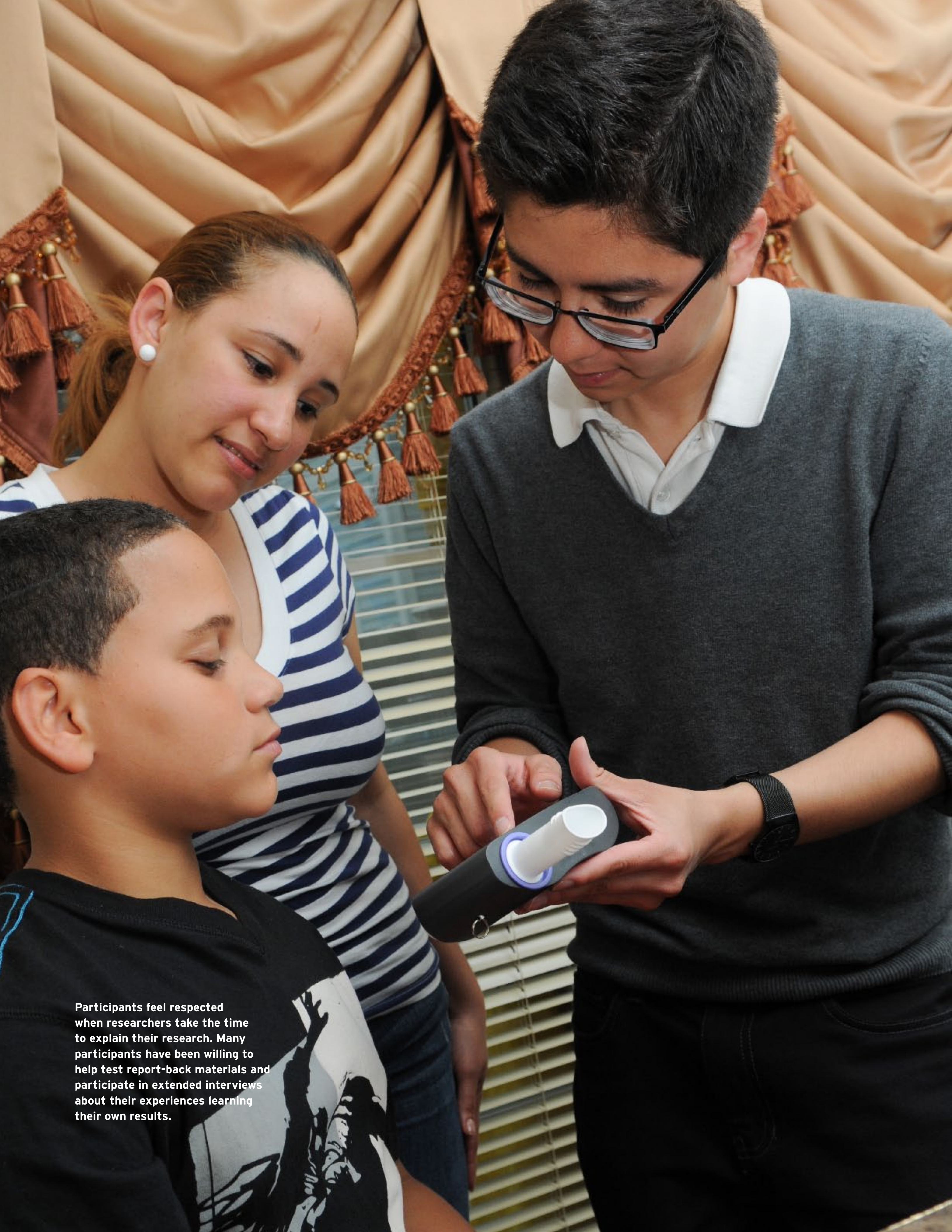

Participants feel respected when researchers take the time to explain their research. Many participants have been willing to help test report-back materials and participate in extended interviews about their experiences learning their own results.

CHAPTER 3: EVALUATING REPORT-BACK

> "THAT'S THE KEY OF WHAT WE'RE TALKING ABOUT. IT SHOULD BE YOUR CHOICE TO GET THE INFO THAT YOU WANT OR DON'T WANT."

WHY EVALUATE THE REPORT-BACK PROCESS?

A growing number of researchers are reporting individual results to participants in biomonitoring and personal exposure studies and some have evaluated the process (Adams 2011; Altman 2008; Brody 2007; Morello-Frosch 2009; Quandt 2004; Wu 2009). This is a new field, though, so collecting additional information about participants' experiences, including how they perceive and act on their results, can help improve ethical and effective approaches. Methods for evaluating report-back include interviews with participants after they receive results, surveys conducted at community meetings, and focus groups.

PLANNING AHEAD

Developing an evaluation plan early in the report-back process can help teams allocate adequate time and resources, avoid IRB roadblocks, and facilitate interviews with participants soon after they receive results. Keep in mind that evaluation materials used with participants need to be approved by the study's IRB. It may be helpful to partner with a social scientist who has experience with qualitative techniques to develop and implement the report-back evaluation.

INTERVIEWING PARTICIPANTS ABOUT THEIR EXPERIENCES

Interviews with participants can provide rich information about their experiences with receiving their own results and the effectiveness of report-back practices. The planning steps include developing an interview schedule, getting informed consent, selecting interviewers and interviewees, conducting interviews, and interpreting participant responses.

Developing an interview protocol

Interviews with participants can provide rich information about their experiences with receiving their own results and the effectiveness of report-back practices. The planning steps include developing an interview schedule, getting informed consent, selecting interviewers and interviewees, conducting interviews, and interpreting participant responses.

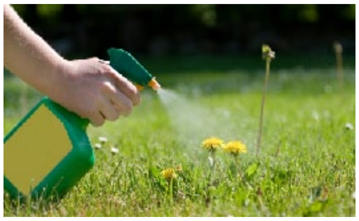

In the Household Exposure Study, participants were surprised to learn that pesticides used on weeds outdoors were tracked inside.

In our Household Exposure Study, we used a semi-structured interview organized around topics such as participation in sample collection, interpreting and understanding results, and general implications of the study (**Appendix E**). For example, our interviews begin with questions that engage participants and are easy to answer, and then the section about interpreting results asks the following question and series of follow-up questions:

4. Do you recall what the study found in your home? [Long pause, wait for answer]

   a. Do you remember any specific chemicals that were found in your home?
   b. Would you say that the levels of any chemicals found in your home were "high"?
      i. Do you think the results seem high compared to what was found in other people's homes? Which chemicals were you thinking about? What makes you think that?
      ii. Do you think the results seem high compared to government guidelines? Which chemicals were you thinking about? What makes you think that?
   c. Were you surprised by the results? *If so, probe:*
      – What was surprising?
   d. Are you concerned about any possible health implications for you and your family from the chemicals found in your home?
   e. Did you find the conversation with the researcher useful? *If so, probe:*
   i. What was useful about that?

This example illustrates some additional features of interview schedules including the use of open-ended questions, which encourage descriptive answers, and probes, which allow interviewers to follow-up for clarification or additional information.

As with report-back materials, when developing interview schedules, teams should consider socio-demographic and community context. Interview protocols may need to be translated and conducted in multiple languages. Include terminology that is consistent with other study materials such as the report-back packets and study fact sheets. We go through many iterations in order to best cover a lot of material without too many questions, and with a combination of the most precise wording and the most openness to elicit detailed responses.

Getting informed consent, again

Teams that decide to interview participants will need to obtain informed consent for the interviews. We first discussed follow-up interviews with participants in our Household Exposure Study during informed consent for sample collection and included this text in the informed consent form (**Appendix E**):

> If you choose to receive your results from this study, we will visit you a third time after you receive the results to interview you about your experiences participating in the study and receiving your results. The third visit will last approximately one hour.

When we met with participants to report their individual study results, we asked for permission to re-contact them to ask about their experience. After we reported results, we phoned participants to schedule visits to their homes to conduct the follow-up interviews. During the follow-up visits we obtained informed consent for the interviews. For example, our informed consent form (**Appendix E**) includes the following text:

> Now, if you agree, we would like to interview you about your response to how we reported to you what chemicals we found in your home. The interview will take about an hour. The questions are designed to help us evaluate how well we did in informing study participants about what was found in their homes and also to find out what people think was important about their results. With your permission, we will tape record the interview to ensure that we get the most complete record of your responses.

Selecting interviewers

Interviews can be conducted by trained researchers or community members who are familiar with study findings and how to interpret them, but may be blinded to individual participants' results. They should be prepared to answer questions about how to read the graphs, sources of target chemicals, and exposure reduction strategies. For example, interviewers for our Household Exposure Study found it useful to bring a packet of handouts covering relevant exposure reduction strategies such as IPM.

Conducting interviews

Conducting interviews in-person can create a rich interaction that allows interviewers to pick up on non-verbal cues and build rapport. Conducting interviews over the phone is another option. In an evaluation of a breast milk biomonitoring study, Wu et al.(2009) conducted brief telephone interviews to evaluate the report-back process and assess how participating in the study and receiving results affected participants' attitudes towards breastfeeding.

Teams may wish to interview only a subset of participants, particularly in studies with larger study populations. Based on our experience in the Household Exposure Study and on experience in mental models studies (Morgan 2002), approximately 20 - 25 interviews is enough to provide saturation; that is, the point at which responses largely duplicate prior participants' answers. A larger number of interviews would be needed to evaluate how common certain types of responses are, especially if there were multiple racial-ethnic and income groups.

When developing and conducting interviews, it is important to keep in mind that the interviews are not meant to "test" participants, but rather to elicit participants' experiences with and views on report-back. Focusing on participants' experiences rather than on what details they can recall will generate more helpful responses. We avoid "test-like" questions that make the interview seem like an evaluation of the participant rather than an inquiry about the report-back process.

Case Study: Household Exposure Study

In our Household Exposure Study, we evaluated our report-back process by conducting hour-long in-person interviews with 57 study participants (Adams 2011; Altman 2008). Interviews were designed to assess:

- How participants interpreted the materials
- What information helps participants understand and contextualize results
- Participants' emotional responses to receiving results
- What practices are appropriate in particular community contexts
- What actions participants considered or took in response to receiving their results

Interviews revealed that participants from diverse socioeconomic and educational backgrounds were able to grapple with complex results and uncertain health implications. Participants learned about everyday exposures to environmental chemicals, demonstrating how report-back can increase environmental health literacy. We found that participants understood key messages from the study findings. Participants often expressed surprise at their results but not undue worry or stress. For example,

"AND THAT'S WHAT I WOULD WANT FROM THIS [EXPOSURE] STUDY, IS GIVE ME SOMETHING I CAN DO ABOUT IT. DON'T JUST GIVE ME INFORMATION THAT TELLS ME I HAVE PROBLEMS. ... BECAUSE THAT'S FRUSTRATING, YOU KNOW? BUT I'M PROACTIVE ENOUGH THAT I'LL SAY, "OK, I HAVE THIS INFORMATION NOW IT'S UP TO ME TO DO SOMETHING."

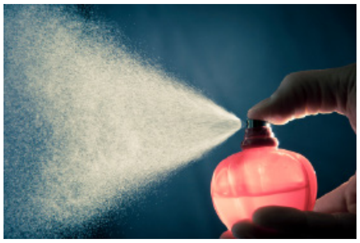

Chemicals from consumer products linger in household air and dust, exposing everyone in the home.

participants were surprised to learn about indoor pollution from everyday products and activities:

"It's interesting how the indoor [pollution] is higher in both of the communities… I mean they're very starkly different. Polar opposite-type of environments."

Interviews revealed that reporting individual results motivated individual and collective action to reduce exposures.

Case Study: La Familia Study

In the La Familia Study, Quandt et al (2004) conducted in-person interviews to assess participants' reaction to receiving information about pesticides detected in their homes, how participants interpreted the report-back materials, and how well the study's main risk communication messages were conveyed. Interviews revealed that participants were able to comprehend the main points of the risk communication and to interpret the figures presented. The findings indicate that even participants with low literacy were able to understand complex scientific concepts. Participants' reactions to results varied, including relief, surprise, concern, and complacency.

Case Study: Greater Boston PBDE Breastmilk Biomonitoring Study

In the Greater Boston PBDE Breastmilk Biomonitoring Study, Wu et al (2009) conducted brief telephone interviews to assess how participating in the biomonitoring study and receiving results affected participants' attitudes towards breastfeeding and to evaluate the report-back process. Findings suggest that receiving individual results and other study materials did not negatively impact breastfeeding behavior, and that the context and manner in which the results were reported mitigated any potentially negative impacts. Participants' responses indicate that the study provided an opportunity for learning about environmental health and motivated some participants to reduce exposures or engage in other preventive behaviors.

Case Study: The Growing Up Female Study

Parents of participants in the Growing up Female Study, an epidemiological study of young girls in Ohio, reported a positive experience receiving individual test results of blood levels of PFOA (Hernick 2011). One participant commented, "Let me get this straight: You have found something, you do not know the cause or solution? Thank you for doing the right thing morally and ethically for sharing this information with us" (Hernick 2007). Other benefits of reporting individual results noted by the study team include providing parents with a better understanding of the relevance of the study, an opportunity to dialogue with study families, and better study retention.

EVALUATING COMMUNITY MEETINGS WITH SURVEYS

In the Household Exposure Study, aggregate results were reported at community meetings held in the study communities. To evaluate this part of the report-back process, we distributed anonymous five-question surveys at the community meetings that asked attendees why they attended, what they hoped to learn, and what follow-up questions they may have. We learned that people felt they learned a lot from the presentations, were grateful for the information we presented, and felt they could use the information to improve their community (**Appendix F**).

“LET ME GET THIS STRAIGHT: YOU HAVE FOUND SOMETHING, YOU DO NOT KNOW THE CAUSE OR SOLUTION? THANK YOU FOR DOING THE RIGHT THING MORALLY AND ETHICALLY FOR SHARING THIS INFORMATION WITH US.”

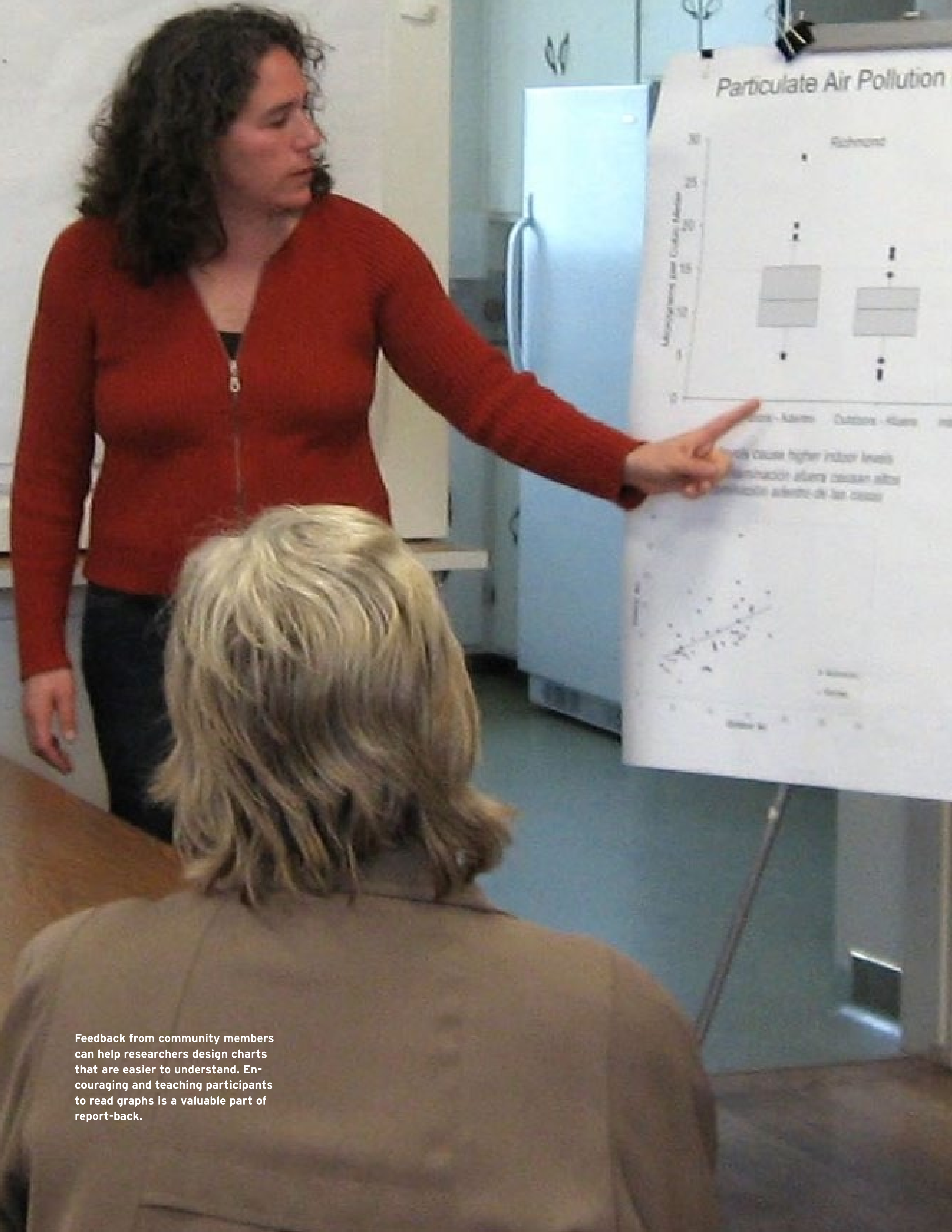

Feedback from community members can help researchers design charts that are easier to understand. Encouraging and teaching participants to read graphs is a valuable part of report-back.

CONCLUSION

Active report-back of personal exposure data to participants is an exciting area that combines good environmental health science, democratic ethics, and community empowerment. This approach offers the potential for high quality, innovative science that at the same time benefits individuals and communities who participate. As a fairly recent approach, report-back has begun to have important impacts, especially regarding emerging contaminants about which little has been previously known. As with any new approach, practitioners, participants, and IRBs often lack guidance on how to do this work. This handbook provides that guidance, as well as offering scientific support for this approach.

Researchers, government agencies, and community groups engaged in biomonitoring — and other personal exposure studies, such as household sampling, as well — can benefit from extensive communication with each other about their experiences and their hopes for expanding and improving such work in the future. We seek communication from others engaged in report-back, so that we can update this handbook and serve as a resource for others involved in similar work.

We welcome your input. Please send comments about this handbook and examples of your own approaches to us at info@silentspring.org.

"I APPRECIATE THAT YOU AND YOUR COLLEAGUES DO THIS WORK. YOU KNOW, IT'S LIKE WE'RE NOT BEING IGNORED."

REFERENCES

Adams C, Brown P, Morello-Frosch R, Brody J, Rudel R, Zota A, Dunagan S, Tovar J, Patton S. 2011. Disentangling the Exposure Experience: The Roles of Community Context and Report-back of Environmental Exposure Data. Journal of Health and Social Behavior 52(2): 180-196.

Altman R, Morello-Frosch R, Brody J, Rudel R, Brown P, Averick M. 2008. Pollution Comes Home and Gets Personal: Women's Experience of Household Toxic Exposure. Journal of Health and Social Behavior 49(4): 417-435.

Bradman A. 2007. Reporting Biomonitoring Results to Individuals and Medical and Public Audiences: Challenges and Opportunities. Berkeley: University of California, Berkeley, Center for Children's Environmental Health Research.

Brody JG, Morello-Frosch R, Brown P, Rudel RA, Altman RG, Frye M, Osimo CA, Perez C, Seryak LM. 2007. Improving Disclosure and Consent: "Is It Safe?": New Ethics for Reporting Personal Exposures to Environmental Chemicals. American Journal of Public Health 97(9): 1547-1554.

Brody JG, Morello-Frosch R, Zota A, Brown P, Perez C, Rudel RA. 2009. Linking Exposure Assessment Science with Policy Objectives for Environmental Justice and Breast Cancer Advocacy: The Northern California Household Exposure Study. American Journal of Public Health 99 Suppl 3: S600-609.

Brown-Williams H. 2009a. Integrating Health Literacy Best Practices in Biomonitoring Results Communication. In: Proceedings of the The California Environmental Contaminant Biomonitoring Program (CECBP) Scientific Guidance Panel, July 28-29, 2009, Oakland, CA.

Brown-Williams H, Graham C, Oliver M. 2009b. Report on CYGNET Study Focus Groups: Communicating Results to Study Participants. Berkeley: Health Research for Action, University of California, Berkeley School of Public Health.

Brown P, Morello-Frosch R, Brody JG, Altman RG, Rudel RA, Senier L, Perez C, Simpson R. 2010. Institutional Review Board Challenges Related to Community-Based Participatory Research on Human Exposure to Environmental Toxins: A Case Study. Environmental Health 9: 39.

Commonweal Biomonitoring Resource Center and the Body Burden Work Group. 2007. Is It In Us? Chemical Contamination in Our Bodies. Available: http://www.isitinus.org/documents/Is%20It%20In%20Us%20Report.pdf [accessed: 1 July 2013].

Dodson RE, Perovich LJ, Covaci A,Van den Eede N, Ionas AC, Dirtu AC, Brody JG, Rudel R. 2012. After the PBDE Phase-out: A Broad Suite of Flame Retardants in Repeat House Dust Samples from California. Environmental Science & Technology 46(24): 13056-13066.

Emmett EA, Zhang H, Shofer FS, Rodway N, Desai C, Freeman D, Hufford M. 2009. Development and Successful Application of a "Community-First" Communication Model for Community-Based Environmental Health Research. Journal of Occupational and Environmental Medicine, American College of Occupational and Environmental Medicine 51(2): 146-156.

Few S. 2004. Show Me the Numbers: Designing Tables and Graphs to Enlighten. Oakland, CA: Analytics Press.

Hernick AD. Talking with Our Study Families: What, When and How to Report Study Findings. In: Proceedings of the 4th Annual Early Environmental Exposures Conference, 2007. Cincinatti, OH: Breast Cancer & the Environment Research Centers (BCERC).

Hernick AD, Brown MK, Pinney SM, Biro FM, Ball KM, Bornschein RL. 2011. Sharing Unexpected Biomarker Results with Study Participants. Environmental Health Perspectives 119(1): 1-5.

Judd S, Plumb M. 2012. Reporting Personal Exposures to Environmental Chemicals: Focus Group Feedback. Oakland: Child Health and Development Studies, Public Health Institute.

Morello-Frosch R, Brody JG, Brown P, Altman RG, Rudel RA, Perez C. 2009. Toxic Ignorance and the Right-to-Know: Ethical and Scientific Dilemmas of Reporting Data in Body Burden Research. Environmental Health 8(6).

Morello-Frosch R, Pastor M, Sadd J, Porras C, Prichard M. 2005. Citizens, Science, and Data Judo: Leveraging Community-based Participatory Research to Build a Regional Collaborative for Environmental Justice in Southern California. In: Methods for Conducting Community-Based Participatory Research in Public Health, (Barbara Israel EE, Amy Shultz, Edith Parker, eds). San Francisco: Jossey-Bass Press.

Morgan MG, Fischhoff B, Bostrom A, Atman CJ. 2002. Risk Communication: A Mental Models Approach. Cambridge, UK: Cambridge University Press.

National Academy of Sciences. 2006. Human Biomonitoring for Environmental Chemicals. Available: http://www.nap.edu/catalog/11700.html [accessed 1 July 2013].

National Institutes of Health. 2009. Certificates of Confidentiality Kiosk. Available: http://grants.nih.gov/grants/policy/coc [accessed 17 June 2013].

Nelson JW, Scammell MK, Altman RG, Webster TF, Ozonoff DM. 2009. A New Spin on Research Translation: The Boston Consensus Conference on Human Biomonitoring. Environmental Health Perspectives 117(4): 495-499.

Pausentbach D, Galbraith D. 2006. Biomonitoring: Is Body Burden Relevent to Public Health? Regulatory Toxicology and Pharmacology 44: 249-261.

Penslar RL. 1993. Institutional Review Board Guidebook. The Poynter Center for the Study of Ethics and American Institutions, Indiana University. Available: http://www.hhs.gov/ohrp/archive/irb/irb_guidebook.htm [accessed 5 July 2013].

Perata. 2006. The California Environmental Contaminant Biomonitoring Program. In: Senate Bill No 1379 (California Department of Public Health, the Office of Environmental Health Hazard Assessment, and the Department of Toxic Substances Control, ed), 1-7.

Quandt SA, Doran AM, Rao P, Hoppin JA, Snively BM, Arcury TA. 2004. Reporting Pesticide Assessment Results to Farmworker Families: Development, Implementation, and Evaluation of a Risk Communication Strategy. Environmental Health Perspectives 112(5): 636-642.

Rudel RA, Camann DE, Spengler JD, Korn LR, Brody JG. 2003. Phthalates, Alkylphenols, Pesticides, Polybrominated Diphenyl Ethers, and Other Endocrine-Disrupting Compounds in Indoor Air and Dust. Environmental Science and Technology 37(20): 4543-4553.

Rudel RA, Dodson RE, Perovich LJ, Morello-Frosch R, Camann DE, Zuniga MM, Yau AY, Just AC, Brody JG. 2010. Semivolatile Endocrine Disrupting Compounds in Paired Indoor and Outdoor Air in Two Northern California Communities. Environmental Science and Technology 44(17): 6583-6590.

Rudel RA, Seryak LM, Brody JG. 2008. PCB-Containing Wood Floor Finish is a Likely Source of Elevated PCBs in Residents' Blood, Household Air and Dust: A Case Study of Exposure. Environmental Health 7(1): 2.

US Department of Health and Human Services. 1991. Federal Policy for the Protection of Human Subjects ('Common Rule'). 45 C.F.R. 46.

US Environmental Protection Agency. 1988. Seven Cardinal Rules of Risk Communication. Available: http://www.epa.gov/CARE/library/7_cardinal_rules.pdf [accessed 5 July 2013].

Wu N, McClean MD, Brown P, Aschengrau A, Webster TF. 2009. Participant Experiences In a Breastmilk Biomonitoring Study: A Qualitative Assessment. Environmental Health 8(4).

RESOURCES

The Belmont report
http://www.hhs.gov/ohrp/humansubjects/guidance/belmont.html

45 CFR Part 46
http://www.hhs.gov/ohrp/humansubjects/guidance/45cfr46.html

Certificate of confidentiality kiosk
http://grants.nih.gov/grants/policy/coc/

A guide for choosing and designing health-related materials
http://www.nigz.nl/inc/pdf.cfm?filename=instrumenten/healthlitbrochure.pdf

Guidance on Written IRB Procedures
http://www.hhs.gov/ohrp/policy/irbgd107.html

Partnerships in environmental public health (PEPH) materials
http://www.niehs.nih.gov/research/supported/programs/peph/materials/index.cfm

Seven cardinal rules of risk communication
http://www.epa.gov/CARE/library/7_cardinal_rules.pdf

OUR ARTICLES ON REPORT-BACK

1) "Improving Disclosure and Consent: 'Is It Safe?' New Ethics for Reporting Personal Exposures to Environmental Chemicals" (Julia Green Brody, Rachel Morello-Frosch, Phil Brown, Ruthann A. Rudel, Rebecca Gasior Altman, Margaret Frye, Cheryl C. Osimo, Carla Perez, and Liesel M. Seryak). *American Journal of Public Health*, 2007 97: 1547-1554.

2) "Experts, Ethics, and Environmental Justice: Communicating and Contesting Results from Personal Exposure Science" (Rachel Morello-Frosch, Phil Brown, Julia Green Brody, Rebecca Gasior Altman, Ruthann A. Rudel, Ami Zota, and Carla Perez). In Gwen Ottinger and Benjamin Cohen, eds., *Engineers, Scientists, and Environmental Justice*. Cambridge, MA: MIT Press 2011.

3) "Pollution Comes Home and Gets Personal: Women's Experience of Household Chemical Exposure." (Rebecca Gasior Altman, Rachel Morello-Frosch, Julia Green Brody, Ruthann Rudel, Phil Brown, and Mara Averick) *Journal of Health and Social Behavior* 2008 49:417-

4) "'Toxic Ignorance' and the Right-to-Know: Assessing Strategies for Biomonitoring Results Communication in a Survey of Scientists and Study Participants" (Rachel Morello-Frosch, Julia Green Brody, Phil Brown, Rebecca Gasior Altman, Ruthann A. Rudel, Carla Pérez. *Environmental Health*. 2009 8:6.

5) "Linking Exposure Assessment Science with Policy Objectives for Environmental Justice and Breast Cancer Advocacy: The Northern California Household Exposure Study" (Julia Green Brody, Rachel Morello-Frosch, Ami Zota, Phil Brown, Carla Pérez, and Ruthann A. Rudel). *American Journal of Public Health* 2009 99:S600-S609

6) "Institutional Review Board Challenges Related to Community-Based Participatory Research on Human Exposure to Environmental Toxins: A Case Study" (Phil Brown, Rachel Morello-Frosch, Julia Green Brody, Rebecca Gasior Altman, Ruthann A. Rudel, Laura Senier, Carla Pérez and Ruth Simpson). *Environmental Health* 2010 9:39.

7) "Disentangling the Exposure Experience: The Roles of Community Context and Report-back of Environmental Exposure Data" (Crystal Adams, Phil Brown, Rachel Morello-Frosch, Julia Green Brody, Ruthann Rudel, Ami Zota, Sarah Dunagan, Jessica Tovar, and Sharyle Patton). *Journal of Health and Social Behavior* 2011 52 (2):180-196.

8) "Measuring The Success Of Community Science: The Northern California Household Exposure Study" (Phil Brown, Julia Green Brody, Rachel Morello-Frosch, Jessica Tovar, Ami R. Zota, and Ruthann A. Rudel). *Environmental Health Perspectives* 2012, 120:326–331.

APPENDIX

A. CALIFORNIA HOUSEHOLD EXPOSURE STUDY SAMPLE REPORT-BACK PACKET

B. CHEMICALS IN OUR BODIES STUDY SAMPLE REPORT-BACK PACKET

C. GREEN CLEANING FACT SHEET

D. TAKE ACTION FACT SHEET

E. CALIFORNIA HOUSEHOLD EXPOSURE STUDY INTERVIEW

F. COMMUNITY MEETING SURVEY: POST-FORUM QUESTIONNAIRE

APPENDIX

A. CALIFORNIA HOUSEHOLD EXPOSURE STUDY SAMPLE REPORT-BACK PACKET

Silent Spring Institute
Brown University
Communities for a Better Environment

617.332.4288 www.silentspring.org
www.brown.edu
510.302.0430 www.cbecal.org

Name
Address
City, State, Zip

December 20, 2010

Dear _____________:

Thank you very much for participating in the Household Exposure Study. When we visited your home to collect air and dust samples for the study last year, you asked us to report back to you on the results; so we are enclosing these results for you.

We tested 50 homes for 185 chemicals. We are enclosing a summary of what was found in your home. The results are shown in more detail in 10 graphs, grouped by chemical families (for example, pesticides) and type of sample (air or dust). For each chemical, the concentration measured in your home is shown in comparison with all 50 homes in the study. The page entitled *A Guide to Reading Your Results* provides more information about how to read your graphs.

We are doing this study because most people spend much of their time at home, so chemicals that people are exposed to at home can be important for health. We are studying chemicals that come from activities inside the home and pollutants that may come from outside. For some chemicals, your results can be compared to a government health guideline. For other chemicals, scientists don't know yet how they affect health, and measuring household levels is the first step.

Some people in the study may want to make changes to reduce the levels of some of the chemicals we found. To help people think about ways to reduce exposure, we have included information about the products, materials, or activities that may be sources of the chemicals in your home or local environment. This information is in your summary and in a detailed table listing each chemical along with a key to the abbreviations used in the graphs.

We know that some people are more comfortable than others reading graphs and tables like the ones included, and we would be glad to talk with you to help you understand your results or answer any questions. Please call Communities for a Better Environment Project Coordinator, Carla Perez, at 510-302-0430 ext. 11 if you have any questions about any parts of the study.

In addition, we will be inviting you to a series of community information sessions over the next two years where we will discuss what we learned from this sampling program. We deeply appreciate your active participation in this project.

Sincerely,
Carla M. Perez, Northern California Program Director
Communities for a Better Environment

Julia G. Brody, Ph.D.
Executive Director
Silent Spring Institute

Summary of Your Results

We tested for 185 chemicals in this study. Overall, we detected many chemicals in every home. Your results are shown in the enclosed graphs.

For your home, we detected**:**
- **27** chemicals in the outdoor air near your home,
- **33** in your indoor air,
- **11** in your dust sample.

Brominated Flame Retardents
Your house contained PBDE flame retardants. The PBDEs in your house dust were generally higher than most others in the study (see page 9, PBDE). PBDE flame retardants are in foam furniture and cushions, and synthetic carpets. These chemicals were banned in Europe because of effects on thyroid hormones.

Pesticides
We found two insecticide ("bug killer") ingredients in your indoor air (page 2) and dust (page 8). You can reduce your exposure by controlling indoor pests with bait traps and other less toxic methods.

Phthalates – Vinyl, Other Plastics, and Cosmetics
Among the 50 homes in our study, you had one of the highest levels of DEHA in air (page 4) and DEP in dust (page 9). You had generally higher levels of phthalates in your outdoor air. We can't tell from this test exactly what the sources of these chemicals are, but the indoor sources could be from cosmetics and products like cologne, a shower curtain, plastic toys, a raincoat, or food packaging. Phthalates were banned from children's toys and cosmetics in Europe, because of concerns about effects on children's development. Phthalates are widely used in the US, and we found phthalates in every house we tested.

Polycyclic Aromatic Hydrocarbons (PAHs)
Some PAHs in your indoor air were higher than others in the study (page 5). PAHs in your home may come from outdoor air sources. Common outdoor sources of PAHs are cars, buses, trucks, and industrial emissions. Common indoor sources are home heating, cooking, and smoking.

Other
A group of chemicals called Alkylphenols are often found in soaps, detergents, and some pesticides. In your indoor air, you had a higher level than most of 1) a chemical found in disinfectants or mothballs (See page 7, 24DCPh), and 2) a chemical found in soaps and detergents (See page 7, NP).

The study team is continuing research to learn how to reduce exposures to pollutants in homes. If you have any questions, please call us at 510-302-0430 ext. 11.

A Guide to Reading Your Results

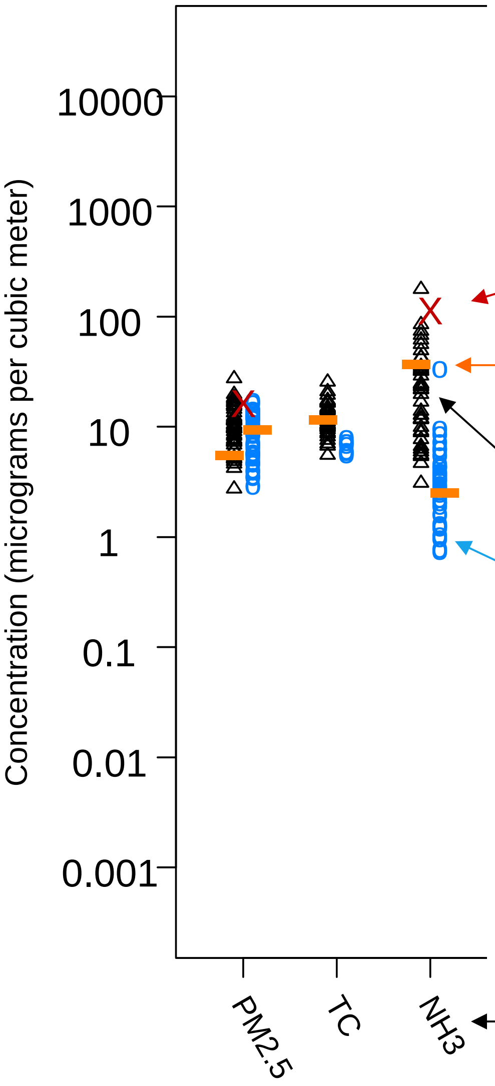

X shows the current EPA health guideline. If your bar is above the X, your results are higher than the guideline.

Your results are marked by orange bars.

If there is no orange bar, then the chemical was not detected in your home.

Each △ represents one other home's indoor air result in the study, and each o represents one other home's outdoor air result.

The column of circles shows the range of concentrations measured.

If your bar is near the top, your result was higher than most; if your bar is near the bottom, your result was lower than most.

You can find more information about each chemical by matching the abbreviation on the graph with the full name on the "Sources" chart.

Pesticides in Air

Participant 47

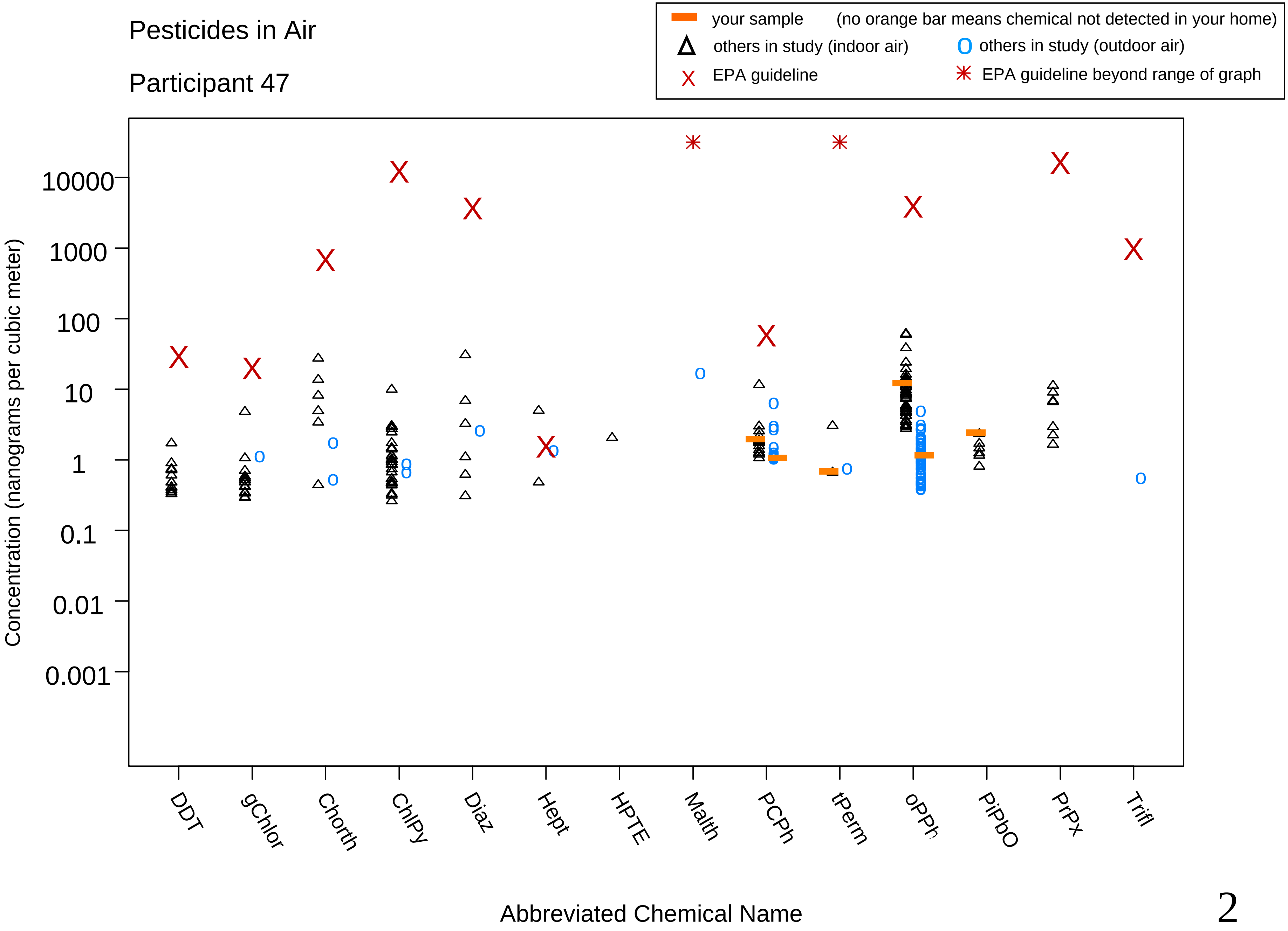

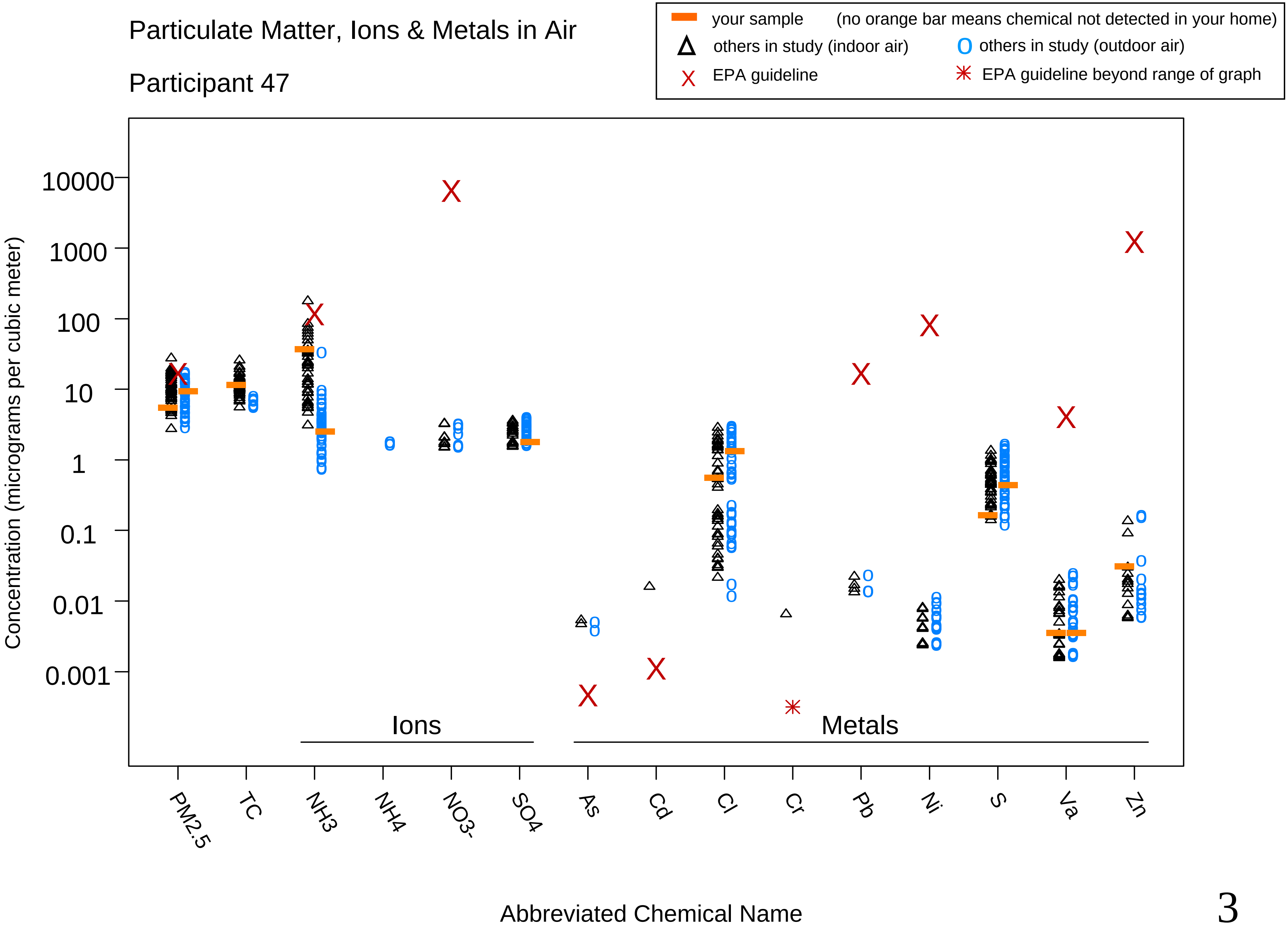

Particulate Matter, Ions & Metals in Air
Participant 47
your sample (no orange bar means chemical not detected in your home)
others in study (indoor air)
others in study (outdoor air)
EPA guideline
EPA guideline beyond range of graph
Concentration (micrograms per cubic meter)
10000
1000
100
10
1
0.1
0.01
0.001
Ions
Metals
PM2.5
TC
NH3
NH4
NO3-
SO4
As
Cd
Cl
Cr
Pb
Ni
S
Va
Zn
Abbreviated Chemical Name

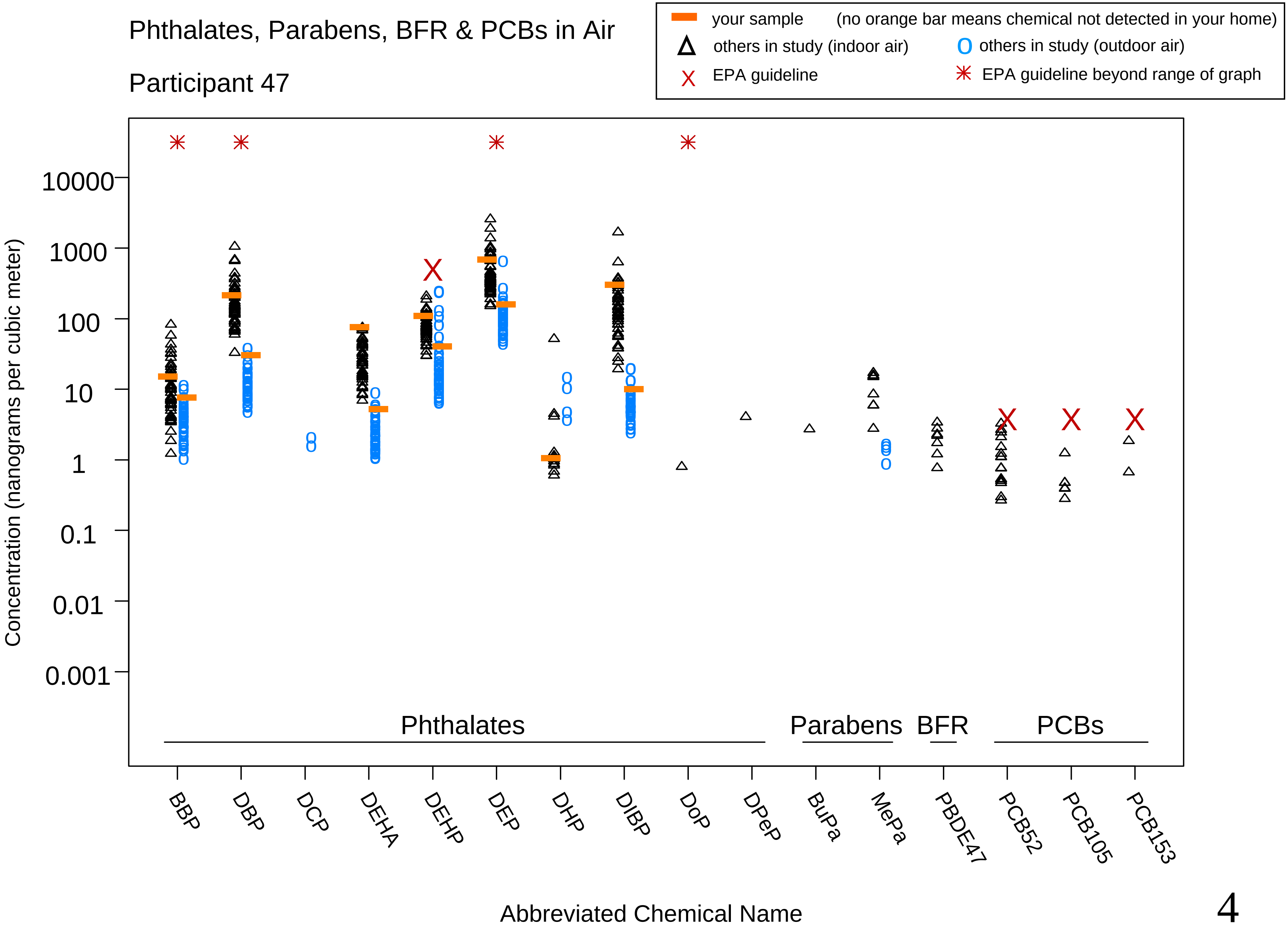

Phthalates, Parabens, BFR & PCBs in Air
Participant 47
your sample
(no orange bar means chemical not detected in your home)
others in study (indoor air)
others in study (outdoor air)
EPA guideline
EPA guideline beyond range of graph
Concentration (nanograms per cubic meter)
10000
1000
100
10
1
0.1
0.01
0.001
Phthalates
Parabens
BFR
PCBs
BBP
DBP
DCP
DEHA
DEHP
DEP
DHP
DIBP
DoP
DPeP
BuPa
MePa
PBDE47
PCB52
PCB105
PCB153
Abbreviated Chemical Name

PAHs in Air

Participant 47

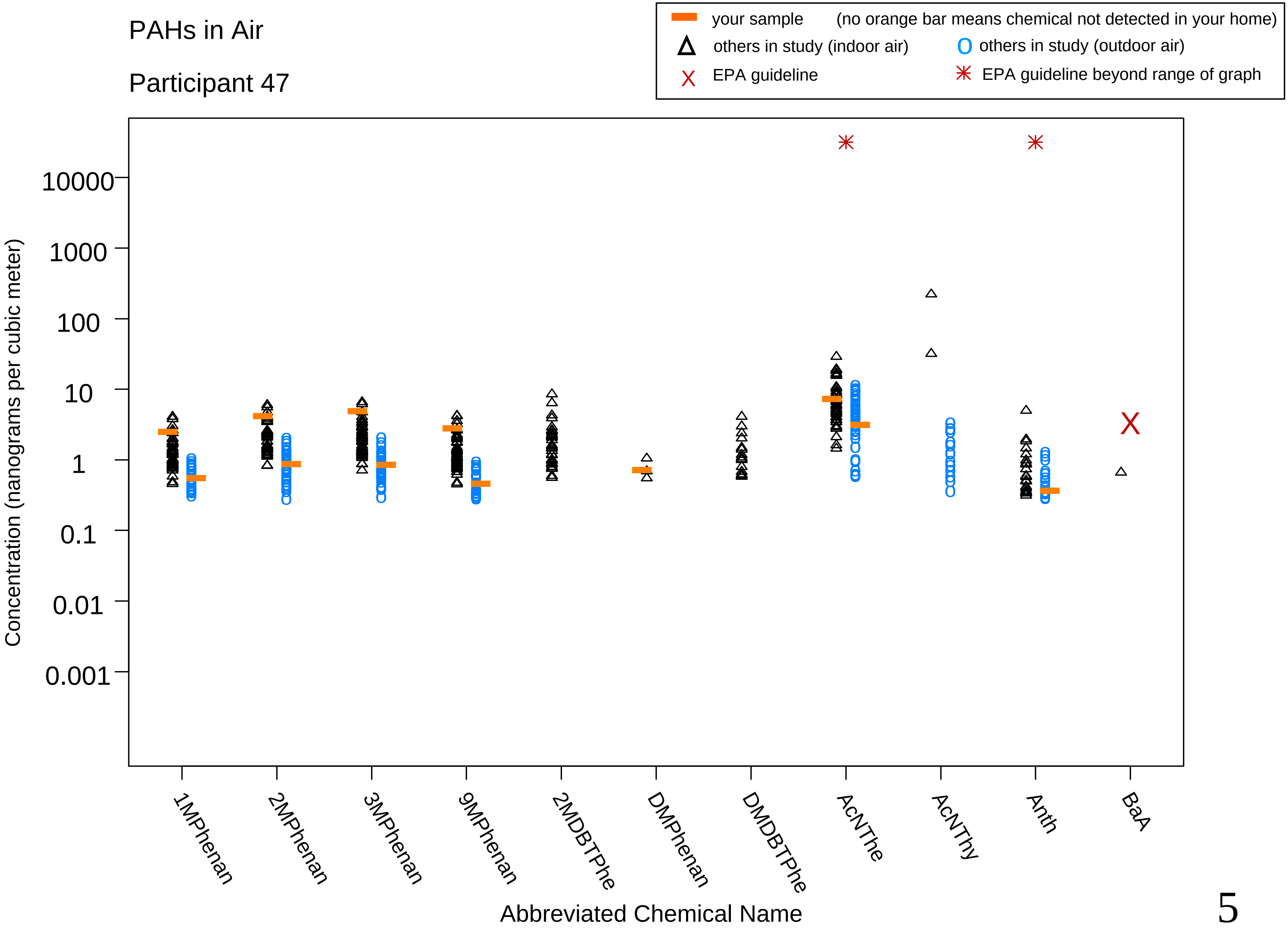

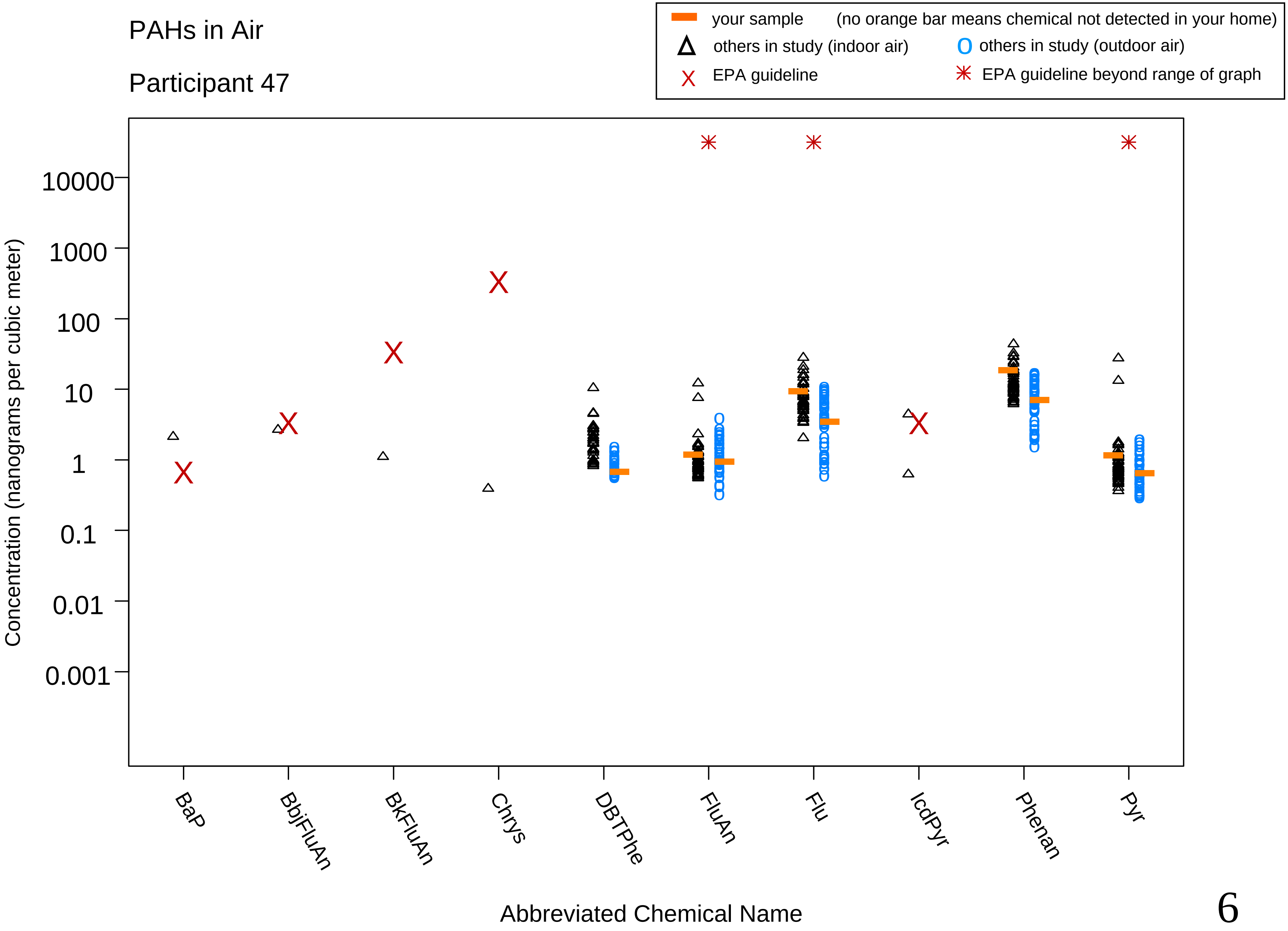

PAHs in Air
Participant 47
your sample (no orange bar means chemical not detected in your home)
others in study (indoor air)
others in study (outdoor air)
EPA guideline
EPA guideline beyond range of graph
Concentration (nanograms per cubic meter)
10000
1000
100
10
1
0.1
0.01
0.001
BaP
BbjFluAn
BkFluAn
Chrys
DBTPhe
FluAn
Flu
IcdPyr
Phenan
Pyr
Abbreviated Chemical Name

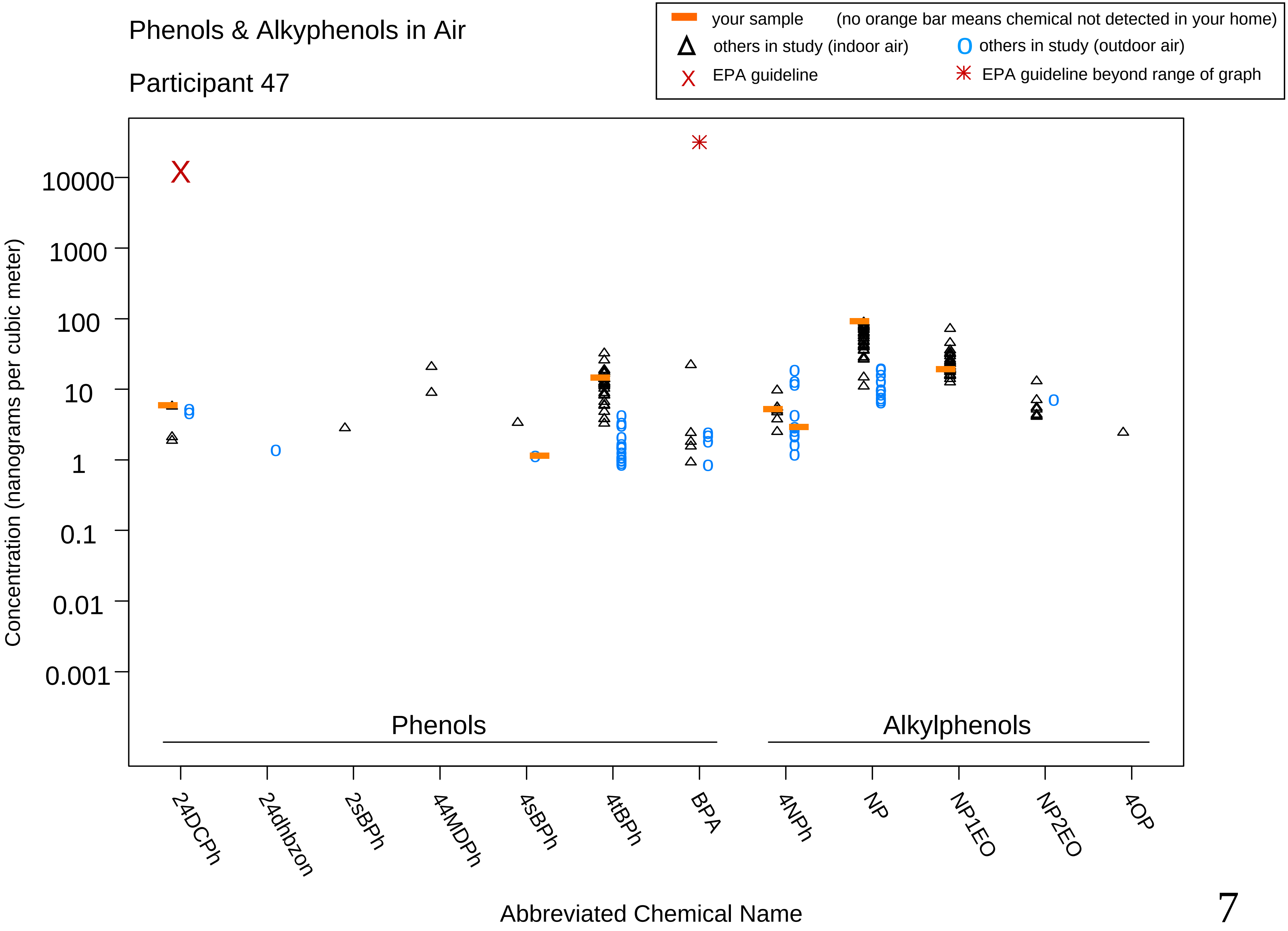

Phenols & Alkyphenols in Air
Participant 47
your sample
(no orange bar means chemical not detected in your home)
others in study (indoor air)
others in study (outdoor air)
EPA guideline
EPA guideline beyond range of graph
Concentration (nanograms per cubic meter)
10000
1000
100
10
1
0.1
0.01
0.001
Phenols
Alkylphenols
24DCPh
24dhbzon
2sBPh
44MDPh
4sBPh
4tBPh
BPA
4NPh
NP
NP1EO
NP2EO
4OP
Abbreviated Chemical Name

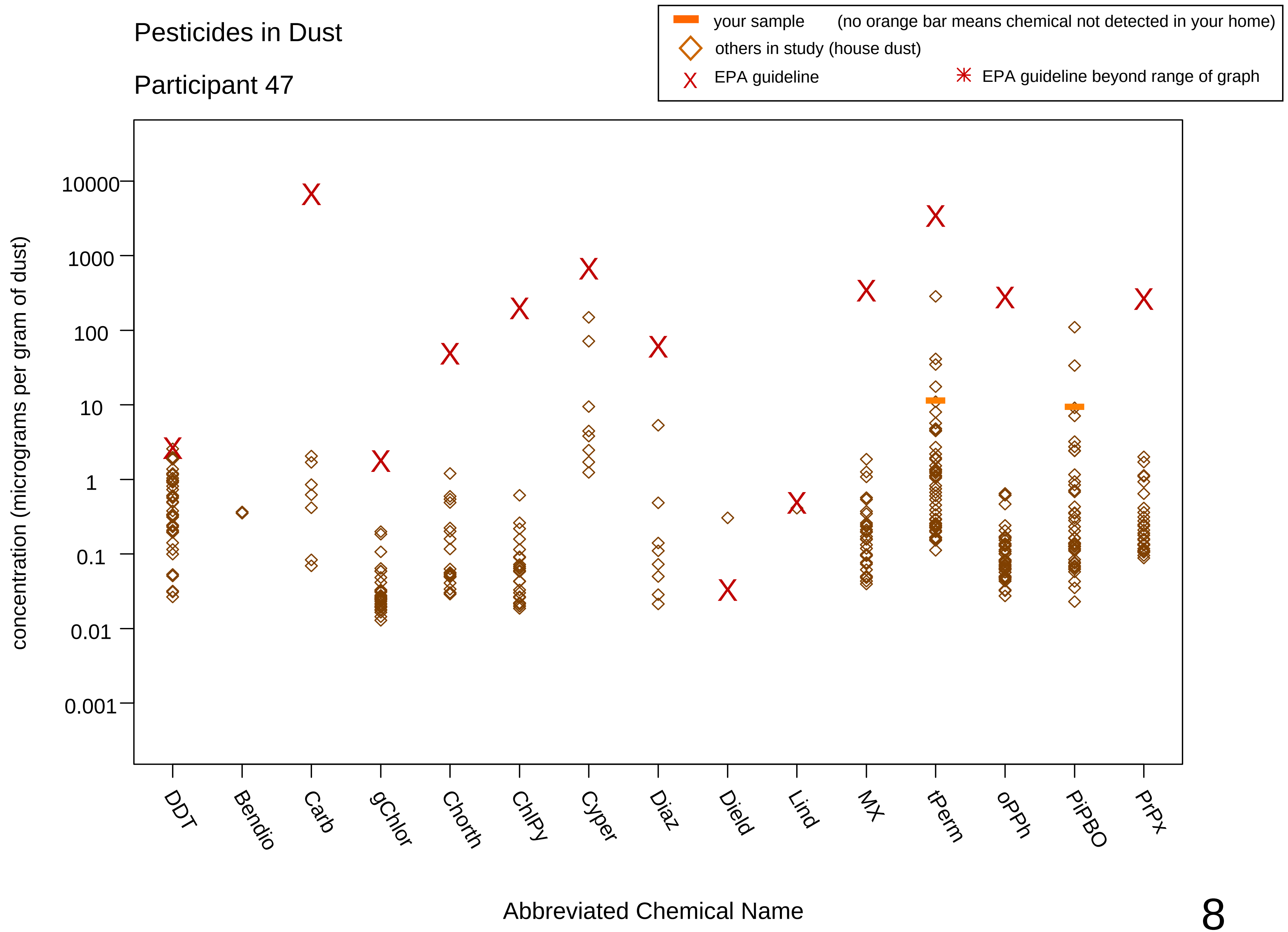
Pesticides in Dust
Participant 47
your sample
(no orange bar means chemical not detected in your home)
others in study (house dust)
EPA guideline
EPA guideline beyond range of graph
concentration (micrograms per gram of dust)
10000
1000
100
10
1
0.1
0.01
0.001
DDT
Bendio
Carb
gChlor
Chorth
ChlPy
Cyper
Diaz
Dield
Lind
MX
tPerm
oPPh
PiPBO
PrPx
Abbreviated Chemical Name

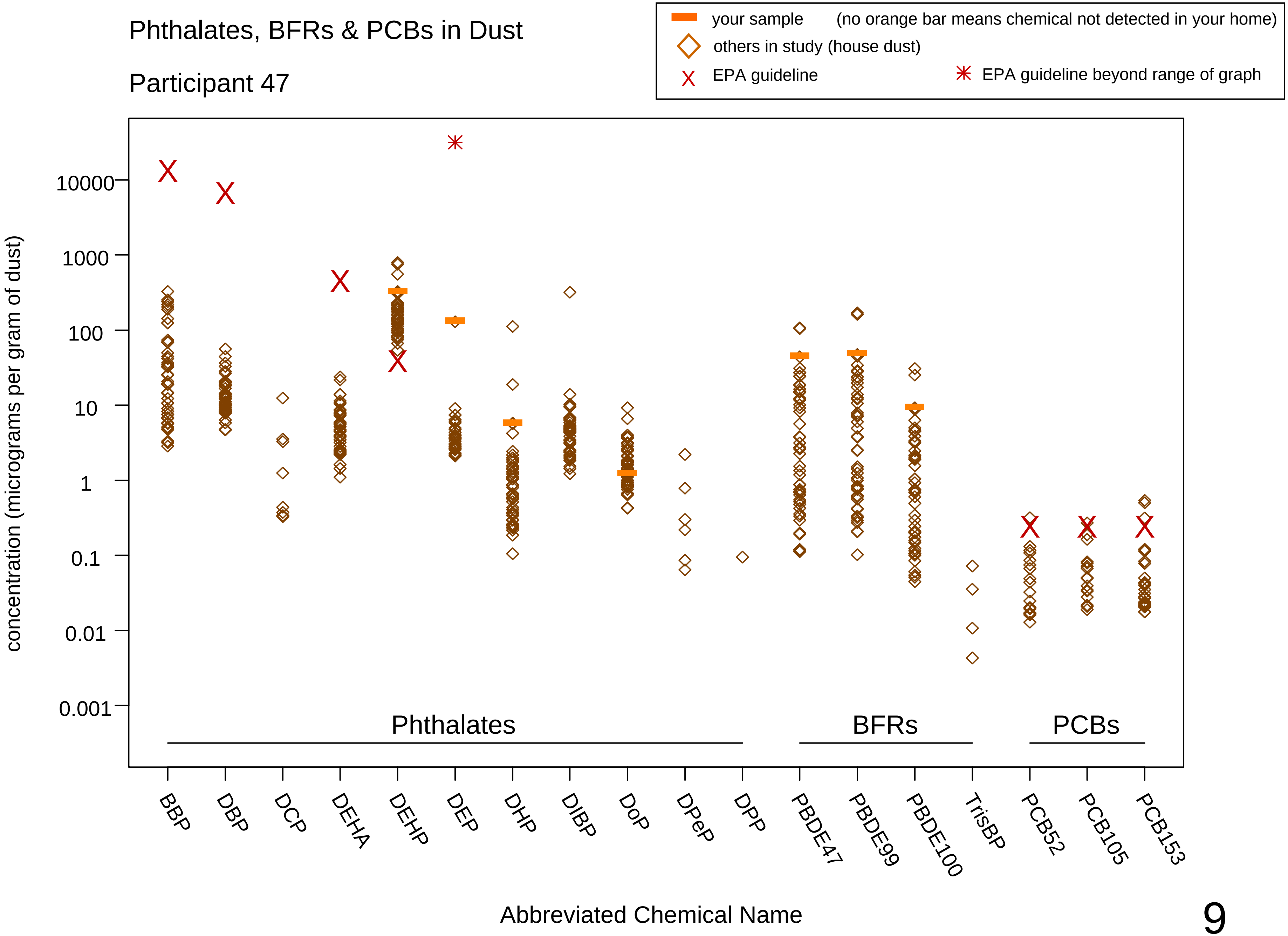

Phthalates, BFRs & PCBs in Dust
Participant 47
your sample (no orange bar means chemical not detected in your home)
others in study (house dust)
EPA guideline
EPA guideline beyond range of graph
concentration (micrograms per gram of dust)
10000
1000
100
10
1
0.1
0.01
0.001
Phthalates
BFRs
PCBs
BBP
DBP
DCP
DEHA
DEHP
DEP
DHP
DIBP
DoP
DPeP
DPP
PBDE47
PBDE99
PBDE100
TrisBP
PCB52
PCB105
PCB153
Abbreviated Chemical Name

PAHs in Dust

Participant 47

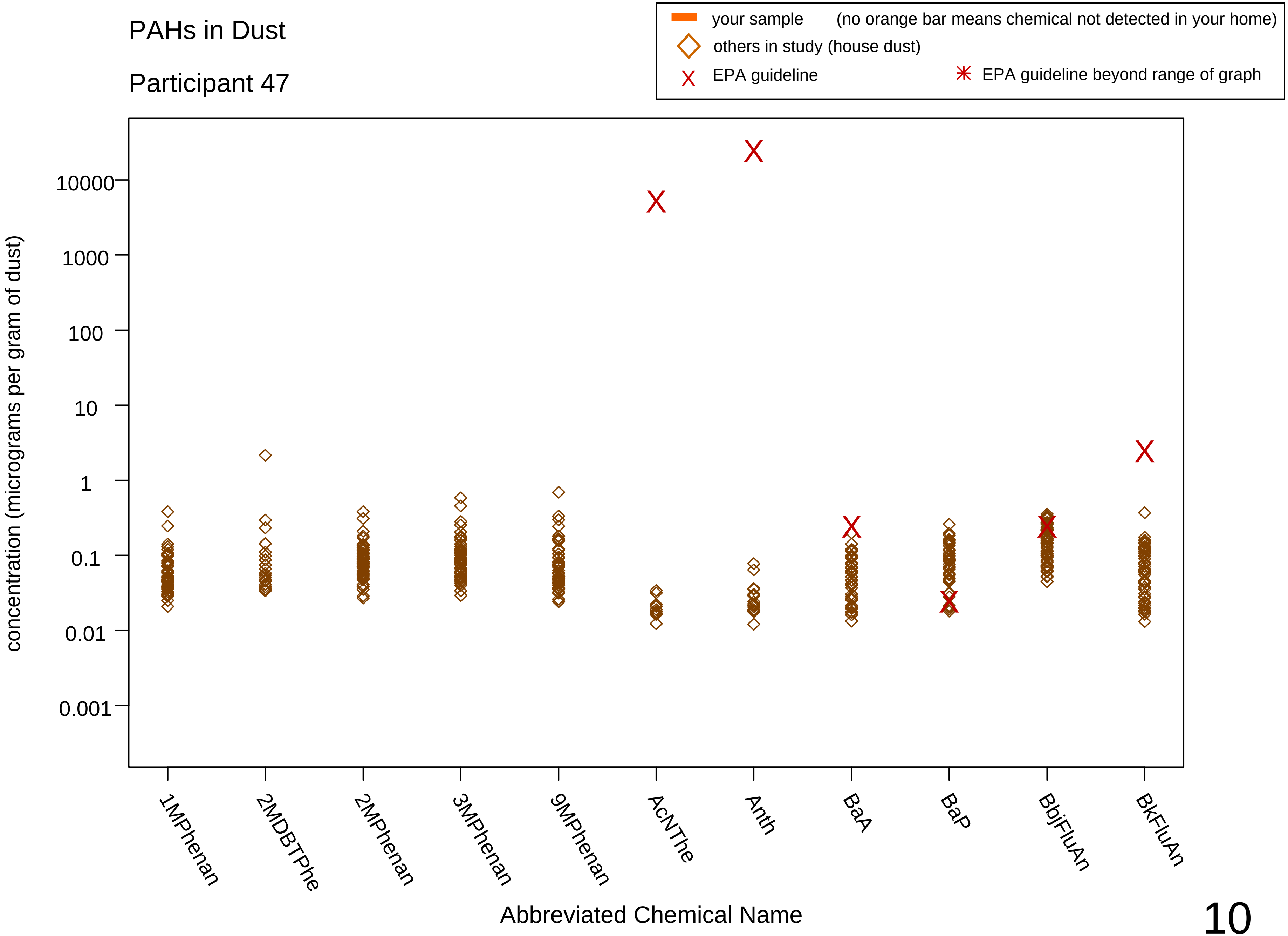

PAHs in Dust

Participant 47

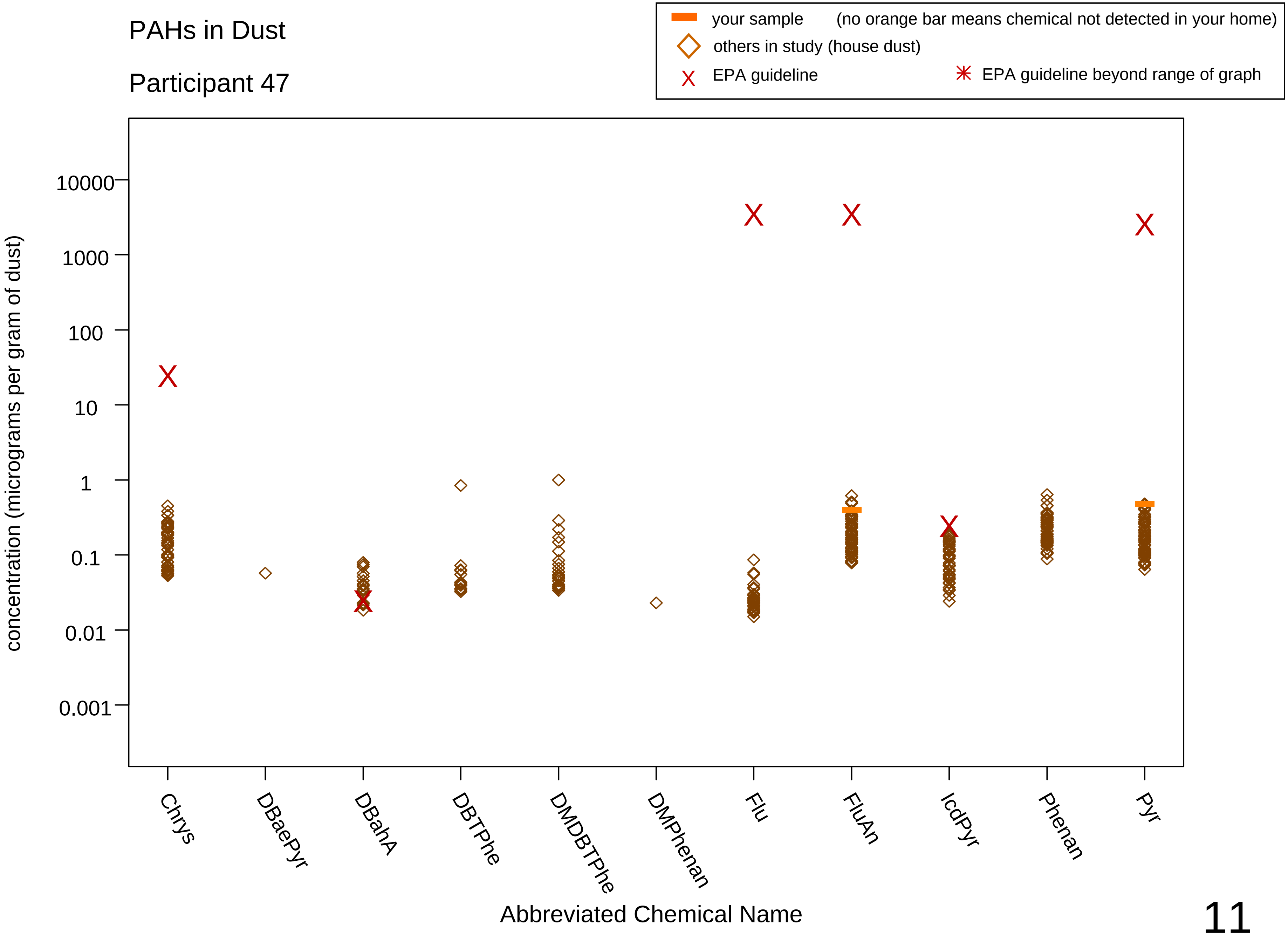

How to Read This Graph

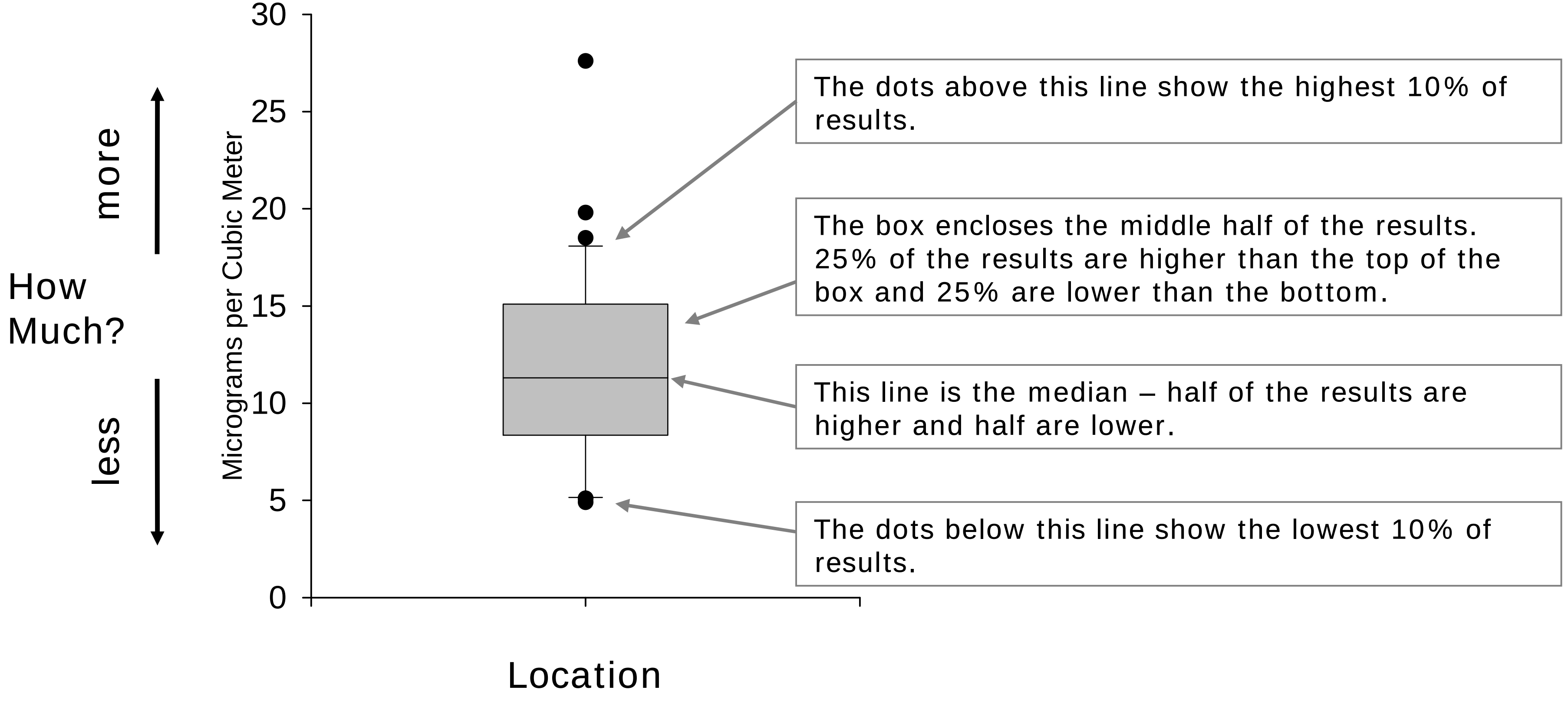

Cómo interpretar esta gráfica

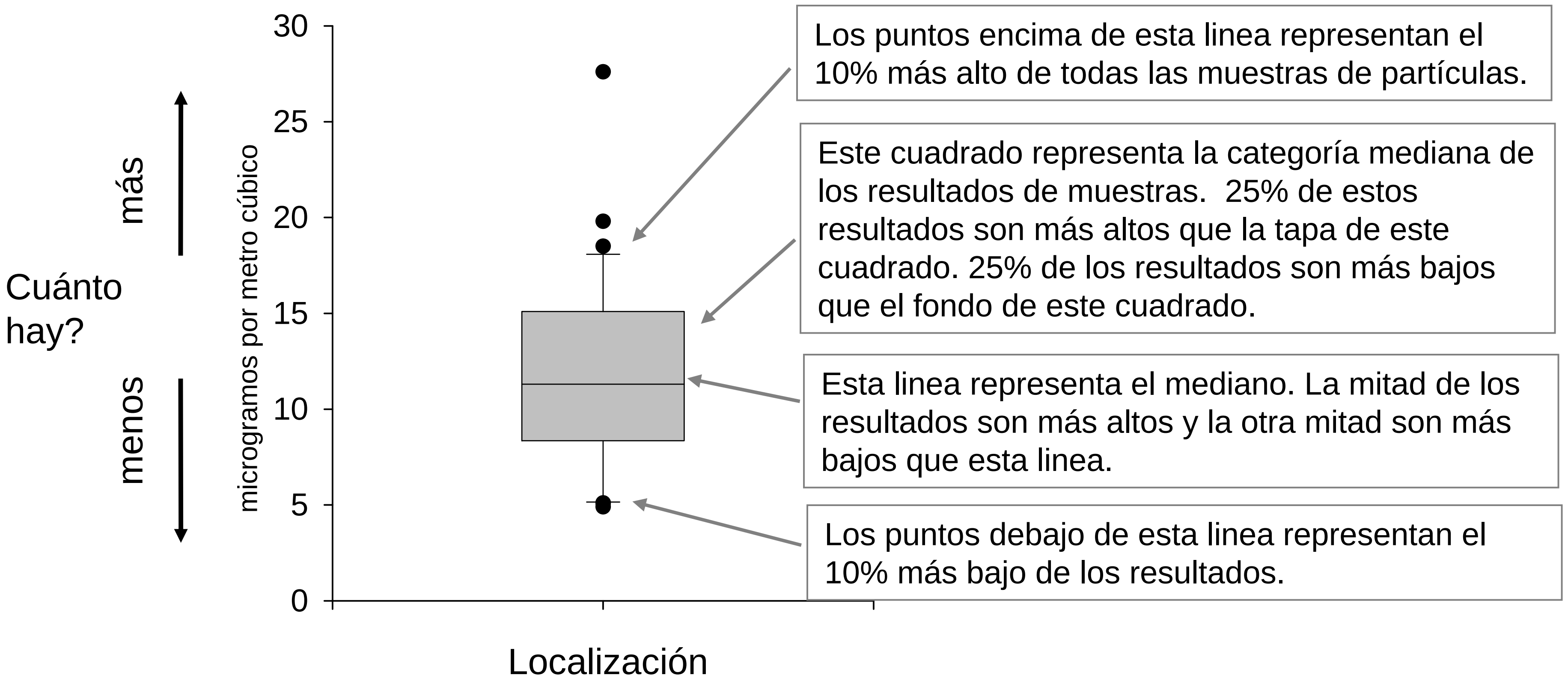

Particulate Air Pollution ($PM_{2.5}$): Richmond and Bolinas

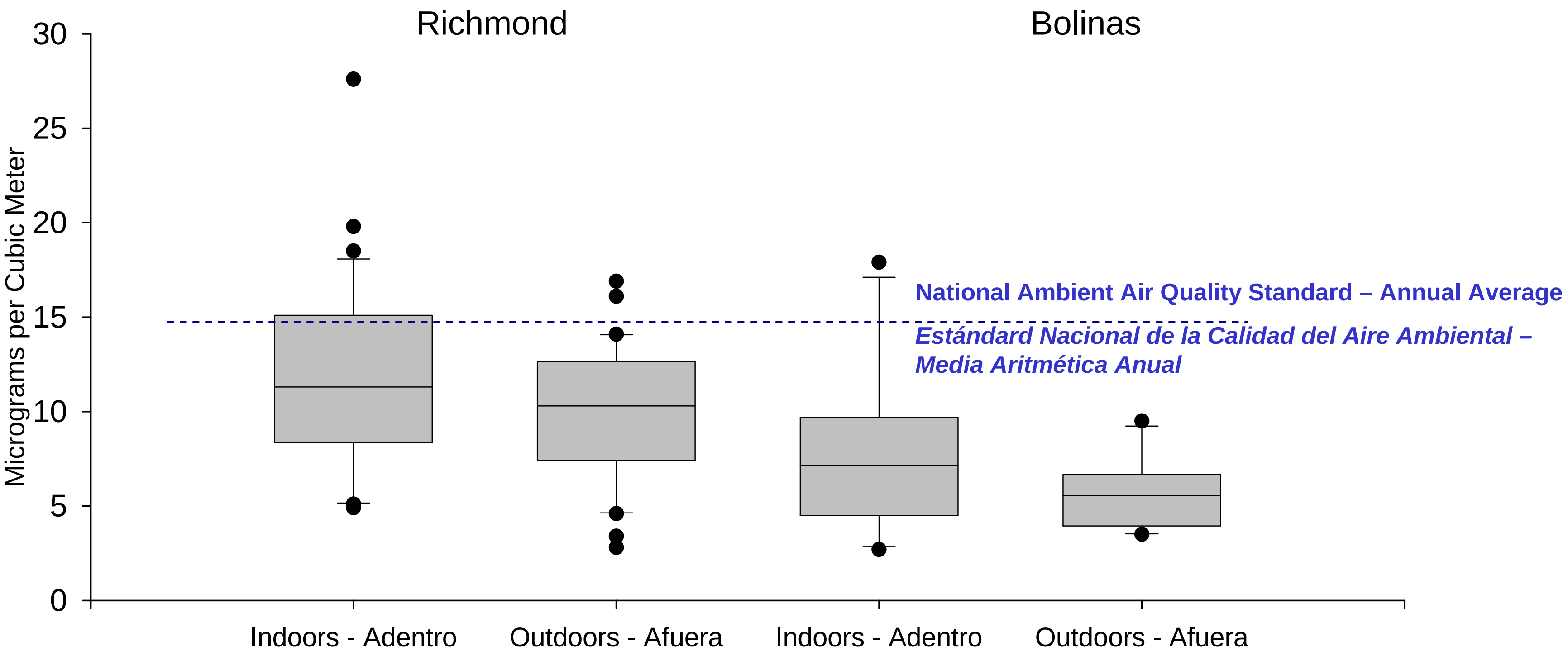

- Particulate levels in Richmond are higher than Bolinas
    - *Los niveles de contaminación son más altos en Richmond*
- Indoor levels are higher than outdoor in both locations
    - *En general, los niveles de contaminación son más altos adentro de las casas que afuera.*
- Particulates can cause respiratory problems, heart problems, and aggravated asthma
    - *Las partículas pueden causar problemas respiratorios, problemas del corazón, y agravar el asma*

Contaminación de partículas (PM2.5) en el aire: Richmond y Bolinas

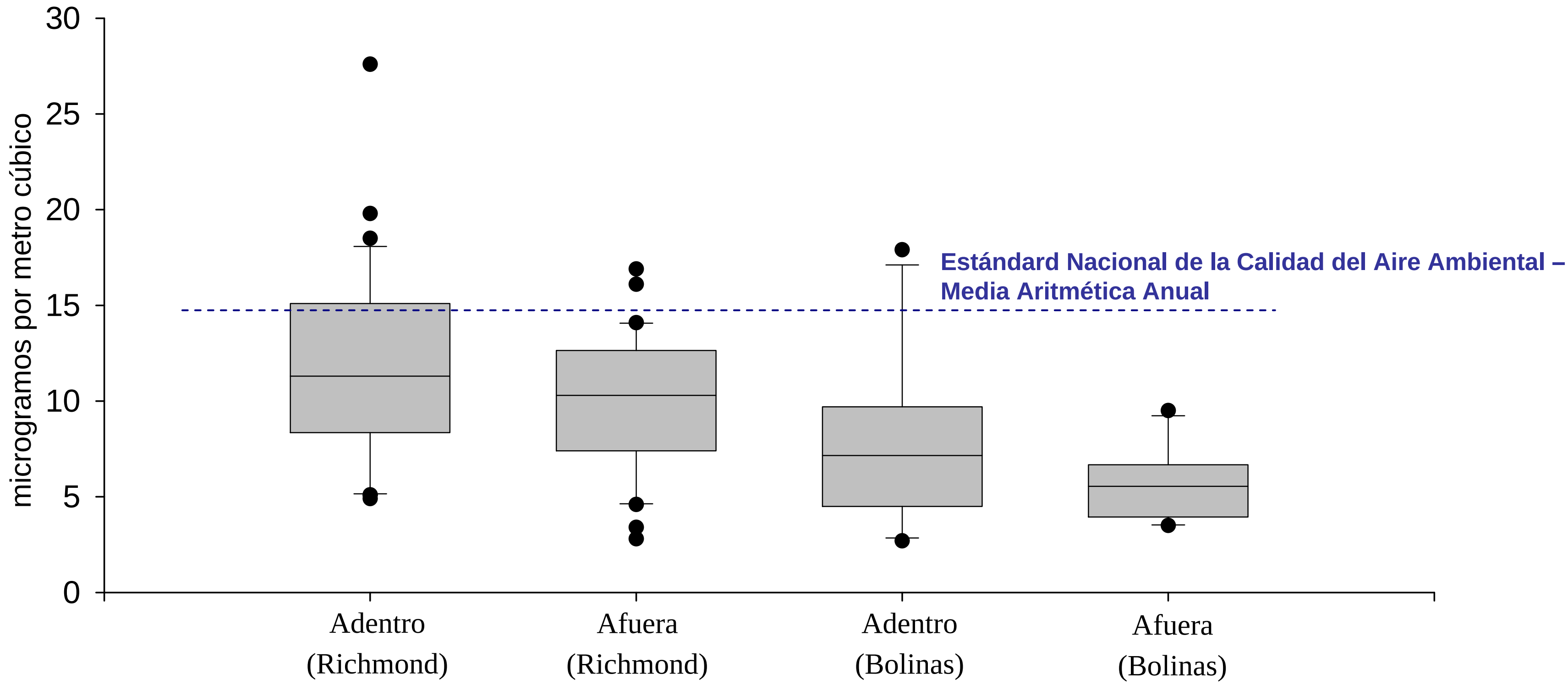

- Los niveles de contaminación son más altos en Richmond.

- En general, los niveles de contaminación son más altos adentro de las casas que afuera.

- Las partículas pueden causar problemas respiratorios, problemas del corazón, y agravar el asma.

Sources of chemicals and amount detected in the CA Household Exposure Study

Chemical	Abbreviation	What is the source? How is it used?	In how many homes did we find it?		
			Number of Detects (%)		
			Indoor Air	Outdoor Air	Indoor Dust
**Pesticides**			50 Homes Tested	43 Homes Tested	49 Homes Tested
4,4'-DDT	DDT	Widely used insecticide (bug killer) prior to 1972 when EPA banned all uses in the US except for public health emergencies. Currently used outside the US for disease control (malaria and other insect transmitted diseases).	12 (24%)	0	42 (86%)
bendiocarb	Bendio	Insecticide (bug killer) used for cockroaches, soil insects, mosquitoes, flies, wasps, ants, fleas. All bendiocarb products voluntarily cancelled in 2001.	-	-	2 (4%)
carbaryl	Carb	Insecticide (bug killer) used for crops, livestock, poultry, household, garden, lawn, and pets; molluscicide; veterinarian medication; former use (sewage treatment plants). Trade name is Sevin.	-	-	7 (14%)
chlordane	gchlor	Former insecticide (bug killer) used for vegetables, termites, lawns, and fruit. Major uses including termite control were stopped in 1988.	16 (32%)	1 (2%)	30 (61%)
chlorothalonil	Chorth	Fungicide (to treat plant diseases) used for vegetable crops, peanuts, lawns, and paint; wood preservative.	6 (12%)	2 (5%)	20 (41%)
chlorpyrifos	ChlPy	Insecticide (bug killer) used for ticks, mosquitoes, soil pests, other household pests, in animal houses, stored products, foliage, corn, alfalfa, cotton, sorghum, citrus, deciduous fruits, and nuts. EPA restricted residential and indoor uses in 2000.	28 (56%)	2 (5%)	25 (51%)
cypermethrin	Cyper	Insecticide (bug killer) used for mosquitoes, cockroaches, houseflies, crops; veterinarian medication.	-	-	8 (16%)
diazinon	Diaz	Insecticide (bug killer) used for over-the-counter ant and roach sprays, garden and lawn sprays, vegetable crops, tobacco, corn, citrus. Sales for residential use were banned by EPA in 2004, but limited use continues.	6 (12%)	1 (2%)	8 (16%)
dieldrin	Dield	Former insecticide (bug killer) used for malaria, locusts, termites, corn, and citrus; termite-proofer; timber preservation. In 1974 EPA restricted its use to termite control, non-food seed and plant treatment, and nonagricultural applications. Not registered for current use in the United States.	-	-	1 (2%)

Sources of chemicals and amount detected in the CA Household Exposure Study

Chemical	Abbreviation	What is the source? How is it used?	In how many homes did we find it?		
			Number of Detects (%)		
			Indoor Air	Outdoor Air	Indoor Dust
heptachlor	Hept	Former insecticide (bug killer) used for agricultural crops, lawn and garden, termite control, seed treatment; repellant spray used for flies, fleas, and mosquitoes. Most registered uses were cancelled in 1978.	2 (4%)	1 (2%)	-
HPTE	HPTE	Breakdown product of methoxychlor (see methoxychlor).	1 (3%)*	0**	-
lindane	Lind	Former insecticide (bug killer) used for plant-eating and soil-inhabiting insects in crops and seed treatments, public-health pests such as lice, scabies mites, and animal skin parasite; indoor animal treatment; rodent control. EPA cancelled remaining registrations in 2006.	-	-	1 (2%)
malathion	Malth	Insecticide (bug killer) used for fruit flies and mosquitoes in gardens, lawns, crops, golf courses and storage bins; public health (cattle, poultry, dogs, cats, human head and body lice, household insects, and stored grain); mites and ticks; transportation equipment. Registration currently being reviewed by the EPA.	0	1 (2%)	-
methoxychlor	MX	Former insecticide (bug killer) used for flies, mosquitoes, and other pests in cattle, goats, sheep, pigs, fruit and shade trees, vegetables, vines, flowers, and forestry; DDT replacement in animal houses, dairies, homes, and industrial premises. Use cancelled by the EPA in 2002.	-	-	29 (59%)
pentachlorophenol	PCPh	Insecticide (bug killer); herbicide (weed killer); molluscicide; fungicide; algacide; germicide (trays in mushroom houses); common wood preservative. No longer available for over-the-counter sale in the United States but currently registered for use in United States pending pre-Registration Eligibility Decision by the EPA.	2 (6%)	0**	-
trans-permethrin	tPerm	Insecticide (bug killer) used for crops, livestock, household pests (ants cockroaches), mosquitoes, head and body lice; nematocide; acaricide; wood preservative; tick repellent; medication; veterinarian medication.	2 (4%)	1 (2%)	48 (98%)

Sources of chemicals and amount detected in the CA Household Exposure Study

Chemical	Abbreviation	What is the source? How is it used?	In how many homes did we find it?		
			Number of Detects (%)		
			Indoor Air	Outdoor Air	Indoor Dust
o-phenylphenol	oPPh	Fungicide for crops; germicide; fumicide; household disinfectant; preservative (stains, paints, metal working fluids, textiles, adhesives, cleaning products); ingredient of dyes.	28 (56%)	20 (47%)	47 (96%)
piperonyl butoxide	PipBO	Insecticide synergist (enhances the insecticidal properties of pyrethrins) used for household pests, crops, livestock, storage facilities, mosquito control; medication.	6 (12%)	0	43 (88%)
propoxur	PrPx	Insecticide (bug killer) used for cockroaches, ants, hornets, flies, mosquitoes, wooly aphids, bugs, and leaf hoppers; molluscicide; veterinarian medication.	7 (14%)	0	28 (57%)
trifluralin	Trifl	Herbicide (weed killer) used for crops, yards, and houses.	0	1 (2%)	-
**Particulate Matter, Ions and Metals**			**42 Homes Tested**	**42 Homes Tested**	**No Homes Tested**
particulate matter	PM2.5	Automobile emissions, petroleum refining, power plants, cigarette smoke, home heating, frying food, and burning fossil fuels, candles and incense.	42 (100%)	42 (100%)	-
elemental carbon	EC	See particulate matter.	38 (90%)	30 (17%)	-
ammonia	NH3	Petroleum refining and other industrial emissions; agriculture (fertilizer, manure), automobile exhaust, household cleaners, sewage, and natural sources.	42 (100%)	42 (100%)	-
ammonium	NH4	See ammonia.	0	5 (12%)***	-
nitrate	NO3	Automobile exhaust, petroleum refining, burning fossil fuels, fertilizer, sewage, and natural sources.	9 (21%)	7 (17%)***	-
sulfate	SO4	Shipping, coal combustion (power plants), petrolium refining, and other industrial emissions; burning fossil fuels, natural sources.	21 (50%)	26 (63%)***	-
arsenic	As	Burning fossil fuels, mining, smelting, waste incineration, pesticides, pressure treated wood, and natural sources. Typical air concentrations in urban areas are above the EPA guideline as they were in this study population.	1 (2%)	2 (5%)	-
cadmium	Cd	Burning fossil fuels, mining, smelting, waste incineration, fertilizer, cigarette smoke, and natural sources.	1 (2%)	0	-
chlorine	Cl	Manufacturing (paper bleaching, chlorinated solvents, PVC resins), waste-water treatment, and marine sources.	39 (93%)	35 (83%)	-

Sources of chemicals and amount detected in the CA Household Exposure Study

Chemical	Abbreviation	What is the source? How is it used?	In how many homes did we find it?		
			Number of Detects (%)		
			Indoor Air	Outdoor Air	Indoor Dust
chromium	Cr	Burning fossil fuels, metal processing, waste incineration, and natural sources.	1 (2%)	0	-
lead	Pb	Burning fossil fuels, manufacturing, mining, smelting, waste-incineration, cigarette smoke, older paints, burning lead-wick candles, and natural sources.	4 (10%)	4 (10%)	-
nickel	Ni	Automobile exhaust, petroleum refining, power plants, waste incineration, cement manufacturing, electroplating, cigarette smoke, and natural sources.	20 (48%)	24 (57%)	-
sulfur	S	Petroleum refining, coal combustion (power plants), smelting, burning fossil fuels, fertilizer, and natural sources.	41 (98%)	42 (100%)	-
vanadium	Va	Petroleum refining and other industrial emissions, burning fossil fuels, fertilizer, and natural sources.	30 (71%)	32 (76%)	-
zinc	Zn	Burning fossil fuels, smelting, fertilizer, wood preservatives, paint, road dust, and natural sources.	13 (31%)	14 (33%)	-
**Phthalates**			**50 Homes Tested**	**43 Homes Tested**	**49 Homes Tested**
benzyl butyl phthalate	BBP	Plastic softener and ingredient in polyvinyl chloride (PVC)-based flooring products, adhesives, and other plastics.	13 (26%)	0	48 (98%)
di-n-butyl phthalate	DBP	Plastic softener used in coatings (food and beverage cans, carpets), ingredient in ink, resins and cosmetics (nail polish, perfume, cologne); textile lubricant; carpet backing, paper coatings, adhesives; former insecticide.	50 (100%)	5 (12%)	45 (92%)
dicyclohexyl phthalate	DCP	Plastic softener (synthetic resins); paper finisher; ingredient in water resistant ink.	0	2 (5%)	8 (16%)
bis(2-ethylhexyl) adipate	DEHA	Plastic softener (meat-wrapping, and other plastic food wraps); ingredient in cosmetics (eye shadow, perfumes, cologne, foundations, blush, nail-polish remover, moisturizers, and self tanning products).	50 (100%)	36 (84%)	46 (94%)
bis(2-ethylhexyl) phthalate	DEHP	Plastic softener (shower curtains, rain coats, baby pants, children's toys, floor tiles, household furnishings, food packaging, rubber); ingredient in inks, insect repellant, cosmetics, rubbing alcohol, liquid soap, detergents, and lacquers; paper manufacturing; electric capacitors.	50 (100%)	14 (33%)	49 (100%)

Sources of chemicals and amount detected in the CA Household Exposure Study

Chemical	Abbreviation	What is the source? How is it used?	In how many homes did we find it?		
			Number of Detects (%)		
			Indoor Air	Outdoor Air	Indoor Dust
diethyl phthalate	DEP	Ingredient in varnishes and cosmetics (bath products, perfume, cologne, hair sprays, wave sets, nail polish and remover, detergents, aftershave lotions, skin care products); plastic softener (tooth brushes, children's toys, tools, food packaging); insect repellent; dye carrier; camphor substitute.	45 (90%)	2 (5%)	35 (71%)
di-n-hexyl phthalate	DHP	Plastic softener (cellulose and vinyl plastics).	1 (2%)	2 (5%)	47 (96%)
diisobutyl phthalate	DIBP	Plastic softener.	50 (100%)	37 (86%)	48 (98%)
di-n-octyl phthalate	DOP	Plastic softener (resins and rubber); ingredient in dyes, film, wire, cables, adhesives.	1 (2%)	0	49 (100%)
di-n-pentyl phthalate	DPeP	Plastic softener (nitrocellulose and resin laquers); ingredient in glue and rubber cements.	1 (2%)	0	6 (12%)
di-n-propyl phthalate	DPP	Plastic softener; laboratory use; drug/therapeutic use.	0	0	1 (2%)
**Parabens**			**31 Homes Tested**	**29 Homes Tested**	**No Homes Tested**
butyl paraben	BuPa	Preservative (food and antiseptic creams); antifungal agent.	1 (3%)	0	-
methyl paraben	MePa	Preservative (baked goods, beverages, creams, pastes, jams, jellies, syrups, and cosmetics).	9 (29%)	0	-
**Brominated Flame Retardants**			**50 Homes Tested**	**43 Homes Tested**	**49 Homes Tested**
PBDE 47	PBDE47	Flame retardant used in polyurethane foam for furniture, upholstery, insulation panels, wood imitations, carpet padding, circuit boards, coatings for electrical equipment, military applications, and construction panels.	7 (14%)	0	49 (100%)
PBDE 99	PBDE99	See PBDE 47.	-	-	49 (100%)
PBDE 100	PBDE100	See PBDE 47.	-	-	46 (94%)
tris(2,3-dibromopropyl) phosphate	TrisBP	Former flame retardant used in children's clothing, polyurethane foam for furniture, industrial uniforms, drapes, coatings for electronics, christmas decorations, and polyester thread. Banned in 1977 from use in children's clothing and fabrics and not currently produced in the US.	-	-	4 (8%)

Sources of chemicals and amount detected in the CA Household Exposure Study

Chemical	Abbreviation	What is the source? How is it used?	In how many homes did we find it?		
			Number of Detects (%)		
			Indoor Air	Outdoor Air	Indoor Dust
**Polychlorinated Biphenyls (PCBs)**			**50 Homes Tested**	**43 Homes Tested**	**49 Homes Tested**
PCB 52	PCB52	Former open uses (flame retardants, inks, paints, wood floor finishers, plasticizers, adhesives, wax extenders, dedusting agents, pesticide extenders, lubricants, cutting oils, and carbonless reproducing paper; former closed uses (hydraulic and heat transfer fluids, capacitors, transformers, vacuum pumps, gas-transmission turbines). Open uses were banned in 1977 and closed uses were banned in 1984.	16 (32%)	0	17 (35%)
PCB 105	PCB105	See PCB 52.	6 (12%)	0	16 (33%)
PCB 153	PCB153	See PCB 52.	2 (4%)	0	27 (55%)
**Polycyclic Aromatic Hydrocarbons (PAHs)**			**50 Homes Tested**	**43 Homes Tested**	**49 Homes Tested**
phenanthrene	Phenan	Automobile exhaust, petroleum refining, cigarette smoke, incense smoke, grilling food; burning wood and fossil fuels.	50 (100%)	40 (91%)	48 (98%)
1-methyl phenanthrene	1MPhenan	See phenanthrene.	50 (100%)	27 (63%)	47 (96%)
2-methyl phenanthrene	2MPhenan	See phenanthrene.	50 (100%)	35 (81%)	48 (98%)
3-methyl phenanthrene	3MPhenan	See phenanthrene.	50 (100%)	34 (79%)	47 (96%)
9-methyl phenanthrene	9MPhenan	See phenanthrene.	50 (100%)	24 (56%)	47 (96%)
3,6-dimethyl phenanthrene	DMPhenan	See phenanthrene.	3 (6%)	0	1 (2%)
dibenzothiophene	DBTPhe	Cosmetics, pharmaceuticals; automobile exhaust; burning fossil fuels.	31 (62%)	21 (49%)	12 (24%)
2-methyl-dibenzothiophene	2MDBTPhe	See dibenzothiophene.	35 (70%)	0	24 (49%)
4,6-dimethyl dibenzothiophene	DMDBTPhe	See dibenzothiophene.	18 (36%)	0	22 (45%)
anthracene	Anth	Automobile exhaust, cigarette smoke, incense smoke, home heating, grilling food, paving, waste incineration, burning fossil fuels.	21 (42%)	15 (35%)	17 (35%)
benz(a)anthracene	BaA	Automobile exhaust, cigarette smoke, grilling food, home heating, burning fossil fuels, incense smoke.	1 (2%)	0	42 (86%)
dibenz(a,h)anthracene	DBahA	See anthracene.	**-**	**-**	19 (39%)
pyrene	Pyr	Automobile exhaust, cigarette smoke, incense smoke, home heating; ingredient in dyes and optical brighteners.	50 (100%)	33 (77%)	49 (100%)

Sources of chemicals and amount detected in the CA Household Exposure Study

Chemical	Abbreviation	What is the source? How is it used?	In how many homes did we find it?		
			Number of Detects (%)		
			Indoor Air	Outdoor Air	Indoor Dust
benzo(a)pyrene	BaP	Automobile exhaust, petroleum refining, cigarette smoke, grilling food, incense smoke, burning fossil fuels.	1 (2%)	0	44 (90%)
dibenz(a,e)pyrene	DBaePyr	See pyrene.	-	-	1 (2%)
indeno(1,2,3-cd)pyrene	IcdPyr	Cigarette smoke, home heating, burning fossil fuels, paving.	1 (2%)	0	40 (82%)
fluoranthene	FluAn	Automobile exhaust, petroleum refining, cigarette smoke, grilling food, home heating, incense smoke, waste incineration.	50 (100%)	35 (81%)	49 (100%)
benzo(b)&(j)fluoranthene	BbjFluAn	See fluoranthene.	1 (2%)	0	48 (98%)
benzo(k)fluoranthene	BkFluAn	See fluoranthene.	13 (26%)	0	45 (92%)
acenaphthene	AcNThe	Automobile exhaust, petroleum refining, cigarette smoke, incense smoke, burning fossil fuels, paving, waste incineration.	50 (100%)	43 (100%)	11 (22%)
acenaphthylene	AcNThy	Petroleum refining, burning fossil fuels, waste-incineration.	2 (4%)	15 (35%)	-
chrysene/Iso-chrysene	Chrys	Automobile exhaust, cigarette smoke, home heating, grilling food, incense smoke, fossil fueld combustion, waste-incineration.	1 (2%)	0	47 (96%)
fluorene	Flu	Automobile exhaust, petroleum refining, cigarette smoke, incense smoke; burning biomass and fossil fuels; roofing and paving; waste-incineration.	50 (100%)	41 (95%)	37 (76%)
**Phenols**			**31 Homes Tested**	**29 Homes Tested**	**No Homes Tested**
2,4-dichlorophenol	24DCPh	Ingredient in the herbicide 2,4-D, dyes, moth balls, antiseptics and seed disinfectants.	3 (10%)	2 (7%)	-
2,4-dihydroxybenzophenone	24dhbzon	Sunscreen agent; and ultraviolet absorber in polymers.	0	1 (3%)	-
2-sec-butylphenol	2sBPh	Ingredient in resins, plasticizers, surface-active agents, insecticides, acaricides, and herbicides.	1 (3%)	0	-
4,4'-methylenediphenol	44MDPh	No information available.	2 (6%)	0	-
4-sec-butylphenol	4sBPh	See 2-sec-Butylphenol.	1 (3%)	1 (3%)	-
4-tert-butylphenol	4tBPh	Ingredient in germicides.	31 (100%)	16 (55%)	-
bisphenol A	BPA	Used in production of polyester, epoxy, phenoxy, and polysulfone resins, polycarbonate, and hydroquinone; fungicide; ingredient in flame-retardants and rubber chemicals.	5 (16%)	4 (14%)	-
4-Nitrophenol	4NPh	Industrial manufacturing and processing (drugs, fungicides, dyes), gasoline and diesel exhaust, breakdown product of the insecticide parathion.	5 (16%)	4 (14%)	-

Sources of chemicals and amount detected in the CA Household Exposure Study

Chemical	Abbreviation	What is the source? How is it used?	In how many homes did we find it?		
			Number of Detects (%)		
			Indoor Air	Outdoor Air	Indoor Dust
**Alkylphenols (APEOs)**			**31 Homes Tested**	**29 Homes Tested**	**No Homes Tested**
4-nonylphenol	NP	Surface-active agent (detergents, paints, emulsifiers, pesticides, and herbicides); ingredient in plastics.	31 (100%)	4 (14%)	-
nonylphenol monoethoxylate	NP1EO	See 4-nonylphenol.	30 (97%)	0	-
nonylphenol diethoxylate	NP2EO	See 4-nonylphenol.	9 (29%)	1 (3%)	-
4-octylphenol	4OP	Ingredient in resins, fungicides, bactericides, dyes, adhesives, and rubber chemicals; surface-active agent; plastic softener; antioxidant; fuel oil stabilizer.	1 (3%)	0	-

Notes:

* 31 Homes tested

** 29 Homes tested

*** 41 Homes tested

Silent Spring Institute
Brown University
Communities for a Better Environment

617.332.4288 www.silentspring.org
www.brown.edu
510.302.0430 www.cbecal.org

Household Exposure Study Fact Sheet

WHAT IS THE PURPOSE OF THE STUDY?

Most people spend most of their time at home, so chemicals that people are exposed to at home can be important for health. We are studying chemicals that come from activities inside the home as well as pollutants that may come from outside. The goal is to learn about patterns of exposure inside homes. We are also trying to learn how to reduce household exposures.

WHO IS DOING THE STUDY?

The study is being done by Communities for a Better Environment (CBE), a non-profit environmental health and justice organization; Silent Spring Institute, a non-profit research organization that studies women's health and the environment; and Brown University. The National Institute of Environmental Health Sciences is sponsoring the research.

WHO IS IN THE STUDY AND HOW IS THE STUDY BEING DONE?

We invited residents from the Liberty and Atchison Village neighborhoods in Richmond and from Bolinas to participate in the study. A total of 50 homes are in the study: 40 in Richmond and 10 in Bolinas. Researchers collected air and dust samples from each home and from outdoor areas nearby, and they interviewed participants about the types of household products they use. The air and dust samples will be tested for more than 100 chemicals that are in consumer products or air pollution. By collecting samples in Richmond and Bolinas, we will be able to compare homes near air pollution sources with homes in a more rural area.

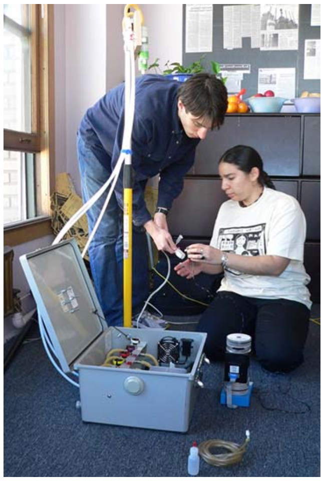

WHAT CHEMICALS IS THE STUDY TESTING FOR?

Metals and particulate matter. Because the Richmond homes in this study are close to industries, such as the Chevron refinery, and to pollution from highways, rail lines, and ship lanes, the study is sampling chemicals associated with those sources, such as metals and particulate matter (small dust that you can breathe into your lungs). These pollutants can affect asthma, other respiratory diseases, and heart health.

Chemicals that may affect hormones. Other chemicals were chosen for this study because of evidence that they may affect hormones. These are known as endocrine disrupting chemicals (EDCs). Some of these chemicals mimic estrogen. They are found in common products, including some pesticides, cleaning products, plastics, furniture, and

cosmetics. Exposure to the natural estrogen made in our bodies increases breast cancer risk, so learning about exposure to other chemicals that mimic estrogen may one day help us learn ways to prevent breast cancer. The chemicals that affect hormones may also affect asthma, fertility, child development in early life and at puberty, learning disabilities, and other aspects of health.

Exposure is an environmental justice concern because low-income communities often have higher asthma rates. Also, African-American women are more likely than others to be diagnosed with breast cancer at an early age and more likely to die of it, even if they have good access to medical care.

WHAT WILL THE STUDY RESULTS SHOW?

Study results will tell us the levels of the chemicals that are found in homes in Richmond and Bolinas. We will be able to compare homes in these two communities with each other and with results from other studies, including homes we tested in Massachusetts. In this study, we will not be able to draw conclusions about the health effects of exposure to the chemicals. Further studies would be needed to determine any link between the pattern of exposure and its health consequences.

HOW WILL RESULTS BE REPORTED?

Individuals who participated in the sampling will have an opportunity to see results for their home if they want to. Summaries of the findings for the communities as a whole will be reported in public meetings and news media as the analysis is completed. The results will also be published in scientific journals. No information that links the personal identity of anyone in the study with the results will be published or shared; individual information will be kept confidential. Right now the laboratory chemical analyses and statistical analyses are in progress. Because this study will yield a great deal of information, additional results will continue to be reported over several years.

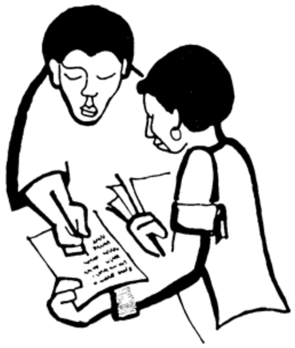

HOW CAN I GET MORE INFORMATION?

Please feel free to contact CBE study coordinator Jessica Tovar or Carla Perez at 510-302-0430.

The study team is grateful to everyone who shared their experiences and opened their homes so that we can learn more about the presence of chemicals in households in these communities and in others. THANK YOU!

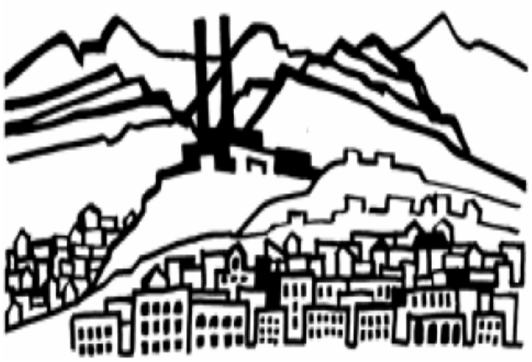

Silent Spring Institute
Brown University
Communities for a Better Environment

617.332.4288 www.silentspring.org
www.brown.edu
510.302.0430 www.cbecal.org

Hoja Informativa del Estudio Sobre la Exposición en el Hogar

¿CUÁL ES EL PROPÓSITO DEL ESTUDIO?

La mayoría de la gente pasa la mayor parte de su tiempo en la casa así que los químicos a los cuales están expuestos las personas en su casa pueden ser importantes para la salud. Estamos estudiando químicos que provienen de actividades dentro del hogar así como contaminantes que pueden provenir de fuera. La meta es aprender sobre las pautas de exposición dentro del hogar. También estamos tratando de aprender cómo reducir las exposiciones dentro del hogar.

¿QUIÉN HACE EL ESTUDIO?

El estudio lo hace *Communities for a Better Environment* (CBE) (Comunidades Para un Mejor Medioambiente), una organización sin fines de lucro para la salud y justicia medioambiental; *Silent Spring Institute*, una organización sin fines de lucro que estudia la salud de las mujeres y el medioambiente; y la Universidad Brown. El Instituto Nacional de Ciencias de la Salud Medioambiental está patrocinando la investigación.

¿QUIÉN PARTICIPA EN EL ESTUDIO Y CÓMO SE ESTÁ HACIENDO EL ESTUDIO?

Invitamos a residentes de los vecindarios de *Liberty* y *Atchison Village en Richmond* y de Bolinas a que participaran en el estudio. Un total de 50 hogares están participando en el estudio: 40 en Richmond y 10 en Bolinas. Los investigadores recogieron muestras del aire y del polvo de cada casa y de las zonas exteriores cercanas y entrevistaron a los participantes sobre los tipos de productos para el hogar que usan. Las muestras del aire y del polvo se analizarán para ver si contienen uno o más de los 100 químicos que se encuentran en productos del consumidor o en la contaminación del aire. Al recoger las muestras en Richmond y en Bolinas podremos comparar las casas que están cerca de las fuentes de contaminación del aire con casas que están en zonas más rurales.

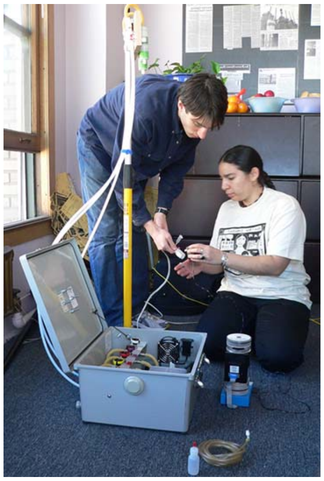

¿CUÁLES QUÍMICOS SE BUSCAN EN EL ESTUDIO?

Metales y Partículas. Debido a que las casas de Richmond en este estudio están cerca de industrias, tales como la refinería de Chevron, y de la contaminación de las carreteras, líneas de tren y rutas de barcos, el estudio está analizando químicos asociados con esas fuentes, tales como metales y partículas (polvo fino que se va a los pulmones al respirar). Estos contaminantes pueden causar asma, otras enfermedades de las vías respiratorias y problemas del corazón.

Químicos que pueden afectar las hormonas. Otros químicos fueron escogidos para este estudio debido a que existe evidencia de que pueden afectar las hormonas. Estos químicos afectan al sistema endocrino, y se llaman *endocrine disrupters* (EDCs) en inglés. Algunos de estos químicos imitan al estrógeno. Se encuentran en productos comunes, incluyendo algunos pesticidas, productos de limpieza, plásticos, muebles y cosméticos. La exposición al estrógeno natural producido por nuestro cuerpo aumenta el riesgo

al cáncer de mama, así que el aprender acerca de la exposición a otros químicos que imitan al estrógeno puede algún día ayudarnos a aprender maneras de prevenir el cáncer de mama. Los químicos que afectan las hormonas también pueden afectar el asma, la fertilidad, el desarrollo infantíl en la niñez y la pubertad, problemas de aprendizaje y otros aspectos de la salud.

La exposición es un problema de la justicia medioambiental porque las comunidades de bajos ingresos frecuentemente tienen índices de asma mayores que otras comunidades. Así mismo, las mujeres afro americanas tienen más posibilidades que otras mujeres de ser diagnosticada con cáncer de mama a una temprana edad y más posibilidades de morir de ello, aunque tengan buen acceso a cuidado médico.

¿QUÉ MOSTRARÁN LOS RESULTADOS DEL ESTUDIO?

Los resultados del estudio nos dirán los niveles de los químicos que se encuentran en los hogares de Richmond y Bolinas. Nos permitirán hacer comparaciones entre hogares de estas dos comunidades y con los resultados de otros estudios, incluyendo hogares que analizamos en Massachussets. En este estudio no podremos sacar conclusiones sobre los efectos de la exposición a los químicos en la salud. Se necesitarían más estudios para determinar el vínculo que existe entre el modelo de la exposición y sus consecuencias médicas.

¿CÓMO SE INFORMARÁ SOBRE LOS RESULTADOS?

Las personas que participaron en las pruebas tendrán la oportunidad de ver los resultados de su hogar si lo desean. Resúmenes de las conclusiones de todas las comunidades serán compartidos en reuniones públicas y en los medios de publicidad cuando se termine el análisis. Los resultados también serán publicados en revistas científicas. No se publicará ni compartirá ninguna información que vincule la identidad de alguien que participó en el estudio con los resultados; la información individual será confidencial. En este momento los análisis de laboratorio de los químicos y los análisis estadísticos están en progreso. Debido a que este estudio dará muchísima información, se continuará informando sobre los resultados adicionales por varios años.

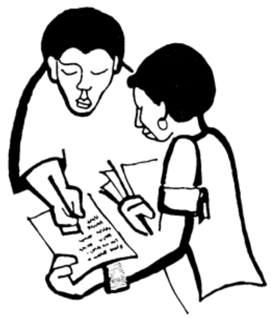

¿CÓMO PUEDO OBTENER MÁS INFORMACIÓN?

Por favor comuníquese con la organizadora comunitaria de CBE Jessica Tovar o Carla Perez al 510-302-0430 x24.

El equipo del estudio agradece a todos los participantes que compartieron sus experiencias y que nos abrieron sus casas para que podamos aprender más sobre la presencia de químicos en los hogares de estas comunidades y otras.

¡GRACIAS!

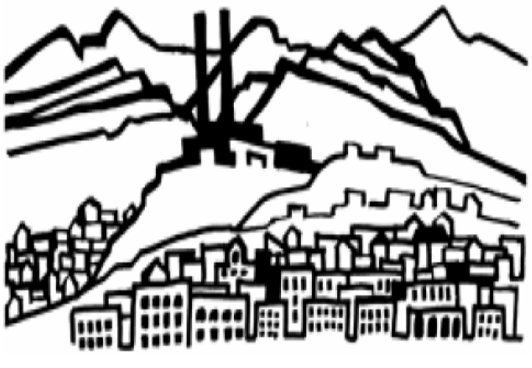

Silent Spring Institute
Brown University
Communities for a Better Environment

617.332.4288 www.silentspring.org
www.brown.edu
510.302.0430 www.cbecal.org

Household Exposure Study Chemicals

Chemical group	Environment and health
**Pesticides**<br><br>*Example:* chlordane	Sources include disinfectants, weed and bug killers used in or near the home, and drift from commercial and agricultural activities.<br><br>Can cause many types of health effects, including effects on brain and reproductive system development and function, hormone systems, ability to fight disease, cancer, and kidney and liver function.
**Phthalates**<br><br>*Example:* dibutyl phthalate	Can be found in vinyl and other plastics, such as children's toys; and also in nail polish, hair spray, and other cosmetics.<br><br>Have been shown to affect hormone systems and cause reproductive harm, especially from exposure during pregnancy.
**Flame Retardants**<br><br>*Example:* PBDE 47	Can be found in children's sleepwear, foam furniture and cushions, mattresses and pillows, synthetic carpets and drapes, and electronic equipment (TVs, computers).<br><br>Have been shown to affect hormone systems and thyroid hormones and cause reproductive harm and effects on learning and behavior in animal studies.
**PCBs**<br><br>*Example:* PCB 52	Sources include older electrical equipment and building materials such as caulks and paints. Banned from new uses in the 1970s but still commonly detected indoors and out.<br><br>Can cause effects on brain development, thyroid hormones, reduced ability to fight disease, hormone disruption, liver damage, and cancer.

**PAHs**<br><br>*Example:* benzo(a)pyrene	Sources include combustion from fireplaces, stoves and heaters, cigarette smoke, outdoor air pollution, and auto exhaust.<br><br>Can cause cancer, reproductive harm, and reduced ability to fight disease.
**Alkylphenols**<br><br>*Example:* Nonylphenol	Can be found in detergents, plastic, and pesticide mixtures.<br><br>Have been shown to affect hormone systems and cause reproductive harm in animal studies.
**Other phenols**<br><br>*Examples:* bisphenol A<br>*o*-phenyl phenol	Can be found in polycarbonate plastic, other plastics, disinfectants, fungicides, and food preservatives.<br><br>Have been shown to affect hormone systems and cause reproductive harm in animal studies.
**Parabens**<br><br>*Example:* methyl paraben	Can be found in cosmetics and skin lotions.<br><br>Have been shown to affect hormone systems in animal studies.
**Metals**<br><br>*Examples:* nickel, lead,<br>vanadium	Sources include industrial emissions, engines, mining, cigarette smoke, and natural processes.<br><br>Can cause many types of health effects, including effects on brain development and functioning, blood pressure, lung irritation, and kidney and liver function.
**Particulate matter**<br><br>*Examples:* soot, PM2.5	Sources include engines, refinery flaring, frying foods, cigarette smoke, and dust.<br><br>Can aggravate asthma and cause breathing and heart problems, resulting in increased death rates.
**Ammonia**	Sources include industrial emissions, fertilizers, cleaning products and natural processes.<br><br>Can cause breathing problems and irritation of skin and eyes.

HOW CAN CHEMICALS GET IN MY BODY? Chemicals can enter your body when you breathe, eat and drink, and through your skin. Chemicals can also be passed from mothers to infants through the placenta and breast milk.

Silent Spring Institute
Brown University
Communities for a Better Environment

617.332.4288 www.silentspring.org
www.brown.edu
510.302.0430 www.cbecal.org

Estudio Sobre la Exposición de los Químicos en el Hogar

Grupo químico	El medioambiente y la salud
**Pesticidas**<br><br>*Ejemplo: chlordane*	Las fuentes incluyen desinfectantes, insecticidas que se usan dentro y cerca del hogar y deriva causada por actividades comerciales y agrícolas.<br><br>Puede causar muchos tipos de efectos médicos, incluyendo efectos en el cerebro y en el desarrollo y función del sistema reproductivo, los sistemas hormonales, la capacidad para combatir las enfermedades, el cáncer y la función de los riñones y el hígado.
**Phthalates**<br><br>*Ejemplo:* dibutyl phthalate	Se encuentra en el vinilo y otros plásticos, tales como los juguetes para niños; y también en el esmalte de uñas, spray para el cabello y otros cosméticos.<br><br>Se ha mostrado que afecta el sistema hormonal y que causa daños reproductivos, especialmente cuando la exposición ocurre durante el embarazo.
**Retardadores de Fuego**<br><br>*Ejemplo:* PBDE 47	Se encuentra en la ropa de dormir de los niños, en muebles y cojines de espuma, colchones y almohadas, alfombras sintéticas y cortinas y en equipo electrónico (teléfonos, computadoras).<br><br>Se ha mostrado que afecta los sistemas hormonales y las hormonas de la tiroides y causa daño reproductivo y tiene efectos en el aprendizaje y la conducta de los animales según los estudios.
**PCBs**<br><br>*Ejemplo:* PCB 52	Las fuentes incluyen equipo eléctrico viejo y materiales de construcción tales como masillas y pinturas. Fueron prohibidos en los años 70 pero todavía se detectan dentro y fuera (de las construcciones).<br><br>Puede causar efectos en el desarrollo del cerebro, en las hormonas de la tiroides, reduce la capacidad de combatir enfermedades, causa desarreglos hormonales, daño al hígado y cáncer.

**PAHs**<br><br>*Ejemplo:* benzo(a)pyrene	Las fuentes incluyen combustión de chimeneas, estufas y calentadores, humo del cigarro, contaminantes del aire exterior y gases de combustión.<br><br>Puede causar cáncer, daño reproductivo y puede reducir la capacidad de combatir enfermedades.
**Alkylphenols**<br><br>*Ejemplo:* Nonylfenol	Se encuentra en detergentes, en el plástico y en mezclas de pesticidas.<br><br>Se ha mostrado que afectan los sistemas hormonales y causan daño reproductivo en los estudios de los animales.
**Otros fenoles**<br><br>*Ejemplos:* bisfenol A<br>*o*-fenyl fenol	Se encuentra en plástico *poli* carbonato, plástico, otros plásticos, desinfectantes, funguicidas y preservativos de comida.<br><br>Se ha mostrado que afectan los sistemas hormonales y causan daño reproductivo en los animales según los estudios.
**Parabenos**<br><br>*Ejemplo:* metil parabeno	Se encuentra en cosméticos y cremas para la piel.<br><br>Se ha mostrado que afecta los sistemas hormonales en los animales según los estudios.
**Metales**<br><br>*Ejemplos:* níquel, plomo, vanadio	Las fuentes incluyen emisiones industriales, motores, minas, humo de cigarro y procesos naturales.<br><br>Puede causar muchos tipos de efectos médicos incluyendo efectos en el desarrollo y funcionamiento del cerebro, la presión arterial, irritación de los pulmones y la función de los riñones y el hígado.
**Partículas**<br><br>*Ejemplos:* hollín, PM2.5	Las fuentes incluyen motores, destellos de refinería, freír comida, el humo del cigarro y el polvo.<br><br>Puede agravar el asma y causar problemas de respiración y del corazón resultando en índices de muerte más altos.
**Amoníaco**	Las fuentes incluyen emisiones industriales, fertilizantes, productos de limpieza y procesos naturales.<br><br>Puede causar problemas de respiración e irritación de la piel y los ojos.

¿CÓMO PUEDEN LOS QUÍMICOS ENTRAR EN MI CUERPO? Los químicos pueden entrar en su cuerpo cuando respira, come y bebe, y por la piel. Los químicos también pueden ser pasados de las madres a los bebés por medio de la placenta y la leche materna.

Ants

Although ants are annoying when they come indoors, they can be beneficial by feeding on fleas, termites, and other pests in the garden. While spraying chemicals inside the house may seem effective, it won't prevent more ants from entering your home because most ants live outdoors. Instead, focus efforts on keeping ants from entering buildings. Combine several methods such as caulking entryways, cleaning up food sources, and baiting when necessary. Avoid the use of pyrethroids (e.g. bifenthrin and cypermethrin), especially on hard surfaces such as driveways, sidewalks, or around the foundation of buildings. These products pollute waterways.

Make your house less attractive to ants.

- **Caulk cracks and crevices that provide entry into the house.**
- **Store attractive food in closed containers.**
- **Clean up grease and spills.**
- **Ant-proof kitchen garbage pails with sticky barriers such as petroleum jelly under the lip and place pet dishes in a moat of water.**
- **Remove or manage sweet food sources next to your house such as aphid-infested bushes and ripened fruit on trees.**
- **Keep plants, grass, and organic mulch at least a foot away from the foundation of buildings to reduce ant foraging and nesting.**

When ants invade your house:

- **Sponge-up invading ants with soapy water as soon as they enter.**
- **Plug up ant entryways with caulk.**
- **Take infested potted plants outdoors and submerge pots in a solution of insecticidal soap and water.**
- **Clean up food sources by wiping up spills or placing food in tight-fitting containers.**
- **Rely on outdoor baits to control the ant colony.**
- **Insecticide sprays should not be necessary.**
- **If you hire a pest control company, ask them to use baits rather than perimeter treatments or monthly sprays.**

ANR UNIVERSITY of CALIFORNIA

How baits work:

Pesticide baits attract worker ants so they will take it back to the nest where the entire colony, including queens, may be killed. The pesticide must be slow acting so workers won't be killed before they get back to the nest.

How to use baits:

Place baits near ant trails and nest openings. Prepackaged or refillable bait stations or stakes are safest and easiest to use. Active ingredients in baits may include boric acid/borate, fipronil, avermectin, sulfluramid, hydramethylnon, or arsenic trioxide. Replace baits when empty and reposition them, or try a different bait product if ants don't appear to be taking it. It may take 5 to 10 days to see fewer ants.

For more information, refer to Pest Notes: Ants at www.ipm.ucdavis.edu.

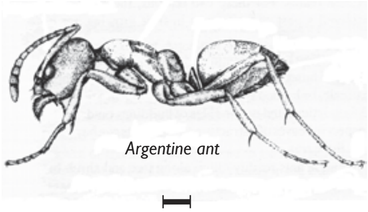

Argentine ant

Actual size 1/8 of an inch.

Minimize the use of pesticides that pollute our waterways. Use nonchemical alternatives or less toxic pesticide products whenever possible. Read product labels carefully and follow instructions on proper use, storage, and disposal.

For more information about managing pests, contact your **University of California Cooperative Extension office** *listed under the county government pages of your phone book or the UC IPM Web site at* **www.ipm.ucdavis.edu.**

UC IPM

University of California Cooperative Extension

What you use in your landscape affects our rivers, and oceans!

Cockroaches

Cockroaches thrive in warm environments that provide food, water, and shelter. Roaches hide in cracks, crawl spaces, and other dark places during the day and come out at night to feed. Pesticide sprays alone will not control roaches and are not usually required. Baits provide better control. You must integrate several strategies to make your home a less roach-friendly environment. Thoroughness is essential for effective control.

Identify your cockroach species first:

- Effective management options vary according to species.
- Cockroach traps provide an easy way to catch roaches for identification.
- Control practices for outdoor invaders (American, oriental roaches) and indoor residents (brown-banded and German roaches) differ.
- For help with identification go to www.ipm.ucdavis.edu

Remove food and water sources:

- Even tiny crumbs or liquids in cracks provide good food sources.
- Store food in sealed containers.
- Keep trash in containers with tight lids.
- Eliminate plumbing leaks.
- Vacuum cracks and crevices and clean floors and counters daily.

Remove roach hiding places:

- Seal cracks and other openings to prevent invaders from the outside.
- Seal cracks in false bottoms of cupboards and other indoor hiding places.
- Seal or clean up other areas where you find roaches or their egg cases hiding.
- Remove old newspapers, boxes and other clutter in kitchens and bathrooms.
- The oriental cockroach hides outdoors under ivy and other shelter. Check to see if you have this roach and remove outdoor hiding places or bait.

ANR UNIVERSITY of CALIFORNIA

Use traps to identify and track cockroach populations:

- Cockroach traps are available in hardware stores.
- Place traps on the floor around edges of walls, in cupboards and other places where you think roaches are foraging. Place bait stations at locations where you trap roaches.
- Check traps daily.
- Sticky traps with pheromones may provide some control of German cockroaches.

Using chemicals to control cockroaches:

- Avoid use of foggers, bombs or aerosol sprays — they just disperse populations.
- Boric acid powder blown into cracks, crevices, hollow walls, under refrigerators, or other undisturbed hiding places is very effective (allow 7 days or more for an effect to be seen).
- Bait stations containing boric acid, abamectin, fipronil, or hydramethylnon placed near hiding places can be effective if other food sources are removed (allow 7 days or more for an effect to be seen). Replace stations as needed as long as roaches are being caught.
- Insecticide sprays alone do not give long term control alone. They are not necessary if other methods such as baits and boric acid powder are combined along with cleanup and removal of hiding places.
- Contact a professional pest control operator for very serious infestations, but be sure they use an integrated program as described above.

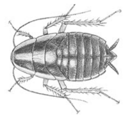

German cockroach nymph

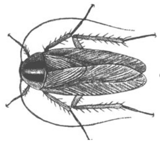

German cockroach adult

Be sure to read product labels carefully and follow all instructions on proper use, storage, and disposal of pesticides.

For more information about managing pests, contact your **University of California Cooperative Extension office** listed under the county government pages of your phone book or the UCIPM Web Site at **www.ipm.ucdavis.edu.**

UC IPM

University of California Cooperative Extension

What you use in your garden affects our creeks, lakes, and rivers!

APPENDIX

B. CHEMICALS IN OUR BODIES STUDY SAMPLE REPORT-BACK PACKET

Date

Name
Address
City, State, Zip

Dear [A01217]:

Thank you very much for taking part in the Chemicals in Our Bodies Project. This project will help us learn more about chemical levels in pregnant women and their babies.

We tested 92 mothers and some of their babies. In some cases, we only have results for mothers, but not their babies, because we were not able to get umbilical cord blood samples at delivery.

When we collected samples in 2010–2011, you asked to receive your results.

Some of your results are ready now and are in this packet. We will send you the results for the other chemicals at a later date.

This packet has three parts:

**Part 1: Metals in Blood**	Includes a summary of results, a results chart, and a table with a list of the metals tested.
**Part 2: Perfluorochemicals in Blood**	Includes a summary of results, a results chart, and a table with a list of the perfluorochemicals tested.
**Part 3: Phenols in Urine**	Includes a summary of results, a results chart, and a table with a list of the phenols tested.

You can compare your results to:

- Results for other mothers and babies in this study.
- National medians. National medians are the middle levels for pregnant women in the U.S. This means that half of U.S. pregnant women tested had chemical levels below the median, and half had levels above the median. Median levels are not necessarily safe.
- Levels of concern. If your level is above a level of concern, we suggest ways to reduce your exposure to protect your health. For most of the chemicals tested, we do not know what level in your body might affect your health.

If you have any questions, please feel free to call (xxx-xxx-xxxx). We are happy to talk with you about your results. We may also contact you to schedule an interview to talk more about your results. Thank you very much for taking part in this project.

Sincerely,

Rachel Morello-Frosch, PhD, MPH, University of California, Berkeley
Rupali Das, MD, MPH, Biomonitoring California, California Department of Public Health
Tracey Woodruff, PhD, MPH, University of California, San Francisco
Project Co-Investigators

Part 1: Metals in Blood Summary of Results for You and Your Baby

Lead

We tested for lead. Lead is a metal that is found in nature and is used in many industries and products.

Did you find lead in my blood?

Yes. We found lead in your blood.

Did you find lead in my baby's blood?

Yes. We found lead in your baby's blood.

Can I compare our levels to other levels?

You can use the *Results Chart* and the Table in this packet to compare your lead levels to:

- **Other mothers and babies in the study**. We found lead in every mother we tested. We found lead in every baby we tested.
- **National median**. The national median is the middle level for pregnant women in the U.S. This means that half of U.S. pregnant women tested had lead levels below the median, and half had levels above the median. Your lead level was less than the national median. The national median for lead in babies is not known.
- **Level of concern**. Your lead level was less than the level of concern. Your baby's lead level was less than the level of concern.

The next page explains more about lead.

More Information about Lead

**Lead is found in**	• Chipped or peeling paint and dust in and around houses built before 1978 (when lead in house paint was banned).<br>• Jobsites in painting, construction, battery recycling, and radiator repair.<br>• Consumer products:<br>– Some handmade glazed dishes<br>– Some imported candies and spices, especially from Mexico and Asia<br>– Some brightly colored medicinal remedies like Azarcon and Greta
**Possible health concerns**	• Lead can affect brain development and contribute to learning problems in babies and young children.<br>• Lead can increase blood pressure, decrease kidney and brain function and cause reproductive problems in adults.<br>• Lead can cause miscarriage and low birth weight.
**Possible ways to reduce exposure**	• Have a trained professional remove or cover old chipped or peeling paint.<br>• Vacuum, wet mop and use a damp cloth to clean your home.<br>• Wash hands before eating or drinking.<br>• Cover bare soil with grass, bark or gravel, especially around houses built before 1978.<br>• If you work with lead, use proper protective equipment and keep work dust out of your home.<br>• Shower after working and wash work clothes separately.<br>• Eat a well-balanced diet that includes foods high in iron and calcium.

For More Information

To have your home checked for lead, call the San Francisco Department of Public Health at 415-252-3956.

If you have concerns about lead and your child, call California's Childhood Lead Poisoning Prevention Program at (510) 620-5600 (www.cdph.ca.gov/programs/CLPPB/Pages/default.aspx)

If you or someone in your family works with lead, call California's Occupational Lead Poisoning Prevention Program at (510) 620-5740 (www.cdph.ca.gov/programs/olppp/Pages/default.aspx)

Cadmium

We tested for cadmium. Cadmium is a metal that is found in nature and is used in many industries and products.

Did you find cadmium in my blood?

No. We did not find cadmium in your blood.

Did you find cadmium in my baby's blood?

No. We did not find cadmium in your baby's blood.

The next page explains more about cadmium.

More Information about Cadmium

**Cadmium is found in**	• Cigarette and other tobacco smoke.<br>• Some cheap metal jewelry and charms.<br>• Rechargeable batteries labeled NiCd or NiCad.<br>• Metal plating and soldering.<br>• Some red, yellow, and orange decorative paints, which may be used on glassware and pottery.
**Possible health concerns**	• Cadmium can affect brain development in young children.<br>• Cadmium can damage the lungs and kidneys.<br>• Cadmium can cause lung cancer.<br>• Cadmium can weaken bones.
**Possible ways to reduce exposure**	• Do not smoke or let children breathe cigarette or other tobacco smoke.<br>• Do not let children wear or play with cheap metal jewelry or charms.<br>• Do not let children handle rechargeable batteries labeled NiCd or NiCad.<br>• Properly recycle batteries.<br>• If you do welding or metal working, be sure the area is well-ventilated and use protective equipment.<br>• Keep children away from welding fumes and other metal vapors and dusts.

For More Information

Cadmium fact sheets: http://www.oehha.ca.gov/public_info/facts/cd_facts.html and http://www.atsdr.cdc.gov/tfacts5.pdf

Battery recycling information: Call 1-(800) CLEANUP (253-2687) or go to http://earth911.com/

Cadmium in consumer products: Call the Consumer Product Safety hotline, 1-800-638-2772

Mercury

We tested for mercury. Mercury is a metal found in nature. It gets into the environment from coal burning, other industries, and abandoned gold mines. Mercury builds up in certain types of fish.

Did you find mercury in my blood?

Yes. We found mercury in your blood.

Did you find mercury in my baby's blood?

Yes. We found mercury in your baby's blood.

Can I compare our levels to other levels?

You can use the *Results Chart* and the Table in this packet to compare your mercury levels to:

- **Other mothers and babies in the study**. We found mercury in every mother we tested. We found mercury in every baby we tested.
- **National median**. The national median is the middle level for pregnant women in the U.S. This means that half of U.S. pregnant women tested had mercury levels below the median, and half had levels above the median. Your mercury level was greater than the national median. The national median for mercury in babies is not known.
- **Level of concern**. Your mercury level was less than the level of concern. Your baby's mercury level was less than the level of concern.

The next page explains more about mercury.

More Information about Mercury

**Mercury is found in**	• Certain types of fish and seafood.<br>• Some imported face creams used for skin lightening, anti-aging or acne.<br>• Silver-colored dental fillings.<br>• Glass thermometers, older barometers, and blood pressure gauges.<br>• Fluorescent lights, including compact fluorescent light (CFL) bulbs.
**Possible health concerns**	• Mercury can affect brain development and cause learning and behavior problems in babies exposed in the womb and in children.<br>• Mercury can harm the nervous system and kidneys.<br>• Mercury may affect the heart.
**Possible ways to reduce exposure**	• Choose fish that are lower in mercury, such as salmon, tilapia, trout, canned light tuna, sardines, anchovies, and oysters.<br>• Avoid fish that are high in mercury, such as shark, swordfish, orange roughy, bluefin and bigeye tuna.<br>• Do not use imported skin-lightening, acne treatment or anti-aging creams unless you are certain that they do not contain mercury.<br>• Do not let children play with silver liquid from items such as mercury thermometers.

For More Information

Choosing fish that are lower in mercury: www.oehha.ca.gov/fish/pdf/2011CommFishGuide_color.pdf
Information on mercury in fish that you catch: www.oehha.ca.gov/fish/hg/index.html or call (510) 622-3170
Concerns about mercury exposure: California Poison Action Line www.calpoison.org/home.html or call 1-800-222-1222
Cleaning up mercury spills, such as from broken thermometers or CFL bulbs: www.epa.gov/mercury/spills/

How to read this chart:	
	**Your level** (There is no blue circle if we did not find this chemical in your blood.)
	**Your baby's level** (There is no purple circle if we did not find this chemical in your baby's blood.)
	**Levels of other mothers or babies in the study** (Each circle represents an individual in the study.)
	**National median** (Half of U.S. pregnant women tested were above this level and half were below.)
	**Level of concern** (If your level is above this, we suggest ways to reduce your exposure.)

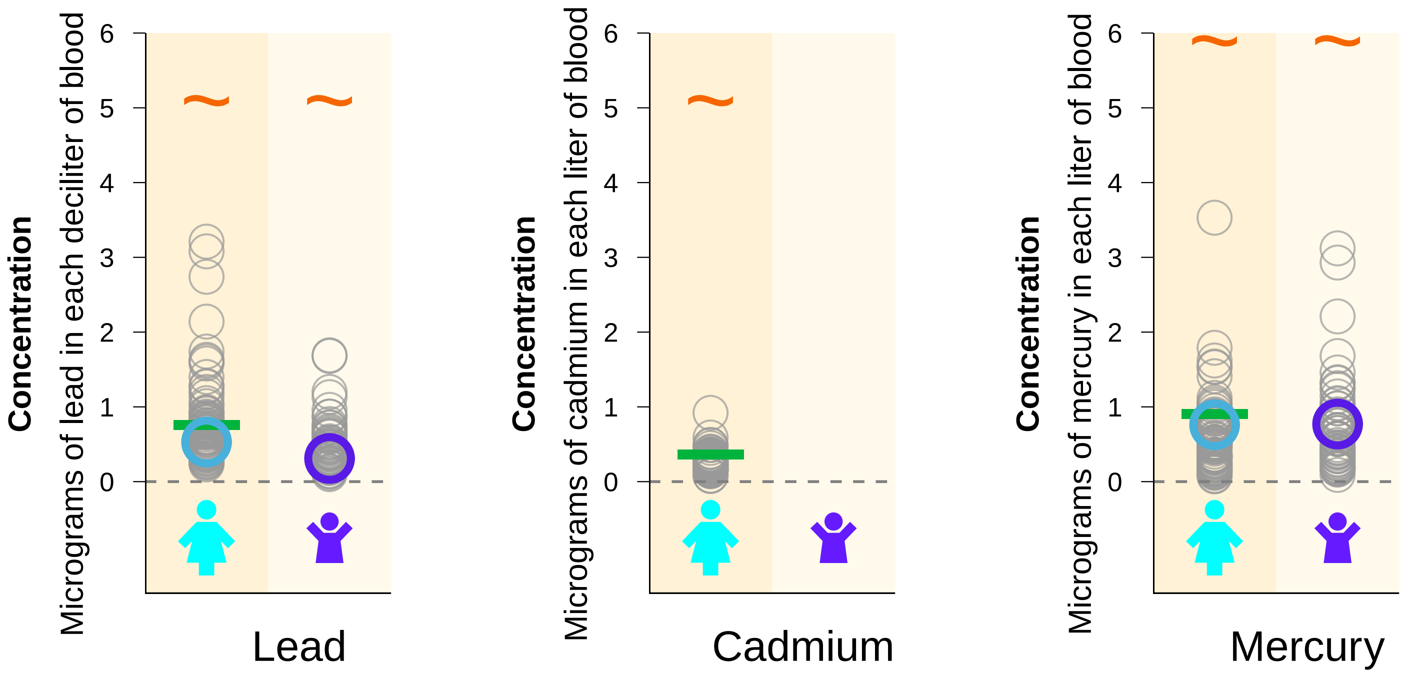

Your exact levels		
Lead	0.53	0.31
Cadmium	Not Found	Not Found
Mercury	0.76	0.77

List of Metals Tested

Explanation of terms:

- **National median**: Half of U.S. pregnant woment tested were above this level and half were below. The national medians for babies are not known.
- **National 95% level**: 95% of U.S. pregnant women tested had levels below this level. This means that in a group of 100 pregnant women, 95 had levels below this level. Only 5%, or 5 out of 100 pregnant women, had levels above this level. The national 95% levels for babies are not known.
- **Level of concern**: If your level is above this, we suggest ways to reduce your exposure.

Metals We Tested in Your Blood

Metal tested	Your level	National median	National 95% level	Level of concern	Number of mothers in this study with this metal in their blood
Lead	0.53	0.61	1.56	5.0	77 of 77 mothers
Cadmium	Not found	0.22	0.96	5.0	65 of 77 mothers
Mercury	0.76	0.76	2.57	5.8	77 of 77 mothers

Metals We Tested in Your Baby's Blood

Metal tested	Your baby's level	National median	National 95% level	Level of concern	Number of babies in this study with this metal in their blood
Lead	0.31	Not available	Not available	5.0	59 of 59 babies
Cadmium	Not found	Not available	Not available	Not available	0 of 59 babies
Mercury	0.77	Not available	Not available	5.8	59 of 59 babies

Lead levels are measured in micrograms per deciliter of blood (ug/dL). Cadmium and mercury levels are measured in micrograms per liter of blood (ug/L).

Part 2: PFCs in Blood Summary of Results for You and Your Baby

Perfluorochemicals (PFCs)

We tested for 12 PFCs. PFCs are used to make products that resist stains, oil, grease and water.

Did you find PFCs in my blood?

Yes. We found 7 PFCs in your blood.

Did you find PFCs in my baby's blood?

Yes. We found 8 PFCs in your baby's blood.

Can I compare our levels to other levels?

You can use the *Results Chart* and the Table in this packet to compare your PFC levels to:

- **Other mothers and babies in the study**. We found PFCs in every mother we tested. We found PFCs in every baby we tested.
- **National median**. The national median is the middle level for pregnant women in the U.S. This means that half of U.S. pregnant women tested had PFC levels below the median, and half had levels above the median. Of the 7 PFCs we found in your blood, 1 was above the national median, and 4 were below. The national medians for the remaining 2 are not known. The national medians for PFCs in babies are not known.
- **Level of concern**. Levels of concern have not been set for PFCs.

The next page explains more about PFCs.

More Information about Perfluorochemicals (PFCs)

**PFCs are found in**	• Some foods, such as red meat and potato chips. Scientists are not sure which foods commonly contain PFCs.<br>• Some grease-repellent paper food containers, such as some microwave popcorn bags, take-out boxes, or fast food wrappers.<br>• Stain-resistant carpets and some carpet cleaning products.<br>• Stain-resistant fabrics and sprays, and waterproofing sprays.<br>• Most non-stick cookware.
**Possible health concerns**	Scientists are still studying how PFCs may affect people's health. There is concern that some PFCs:<br>• May affect the developing fetus and child, including possible changes in growth, learning and behavior.<br>• May decrease fertility and affect hormone balance.<br>• May contribute to cancer.
**Possible ways to reduce exposure**	Scientists are not sure how best to reduce PFC exposures. However, you can:<br>• Limit how often you eat foods from grease-repellent paper containers.<br>• Avoid buying carpets and other items that are labeled "stain-resistant".<br>• Avoid using waterproofing sprays and carpet cleaning solutions that contain PFCs.

For More Information

PFC fact sheet: www.cdc.gov/exposurereport/PFCs_FactSheet.html

How to read this chart:	
	**Your level** (There is no blue circle if we did not find this chemical in your blood.)
	**Your baby's level** (There is no purple circle if we did not find this chemical in your baby's blood.)
	**Levels of other mothers or babies in the study** (Each circle represents an individual in the study.)
	**National median** (Half of U.S. pregnant women tested were above this level and half were below.)

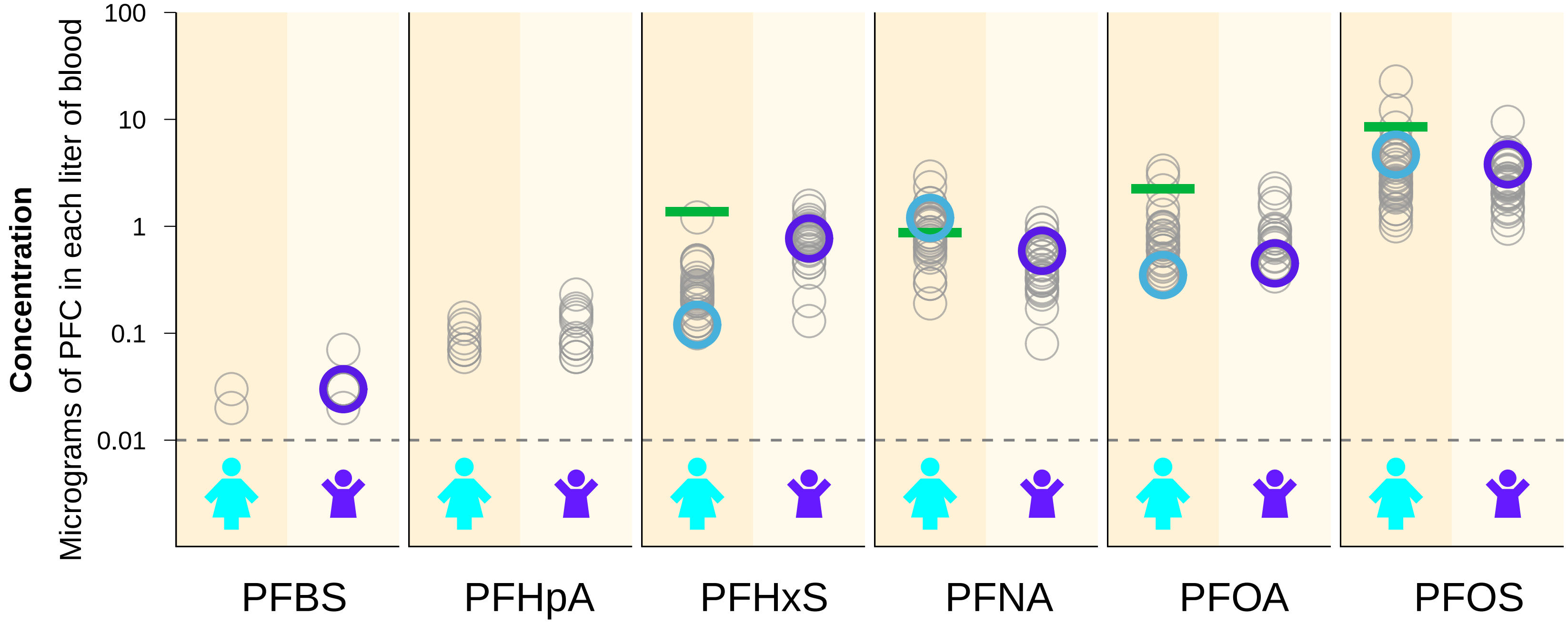

Your exact levels	PFBS (you)	PFBS (baby)	PFHpA (you)	PFHpA (baby)	PFHxS (you)	PFHxS (baby)	PFNA (you)	PFNA (baby)	PFOA (you)	PFOA (baby)	PFOS (you)	PFOS (baby)
	Not Found	0.03	Not Found	Not Found	0.12	0.77	1.20	0.59	0.35	0.45	4.68	3.80

List of Perfluorochemicals (PFCs) Tested

Explanation of terms:

- **National median**: Half of U.S. pregnant woment tested were above this level and half were below.
- **National 95% level**: 95% of U.S. pregnant women tested had levels below this level. This means that in a group of 100 pregnant women, 95 had levels below this level. Only 5%, or 5 out of 100 pregnant women, had levels above this level.
- **Level of concern**: Levels of concern have not been set for PFCs.

PFCs We Tested in Your Blood

PFC tested	Your level	National median	National 95% level	Number of mothers in this study with this PFC in their blood
PFBS	Not found	Not available	Not available	2 of 34 mothers
PFHpA	Not found	Not available	Not available	9 of 34 mothers
PFHxS	0.12	1.10	3.90	32 of 34 mothers
PFNA	1.20	0.70	2.60	34 of 34 mothers
PFOA	0.35	1.80	6.00	27 of 34 mothers
PFOS	4.68	6.80	19.80	34 of 34 mothers
PFDeA	Not found	0.20	0.90	15 of 34 mothers
PFDoA	Not found	Not available	Not available	1 of 34 mothers
PFOSA	0.01	Not available	Not available	33 of 34 mothers
Me-PFOSA-AcOH	0.02	0.40	1.60	34 of 34 mothers
Et-PFOSA-AcOH	Not found	0.14	0.14	24 of 34 mothers
PFUdA	0.23	Not available	Not available	34 of 34 mothers

PFC levels are measured in micrograms per liter of blood (ug/L).

List of Perfluorochemicals (PFCs) Tested

Explanation of terms:

- **National median**: The national medians for PFCs in babies are not known.
- **National 95% level**: The national 95% levels for PFCs in babies are not known.
- **Level of concern**: Levels of concern have not been set for PFCs.

PFCs We Tested in Your Baby's Blood

PFC tested	Your baby's level	National median	National 95% level	Number of babies in this study with this PFC in their blood
PFBS	0.03	Not available	Not available	11 of 30 babies
PFHpA	Not found	Not available	Not available	16 of 30 babies
PFHxS	0.77	Not available	Not available	30 of 30 babies
PFNA	0.59	Not available	Not available	29 of 30 babies
PFOA	0.45	Not available	Not available	21 of 30 babies
PFOS	3.80	Not available	Not available	30 of 30 babies
PFDeA	Not found	Not available	Not available	6 of 30 babies
PFDoA	Not found	Not available	Not available	0 of 30 babies
PFOSA	0.01	Not available	Not available	29 of 30 babies
Me-PFOSA-AcOH	0.02	Not available	Not available	30 of 30 babies
Et-PFOSA-AcOH	Not found	Not available	Not available	25 of 30 babies
PFUdA	0.07	Not available	Not available	30 of 30 babies

PFC levels are measured in micrograms per liter of blood (ug/L).

Part 3: Phenols in Urine Summary of Results for You

Bisphenol A (BPA)

We tested for bisphenol A (BPA). BPA is used to make a hard plastic called polycarbonate. BPA is also used to make protective coatings, like the linings in metal food cans that prevent rust and corrosion.

Did you find BPA in my urine?

Yes. We found BPA in your urine. (We did not test babies for BPA.)

Can I compare my levels to other levels?

You can use the *Results Chart* and the Table in this packet to compare your BPA levels to:

- **Other mothers in the study**. We found BPA in most mothers tested.
- **National median**. The national median is the middle level for pregnant women in the U.S. This means that half of U.S. pregnant women tested had BPA levels below the median, and half had levels above the median. Your BPA level was greater than the national median.
- **Level of concern**. A level of concern has not been set for BPA.

The next page explains more about BPA.

More Information about BPA

**Bisphenol A (BPA) is found in**	• The coatings inside food and drink cans.<br>• Some hard plastic food and drink containers, which might be labeled with the number “7” or “PC” on the bottom.<br>• Some older plastic baby bottles and sippy cups. This use of BPA is ending and will be banned in California by 2013.<br>• Some plastic stretch wrap used to cover or package food.<br>• Some cash register receipts.<br>• Dental sealants and white fillings.
**Possible health concerns**	Scientists are still studying how BPA may affect people’s health. There is concern that BPA:<br>• May affect the fetus and infant, including possible changes in development and behavior.<br>• May affect hormone function.<br>• May affect reproductive function.<br>• May contribute to cancer.
**Possible ways to reduce exposure**	• Eat more fresh food and less canned food.<br>• Use glass or stainless steel containers to store food and liquids.<br>• Avoid using plastic containers for hot food or drinks. Avoid microwaving plastic containers.<br>• Breast-feed your baby when you can. For bottle-feeding, use glass bottles.<br>• Wash hands before eating, because things you touch can have BPA in them.

For More Information

BPA fact sheet for parents: www.hhs.gov/safety/bpa/

Triclosan

We tested for triclosan. Triclosan is used to kill bacteria. It is added to soaps and other consumer products labeled as "antibacterial" or "antimicrobial."

Did you find triclosan in my urine?

Yes. We found triclosan in your urine. (We did not test babies for triclosan.)

Can I compare my levels to other levels?

You can use the *Results Chart* and the Table in this packet to compare your triclosan levels to:

- **Other mothers in the study**. We found triclosan in most mothers tested.
- **National median**. The national median is the middle level for pregnant women in the U.S. This means that half of U.S. pregnant women tested had triclosan levels below the median, and half had levels above the median. Your triclosan level was greater than the national median.
- **Level of concern**. A level of concern has not been set for triclosan.

The next page explains more about triclosan.

More Information about Triclosan

**Triclosan is found in**	• Most antibacterial liquid hand soaps and some antibacterial bar soaps.<br>• Some toothpastes, deodorants, cosmetics, facial cleansers, body washes, and mouthwashes.<br>• Many consumer products, such as some cutting boards, toys, clothes, towels, paint, and garden hoses.
**Possible health concerns**	Scientists are still studying how triclosan may affect people's health. There is concern that triclosan:<br>• May affect hormone function.<br>• May make it harder for antibiotic medicines to fight infections in the body. This is because overuse of triclosan may cause changes in bacteria that make them harder to kill.
**Possible ways to reduce exposure**	• Use regular soap and water to wash your hands. This is just as effective as antibacterial soap.<br>• Avoid products that contain triclosan, unless you have a medical reason for using them.

For More Information

CDC fact sheet: www.cdc.gov/ExposureReport/Triclosan_FactSheet.html

Triclosan in the San Francisco Bay: www.sfei.org/sites/default/files/RMP2011_TriclosanFactsheet_Final4web.pdf

How to read this chart:	
	**Your level** (There is no blue circle if we did not find this chemical in your urine.)
	**Levels of other mothers in the study** (Each circle represents an individual in the study.)
	**National median** (Half of U.S. pregnant women tested were above this level and half were below.)

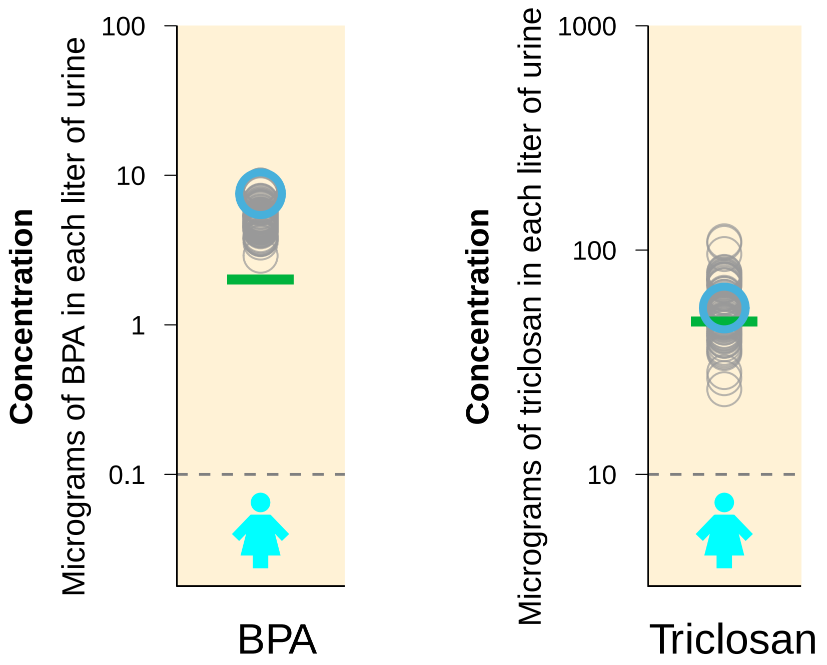

Your exact levels	BPA	Triclosan
	7.53	55.18

List of Phenols Tested

Explanation of terms:

- **National median**: Half of U.S. pregnant woment tested were above this level and half were below.
- **National 95% level**: 95% of U.S. pregnant women tested had levels below this level. This means that in a group of 100 pregnant women, 95 had levels below this level. Only 5%, or 5 out of 100 pregnant women, had levels above this level.
- **Level of concern**: Levels of concern have not been set for phenols.

Phenols We Tested in Your Urine (We Did Not Test Babies for Phenols)

Phenol tested	Your level	National median	National 95% level	Number of mothers in this study with this phenol in their urine
BPA	7.53	1.70	11.70	77 of 77 mothers
Triclosan	55.18	42.90	932.00	77 of 77 mothers

Bisphenol A (BPA) and triclosan levels are measured in micrograms per liter of urine (ug/L).

APPENDIX

C. GREEN CLEANING FACT SHEET

Silent Spring Institute
Brown University
Communities for a Better Environment
University of California Berkeley

GREENING YOUR CLEANING

DID YOU KNOW?

- Many cleaning products can be sources of respiratory irritants, carcinogens, and endocrine disrupting compounds (EDCs – chemicals that can mimic or disrupt hormones).
- Household dust can harbor pollutants such as pesticides, flame retardants, and other chemicals; and allergens.

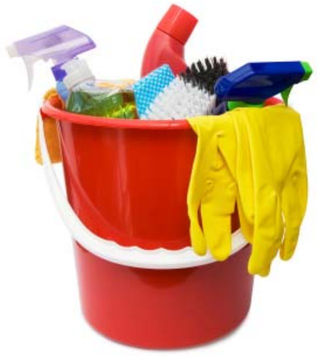

WHAT YOU CAN DO

1) use fewer products
2) keep dust levels low
2) make your own products
3) choose less toxic alternatives

KEEPING DUST LEVELS LOW

Vacuum
Avoid recycling dust back into the air by choosing models with strong suction, rotating brushes, brush on/off switch, a multi-layered bag for dust collection, and a HEPA (high efficiency particulate air) filter. Remember to clean and change the vacuum filter often.

Use a door mat
Pollutants can enter your home on the bottoms of your shoes and on the paws of your pets. To minimize the spread of these pollutants, place a doormat on the outside of each entrance to your home and a washable rug on the inside of each entry; and leave your shoes at the door.

CHOOSING LESS TOXIC CLEANERS

Although some cleaning products are labeled “natural,” “green,” or “non-toxic,” the terms do not necessarily mean the products are safe. Reading the label is the first step; however, because manufacturers do not have to list all ingredients, it isn’t always helpful. Silent Spring Institute is currently testing a range of products to identify safer choices. At this stage, here are some **guidelines to keep in mind when choosing products:**

- Choose mainly plant-based ingredients
- Avoid phthalates (chemicals used to carry fragrance) by reading the label or choosing “fragrance-free”
- Avoid anti-bacterials (e.g., triclosan, Microban) in dishwashing liquid, hand soap, toothpaste, and clothing
- Avoid dichlorobenzene (disinfectant for toilets, garbage cans, and diaper pails)
- Avoid alkylphenol ethoxylates (APEs) in detergents and all-purpose cleaners
- Avoid ethanolamines (e.g., monoethanolamine (MEA), diethanolamine (DEA)) in detergents, all-purpose cleaners, and floor cleaners

MAKING YOUR OWN CLEANERS

(adapted from Women's Voices for the Earth (WVE) "Non-toxic cleaning recipes," available at: http://www.womenandenvironment.org/campaignsandprograms/SafeCleaning/recipes)

All-Purpose Cleaner

suggested uses: hard surfaces like countertops and kitchen floors, windows and mirrors

2 cups white distilled vinegar
2 cups water

Tip: Warming in microwave until barely hot will boost cleaning power for tough jobs. Only microwave in a glass container.

Creamy Soft Scrub

suggested uses: kitchen counters, stoves, bathroom sinks, etc.

2 cups baking soda
½ cup liquid castile soap*

*WVE recommends using a soap that does not contain sodium lauryl (laureth) sulfate (SLS) or Diethanolamine (DEA), which may have harmful side effects.

Tips: For exceptionally tough jobs spray with vinegar first—full strength or diluted—let sit and follow with scrub. Dry soft scrubs can be made with baking soda or salt (or combination of both).

Toilet Bowl Cleaner

Sprinkle toilet bowl with baking soda, drizzle with vinegar, let soak for at least 30 minutes, and scrub with toilet brush.

Tip: Let ingredients soak for a while to make for easy scrubbing, especially on persistent stains like toilet bowl rings

Drain Opener

½ cup baking soda
½ cup vinegar

Pour baking soda down the drain and follow with vinegar. Cover and let sit for at least 30 minutes. Flush with boiling water.

Tip: Prevent your shower from clogging by using a drain trap to catch hair.

Furniture Polish

1/4 cup olive oil
1/4 cup white distilled vinegar
20-30 drops lemon essential oil (2 teaspoons lemon juice may be substituted for lemon oil, but polish must then be stored in refrigerator)

Shake well before using. Dip a clean, dry cloth into the polish and rub wood in the direction of the grain. Use a soft brush to work the polish into corners or tight places.

Tips: To remove water spots rub well with toothpaste. To remove scratches use 1 part lemon juice and 1 part oil, rub with soft cloth.

FOR MORE TIPS AND RECIPES, VISIT:

Silent Spring Institute, www.silentspring.org
Women's Voices for the Earth, www.womenandenvironment.org
Inform Inc., www.informinc.org
Children's Health and Environment Coalition, www.checnet.org
WAGES, www.wagescooperatives.org/eco-house.html (tips in English and Spanish)

Silent Spring Institute
Brown University
Communities for a Better Environment
University of California Berkeley

LIMPIANDO SU CASA ECOLÓGICAMENTE

SABÍA USTED?

- Muchos productos de limpieza pueden ser fuentes de irritantes respiratorios, cancerígenos y compuestos disruptores endocrinos (EDCs – químicos que pueden imitar o perturbar las hormonas).
- El polvo doméstico puede contener contaminantes como pesticidas, retardadores de fuego, y otros químicos; así como también alergénicos.

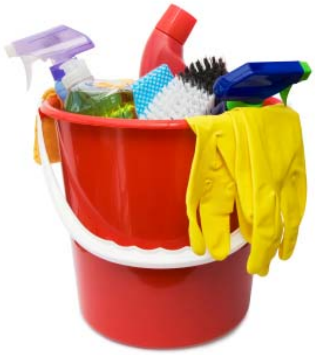

LO QUE USTED PUEDE HACER

1) usar menos productos
2) mantener niveles bajos de polvo
2) fabricar en casa sus propios productos
3) elegir alternativas menos tóxicas

COMO MANTENER BAJOS LOS NIVELES DE POLVO

Aspiradora
Evite que el polvo se devuelva al aire eligiendo modelos con succión fuerte, cepillos rotantes, control on/off para los cepillos, una bolsa para recolección del polvo de varios niveles, y un filtro de aire de alta eficiencia para material particulado conocido como filtro HEPA (high efficiency particulate air). Recuerde limpiar y cambiar el filtro de la aspiradora periódicamente.

Use un tapete a la entrada de su casa
Los contaminantes pueden ingresar a su casa en las suelas de sus zapatos y en las patas de sus mascotas. Para minimizar la propagación de estos contaminantes, ponga un tapete en la parte de afuera de cada entrada de su casa y un tapete lavable en la parte interior de cada entrada; y deje sus zapatos en la puerta.

ELIGIENDO ALTERNATIVAS MENOS TÓXICAS

Aunque algunos productos de limpieza son etiquetados como “naturales”, “verdes (ecológicos)” o “no tóxicos”, estos términos no significan necesariamente que el uso de estos productos es seguro. Leer la etiqueta es el primer paso; sin embargo, debido a que los fabricantes no están obligados a listar todos los ingredientes, la lectura de la etiqueta no siempre es útil. El Silent Spring Institute está analizando actualmente una serie de productos con el objetivo de identificar cuáles son las opciones más seguras. Por ahora, aquí están algunas **pautas que se deben tener en cuenta a la hora de elegir los productos de limpieza:**

- Elija principalmente ingredientes derivados de plantas
- Evite los ftalatos o phtalates (químicos usados como portadores de fragancia) leyendo la etiqueta o eligiendo productos “libres de fragancia”
- Evite los anti-bacteriales (como por ejemplo: triclosan, Microban) en el jabón líquido para lavar los platos, el jabón de manos, la crema dental, y la ropa
- Evite el diclorobenceno o dichlorobenzene (desinfectante para inodoros, botes de basura, y botes para desechar pañales)
- Evite los alquifenoles etoxilados o alkylphenol ethoxylates (APEs) en detergentes y limpiadores multiusos
- Evite los ethanolamines (como por ejemplo: monoethanolamine (MEA), diethanolamine (DEA)) en detergentes, limpiadores multiusos, y limpiadores para el piso

FABRICANDO SUS PROPIOS LIMPIADORES EN CASA

(Adaptado de Women's Voices for the Earth (WVE) "Non-toxic cleaning recipes," disponible en: http://www.womenandenvironment.org/campaignsandprograms/SafeCleaning/recipes)

Limpiador Multiusos

Usos sugeridos: superficies duras como los mesones y los pisos de la cocina, ventanas y espejos

2 tazas de vinagre blanco destilado
2 tazas de agua

Consejo práctico: Calentar la mezcla en el microondas hasta que esté tibia aumenta el poder de limpieza para sucios difíciles. Sólo utilice recipientes de vidrio al usar el microondas.

Limpiador para Fregar Cremoso y Suave

Usos sugeridos: mesones de cocina, estufas, lavamanos, etc.

2 tazas de bicarbonato de sodio
½ taza de jabón de castilla líquido (WVE recomienda el uso de jabones que no contengan lauril sulfato de sodio (sodium lauryl (laureth) sulfate (SLS)) ni Dietanolamina (Diethanolamine (DEA)), los cuales pueden tener efectos secundarios perjudiciales.)

Consejos prácticos: Para sucios excepcionalmente difíciles, rociar con vinagre primero—concentrado o diluido— dejar actuar y refregar en seguida. Se pueden hacer limpiadores para fregar secos y suaves con bicarbonato de sodio o sal (o mezclando los dos).

Limpiador para el inodoro

Rocíe la taza del inodoro con bicarbonato de sodio, después rocíe con vinagre, deje actuar por 30 minutos como mínimo, y refriegue con el cepillo del inodoro.

Consejo práctico: Deje los ingredientes remojar por un rato para que al fregar sea más fácil, especialmente para manchas persistentes como las que se hacen en los bordes de la taza del inodoro

Destapador de Tuberías

½ taza de bicarbonato de sodio
½ taza de vinagre

Vierta el bicarbonato de sodio en el desagüe y después agregue el vinagre. Cubra y deje actuar por 30 minutos como mínimo. Enjuague con agua caliente.

Consejo práctico: Evite que el sifón de su ducha se tape usando una rejilla para atrapar el pelo.

Lustrador de Muebles

1/4 taza de aceite de oliva
1/4 taza de vinagre blanco destilado
20-30 gotas de aceite esencial de limón (se puede sustituir el aceite de limón por 2 cucharaditas de jugo de limón, pero el lustrador debe guardarse en el refrigerador)

Agite bien antes de usar. Unte con la mezcla un paño limpio y seco y frote la madera en dirección de las vetas. Utilice un cepillo suave para esparcir el lustrador en las esquinas o bordes difíciles de alcanzar.

Consejos prácticos: Para eliminar manchas producidas por el agua frote bien con crema dental. Para eliminar rayones mezcle 1 parte de jugo de limón y 1 parte de aceite, frote con un paño suave.

PARA MÁS CONSEJOS PRÁCTICOS Y RECETAS, VISITE:

Instituto Silent Spring (Silent Spring Institute), www.silentspring.org
Voces de las Mujeres por la Tierra (Women's Voices for the Earth), www.womenandenvironment.org
Inform Inc., www.informinc.org
Coalición por la Salud y el Medio Ambiente de los Niños (Children's Health and Environment Coalition), www.checnet.org
WAGES, www.wagescooperatives.org/eco-house.html (Consejos prácticos en Inglés y Español)

APPENDIX

D. TAKE ACTION FACT SHEET

Take Action!

1. Become a CBE member!
   - Receive email or postal alerts on upcoming meetings, events, potlucks and hearings!

   Jessica Tovar at 510-302-0430 x 24 jessica@cbecal.org
   Ana Orozco at 510-302-0430 x 12 aorozco@cbecal.org

2. Testify-Speaking for Ourselves

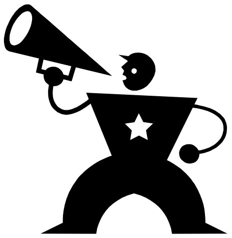

   When you become a member of CBE you will receive alerts on events; including hearings on important issues that affect your community. While CBE works hard to advocate for environmental justice, local decision makers need to hear testimony directly from the community. As a resident living next to industrial facilities; it is important to describe what you see, hear, smell or feel about the facility. The strongest testimonies come from community members who suffer from asthma, headaches, cancer etc. These testimonies have influenced some council members to make decisions based on health and the environment, as opposed to profit.

3. Join us for upcoming events and actions
   - Member orientation ....April 8th (location and time TBA)
   - Member meeting..................................April 14th at St. Mark's Church 6:00 PM
   - Chevron Lawsuit Hearing.....................April 22nd in Martinez (time TBA)
   - "Love Yo Mama"
     Earth Day celebration..........................April 25th at the Tassafaronga Recreation Center and ACORN/Woodland Elementary in East Oakland from 11am to 6pm

4. Speak out against Technical Bulletin 604 (TB 604)
   This standard would regulate the flammability of filled bed clothing including comforters, mattress pads, and pillows. It does not require any health or environmental information about the chemicals and materials that will be used to meet the regulations. Contact Laura Zuniga, Chief, Bureau of Home Furnishings and Thermal Insulation, at bhfti @dca.ca.gov, (916) 574-2041 to say you don't want a bed clothing flammability standard that could lead to toxics in your bed. Go to http://www.bhfti.ca.gov/industry/tb604.shtml to learn about public hearing notice for TB 604.

¡Toma Acción!

1. Ser miembro de CBE – Comunidades para un Medio Ambiente Mejor
   - Reciba emails o alertas por correo sobre reuniones comunitarias, eventos, cenas comunitarias, y demandas legales!

   Jessica Tovar at 510-302-0430 x 24 jessica@cbecal.org
   Ana Orozco at 510-302-0430 x 12 aorozco@cbecal.org

2. Testimonios – Hablando por nosotros mismos
   Cuando sea miembro de CBE, va a recibir alertas sobre eventos incluyendo demandas legales sobre temas importantes que afectan a la comunidad. CBE trabaja duro para abogar por la justicia medioambiental, pero los que hacen las decisiones aquí en Richmond y en el condado de Contra Costa necesitan oír testimonios directamente de la comunidad (ustedes). Como residente viviendo a lado de la industria y fabricas, es importante describir que vea, que oye, y que huele de las fabricas y como se siente como resultado de vivir cerca de las fabricas, como la refinería de Chevron. Los testimonies más Fuertes vienen de miembros de la comunidad que sufren de asma, dolor de cabeza, cáncer, etc. Estos testimonios han influido algunos miembros del consejo para hacer decisiones basado en la salud y el medio ambiente, en lugar de ganancias.

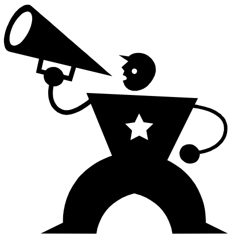

3. Les invitamos a participar en eventos y acciones de abril 2009:
   - Orientación para miembros (nuevos y regresando)........................8 de abril – (lugar y tiempo será anunciado)
   - Reunión comunitaria...........................14 de abril – en la Iglesia de San Marcos 6:00 PM
   - Demanda legal de Chevron................22 de abril – en Martinez (tiempo será anunciado)
   - "Ama a Tu Mamá" Celebración de Día de la tierra..........25 de abril en el Centro de Recreación "Tassafaronga" y la Escuela Primaria ACORN/Woodland en Oakland del Este de 11am a 6pm

4. Habla en contra del boletín técnico 604 (TB 604)
   This standard would regulate the flammability of filled bed clothing including comforters, mattress pads, and pillows. It does not require any health or environmental information about the chemicals and materials that will be used to meet the regulations. Contact Laura Zuniga, Chief, Bureau of Home Furnishings and Thermal Insulation, at bhfti @dca.ca.gov, (916) 574-2041 to say you don't want a bed clothing flammability standard that could lead to toxics in your bed. Go to http://www.bhfti.ca.gov/industry/tb604.shtml to learn about public hearing notice for TB 604.

CBE

APPENDIX

E. CALIFORNIA HOUSEHOLD EXPOSURE STUDY INTERVIEW

Report-Back Interview

Questions for Study Participants in the Richmond/Bolinas Household Exposure Study

Participant #: Date:
Interviewer(s):
Others present at time of interview:

Hello, I'm <name> from <affiliation>. Thank you so much for letting me come talk with you today about your experiences participating in the Household Exposure Study. Before we get started with the interview, I want to review what this study is about and ask you to sign this informed consent statement that says you agree to participate in the study. [*HAND DOCUMENT*] Let me just go over the form with you.

Informed Consent:

This study is designed to follow up on the sampling of air and dust from your home. The interview today will help us learn how people understand and respond to the laboratory results about chemicals that were detected in their home. We hope to use this information to improve how we report results to study participants, especially when the reports tell people results from their own home. We are also trying to learn how participants may use the information we give them.

The study is being done by Silent Spring Institute [*AS NEEDED, ADD*, a non-profit environmental health organization], Brown University, UC Berkeley, and Communities for a Better Environment [*AS NEEDED, ADD*, a non-profit environmental health and justice organization]. The National Institute of Environmental Health Sciences and the National Science Foundation are funding the research.

Back when we came to collect samples of air and dust from your home, you gave us permission to re-contact you. Then we gave you the results for what chemicals were found in your home and other homes in the study, and a researcher may have come to review your results with you and answer questions.

Now, if you agree, we would like to interview you about your response to how we reported to you what chemicals we found in your home. The interview will take about an hour. The questions are designed to help us evaluate how well we did in informing study participants about what was found in their homes and also to find out what people think was important about their

results. With your permission, we will tape record the interview to ensure that we get the most complete record of your responses.

All information that could identify you will remain confidential to the full extent of the law. The interview tape and transcript will be identified with a number rather than your name when they are used to write up reports. Access to your personal records will be restricted to researchers involved in this study.

There are no foreseeable risks to participating in this interview. There are no financial costs or benefits to participating.

The study will benefit people in Richmond, Bolinas, and elsewhere by helping us learn more about methods for effectively reporting to study participants on their individual household exposures.

Participation in the study is voluntary. You can refuse to participate in this interview or withdraw at any time. If you do not participate, there will be no penalty or loss of benefits to which you are otherwise entitled. If you withdraw, information gathered from the interview will be destroyed.

A summary of what we learn in this study will be reported in community meetings, the news media, and scientific journals. We will never use your name or other identifying information in any meetings, reports or articles.

If you are willing to participate in the interview, please sign this form giving permission for me to ask you the interview questions.

[*COLLECT SIGNED CONSENT FORM AND THANK PARTICIPANT. OR, IF PARTICIPANT DOES NOT CONSENT, THANK THEM FOR THEIR TIME.*]

Interview Questions

I. Participation

First, I want to ask about when you first participated in collecting the samples for the study. Researchers came to your home to collect air and dust samples and interview you about some products you use and activities you do in and around your home.

1. Thinking back to when a researcher first came to your home to collect the air and dust samples. What was that like?
2. Tell me about having the researcher interview you about things you do at home, like using pesticides or cleaning products and so on. What was that like?

II. Interpreting and Understanding Individual Results

Next I want to ask about when you received the results for your home. Recently, the study team sent results to individual participants in the household sampling. This information packet was followed up by a home visit from a researcher to go over the results in person with you. In my next questions, I'm going to ask about what we found inside your house [*FOR HOMES WITH OUTDOOR AIR SAMPLES, ADD…*] and then about what we found in the outside air.

3. What were your thoughts or feelings the first time you read the information packet that was mailed to you with your household sampling results?
4. Do you recall what the study found in your home?
   a. Do you remember any specific chemicals that were found in your home? *Probe for names, if they can remember them.*
   b. Would you say that the levels of any chemicals found in your home were "high"?
      i. Do you think the results seem high compared to what was found in other people's homes? Which chemicals were you thinking about? What makes you think that?
      ii. Do you think the results seem high compared to government guidelines? Which chemicals were you thinking about? What makes you think that?
   c. Were you surprised by the results? *If so, probe*:
      -What was surprising?
   d. Are you concerned about any possible health implications for you and your family from the chemicals found in your home?
   e. Did you find the conversation with the researcher useful? *If so, probe*:
      i. What was useful about that?
5. After receiving your results letter, did you contact Silent Spring or Communities for a Better Environment to learn more about the chemicals found in your home? *If so, probe*:
   a. What questions did you ask Silent Spring Institute or Communities for a Better Environment?
   b. Were they able to answer your questions? Were you satisfied with the response?
6. Were the sampling results presented in a useful format for you to learn about what chemicals were found in your home?
   Probe: Please describe what was or was not useful (or understandable).
7. Have you discussed the results with anyone? *If so, probe*:
   a. With whom?
   b. What did you discuss?
   If they say no, gently probe: perhaps your husband (if appropriate), one of your children, a neighbor, or friend…?
8. Did you contact a physician, nurse, or public health professional? *If so, probe*:
   a. What questions did you ask?
   b. What was his/her response?
9. Do you think the study tells you something about your health or the health of other family members?
   a. …for past health issues that have come up in your family?

b. …for the future health of your family?

10. Are you glad to have learned about the results for your own home?
    Probe: Why? Why not?

III. Reading & Interpreting General/Study Results

11. [*FOR HOMES WITH AN OUTDOOR AIR SAMPLE ONLY*] Do you recall what the study found in the outdoor air near your home?
    a. Do you remember any specific chemicals that were found in your outdoor air sample? *Probe for names, if they can remember them.*
    b. Would you say that the levels of any chemicals found in your outdoor air were "high"?
        i. Do you think the results seem high compared to what was found in other people's outdoor air samples? Which chemicals were you thinking about? What makes you think that?
        ii. Do you think the results seem high compared to government guidelines? Which chemicals were you thinking about? What makes you think that?
    c. Were you surprised by the results? *If so, probe*:
        -What was surprising?
    d. Are you concerned about any possible health implications for you and your family from the chemicals found in the outdoor air near your home?

12. So far, we've talked about what the study found in your home [*FOR HOMES WITH OUTDOOR AIR SAMPLE, ADD…*] and in outdoor air nearby,
    now let's talk about what the study found overall about other homes in Richmond and Bolinas.
    a. What did you notice about the results for the other homes in the study?
    b. Are there results for specific chemicals that you think are important?
    c. Were you surprised by anything in the results? What was surprising?

13. The report packet with your results included a graph that compares indoor and outdoor air levels across Richmond and Bolinas homes. Did you see that?
    a. What did you think about the similarities and differences between the two communities? *Probe if person says the communities are different:* Why do you think these two communities are different?
    b. Were you surprised by the results? *If so, probe*:
        -What was surprising?

14. If you were asked to describe to family, friends, and neighbors what you learned from the study, what would you tell them?

15. Did you attend any community meetings about the study?
    a. Was this a useful format for you to get information about the study? Why or why not?

[ASK QUESTION 16 IF INTERVIEWS TAKE PLACE AFTER A PUBLIC MEETING OR NEWS MEDIA COVERAGE. OTHERWISE, THEY WILL BE OMITTED.]

16. , How else did you hear about the overall study results?
    a. Through the television, radio, or newspapers?
        If so, probe: Which ones?

IV. General Implications of Study

17. Did the study change your thinking about what affects the air and environment inside your home?
    If yes, probe:
    a. Can you tell me how it changed your thinking?

18. Did the study results change your thinking about outdoor air in your community? *If yes, probe:*
    a. Can you tell me how it changed your thinking?

19. Have you considered making any changes in your home to reduce indoor chemical exposures as a result of the study?
    If yes, probe:
    a.. Can you tell me about any changes you have made, or are planning to make?

    If no, prompt:
    b. Are there changes that you would like to make, but aren't sure what alternatives there are?
    c. Have you considered buying different products like cleaners or detergents?
    d. Have you considered community actions to reduce indoor chemical exposures as a result of the study?

20. Have you considered taking any actions to reduce the outdoor chemical levels near your home?
    If yes, probe:
    a. Can you tell me about any changes you have made, or are planning to make?
    If no, prompt:
    b. Are there changes that you would like to make, but aren't sure what alternatives there are?
    c. Have you considered community actions to reduce outdoor chemical exposures as a result of the study?

21. Did the study change your views about what role you think chemicals might play in disease or illness? *If so, probe*:
    a. In what ways?

22. As a result of this study, are there any changes that you would like to see in how companies manufacture, sell, or use chemicals? *If so, probe*:
    a. What changes would you like to see?

b. How do you think those changes can be made?

23. As a result of this study, are there any changes that you would like to see in how the government regulates chemicals and consumer products? *If so, probe:*
    a. What changes would you like to see?
    b. How do you think those changes can be made?

24. What additional resources or information would you like to have as a result of this study?

V. Final Thoughts

25. Anything else you'd like to comment on?

Thank you very much for your time and thoughtful participation in this study. Your input will be important for future follow-up on the results in this study and in other environmental health studies.

APPENDIX

F. COMMUNITY MEETING SURVEY: POST-FORUM QUESTIONNAIRE

Note: Your responses contribute to research conducted by Silent Spring Institute in partnership with Brown University (Providence, RI), University of California Berkeley (Berkeley, CA) and Communities for a Better Environment (Oakland, CA). This research is funded by the National Institute of Environmental Health Sciences and the National Science Foundation. Your responses will be kept confidential. We will not contact you about your comments or participation without your consent.

---

1. How did you learn about this event? (e.g., word-of-mouth, newspaper, flyer etc.)

---

2. Why did you attend, and what did you hope to get out of coming to this event?

---

3. What was the most important or useful information you learned tonight?

---

4. Do you have any unanswered questions or unaddressed concerns? If so, please briefly describe.

---

5. May we contact you for a more detailed follow up interview about your experience of this event?

Circle one: Yes No

If so, please print your contact information:

Name: Telephone:

Address: Email:

[Por favor, vea el otro lado para llenar esta encuesta en español.]

WHEN POLLUTION IS PERSONAL

HANDBOOK FOR REPORTING RESULTS TO PARTICIPANTS
IN BIOMONITORING AND PERSONAL EXPOSURE STUDIES

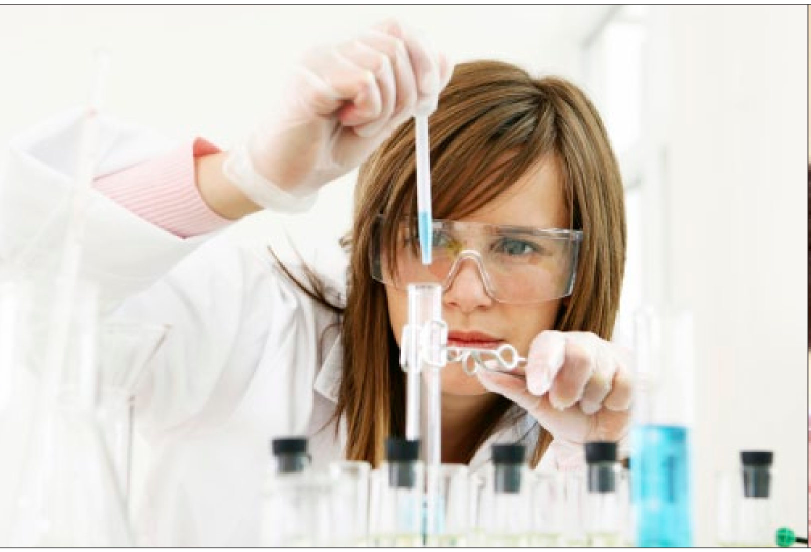

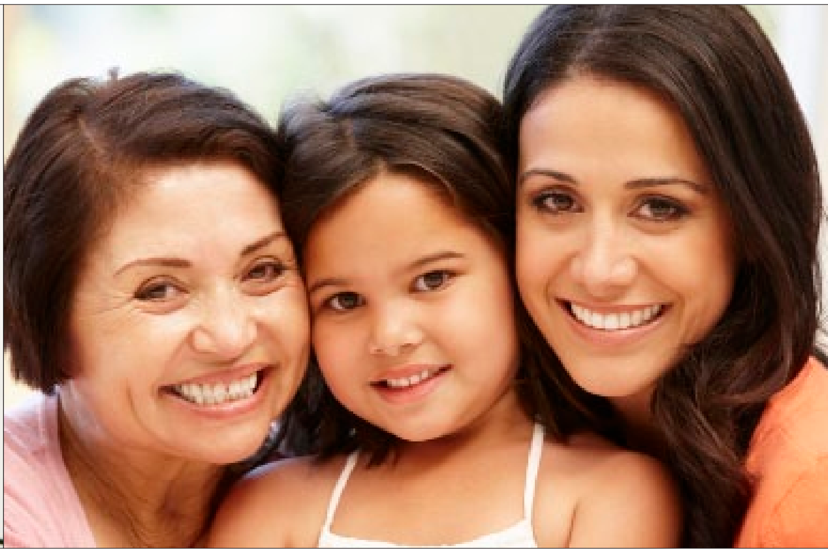

SILENT SPRING INSTITUTE

29 Crafts Street Newton, MA 02458 617-332-4288
www.silentspring.org
